# The Prevalence of Species and Strains in the Human Microbiome: A Resource for Experimental Efforts

**DOI:** 10.1371/journal.pone.0097279

**Published:** 2014-05-14

**Authors:** Laurens Kraal, Sahar Abubucker, Karthik Kota, Michael A. Fischbach, Makedonka Mitreva

**Affiliations:** 1 Department of Bioengineering and Therapeutic Sciences, University of California San Francisco, San Francisco, California, United States of America; 2 California Institute for Quantitative Biosciences, University of California San Francisco, San Francisco, California, United States of America; 3 The Genome Institute, Washington University School of Medicine, Saint Louis, Missouri, United States of America; 4 Department of Medicine, Washington University School of Medicine, Saint Louis, Missouri, United States of America; 5 Department of Genetics, Washington University School of Medicine, Saint Louis, Missouri, United States of America; University of Hyderabad, India

## Abstract

Experimental efforts to characterize the human microbiota often use bacterial strains that were chosen for historical rather than biological reasons. Here, we report an analysis of 380 whole-genome shotgun samples from 100 subjects from the NIH Human Microbiome Project. By mapping their reads to 1,751 reference genome sequences and analyzing the resulting relative strain abundance in each sample we present metrics and visualizations that can help identify strains of interest for experimentalists. We also show that approximately 14 strains of 10 species account for 80% of the mapped reads from a typical stool sample, indicating that the function of a community may not be irreducibly complex. Some of these strains account for >20% of the sequence reads in a subset of samples but are absent in others, a dichotomy that could underlie biological differences among subjects. These data should serve as an important strain selection resource for the community of researchers who take experimental approaches to studying the human microbiota.

## Introduction

A growing number of research groups use wet lab experimental approaches to study bacterial strains from the human microbiota [1]–[5]. Many of the most commonly studied strains have been selected for historical rather than biological reasons, raising the possibility that substantial research effort is being devoted to organisms that are neither associated with disease, nor widely or variably distributed in the human population.

Several approaches have been used to enumerate the composition of bacterial communities. These include 16 S rRNA sequencing [6]–[12]; methods that utilize protein-coding markers [13]; single-copy, single-marker complements to 16 S such as *rpoB* [14]; and interspace typing [15] and shotgun metagenomic sequencing [16], [17], [9], [10], [18], [8], [19], [20].

However, so far no global overview of abundance on the species or strain level has been presented, making it difficult for experimental groups to select strains to study on the basis of abundance data in human subjects. In this study we took advantage of the subset of the samples for which whole-genome shotgun (WGS) metagenomic sequencing was performed, providing an opportunity to determine at the lowest taxonomic level which bacterial strains are broadly distributed among healthy subjects and which ones vary widely from one subject to the next.

To study the pattern of strain distribution in the human microbiota and to create a resource to guide strain selection for experimental characterization, we systematically mapped reads from 380 WGS sequencing samples from the Human Microbiome Project (HMP) [9], [10] to complete or draft sequences from 1,751 reference genomes selected by the HMP; 844 of the reference genomes recruited reads from our data set. Our samples, which came from 100 healthy subjects, cover six major sample sites: stool (representing the lower gastrointestinal tract), tongue dorsum (upper surface of the tongue), buccal mucosa (cheek), supragingival plaque (tooth biofilm above the gum line), posterior fornix (the larger recess of the vagina, behind the cervix), and anterior nares (nostrils) as these are the sites with the most samples available. Species of the human microbiota have been studied for decades, and the model species have been chosen based on culturability and genetic tractability, among other characteristics. However, our analysis of whole-genome shotgun metagenomic data from the Human Microbiome Project [9], [21], suggests that the most abundant strains in the microbiome of healthy individuals are highly understudied, suggesting a needed shift in selecting the laboratory strains used for experimental studies of the human microbiota.

## Results and Discussion

### The methodology and its limitations

Several methodologies have been developed for the phylogenetic assignment of whole-genome shotgun metagenomic sequence data. These include clade-specific markers to unambiguously assign reads to microbial clades [18], a sequence compositional classifier based on the structured output paradigm [22], a hybrid, rank-specific classifier based on BLAST and Naïve Bayes [23], and a method that combines a NGS aligner with an optimized mapping strategy, appropriate parameters and the removal of genomes with high similarity from the database of reference genomes [24]. Our analysis took advantage of the last of these techniques, which has been evaluated previously and was used to taxonomically classify 22.4 billion 100 bp microbial Illumina reads (after subtraction of the human reads) originating from 380 samples from 6 body sites; this method has a very low percentage of incorrectly classified reads (33% at the strain level and just 0.0003% at the species level) [24]. The WGS metagenomic reads were aligned to a reference genome database containing 1,751 bacterial genomes representing 1,253 species [24]. Overall, 57% of the reads could be mapped to a reference genome, ranging from 33% for the anterior nares to 77% for the posterior fornix.

The ‘top random’ mapping strategy of the aligner (in the case of multiple equally high scoring top hits the aligner randomly reports one hit used to map sequencing reads [24]) has two important drawbacks, both of which arise from the uneven taxonomic distribution of reference strains in the reference genome database (i.e., some clades have more sequenced strains than others). First, it has difficulty distinguishing closely related strains of the same species, since reads that map to highly conserved genomic regions are assigned randomly to one of the strains. Second, species with a single strain in the reference database appear more abundant on the strain level than species with multiple strains, since reads are often divided among strains in the latter case. To make the limited but important effect of these drawbacks easy to understand, we ran two of our analyses at the species level in addition to the strain level to facilitate a direct comparison (Figures S5, Figure S6 and Table S2). An obvious limitation of using reference genomes is that the actual strains present in the subjects will often differ from the reference strain. However, the reads will be recruited to the closest genome in the database and the reference genomes have been chosen to be representative of the human microbiome [21], [9], [10]. The reference strains will thus be a great starting point for experimentalists.

**Figure 1:**
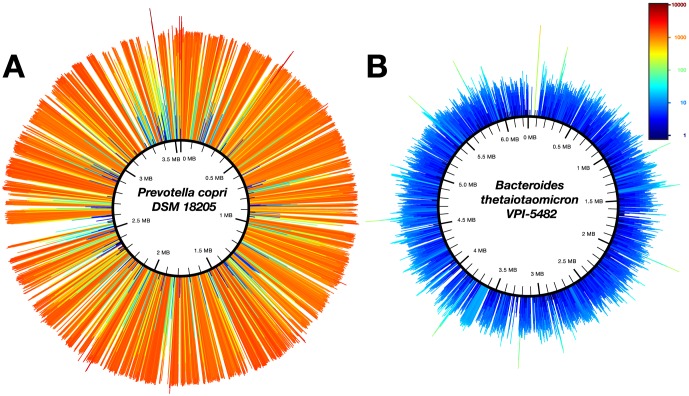
Read recruitment for strain-level abundance inference. Read recruitment of two genomes: one that is highly abundant and one with an average abundance. Each peak represents the average coverage for a 1000 bp bin both in color and length (on a log scale). (A) The breadth and depth of sequencing coverage for *Prevotella copri* DSM 18205 in stool sample SRS017307. For this sample the reads cover 75.3% of the genome with an average coverage of 1006.2 resulting in a relative abundance of 67% in this sample (Figure S2: Stool). The genome of *P. copri* DSM 18205 is a draft comprising 27 contigs with an unknown order (ordered randomly in this figure). (B) The breadth and depth of sequencing coverage for *Bacteroides thetaiotaomicron* VPI-5482 in stool sample SRS019397. *B. thetaiotaomicron* VPI-5842 has an average depth of 7.6 for 73.6% of the genome, resulting in a relative abundance of 0.5% in this sample (Figure S2: Stool).

The abundance of the reference strains in each sample was estimated by calculating the product of its breadth (defined as the percent of covered bases over the length of the reference genome) and depth (defined as sum of depths of each covered base divided by the length of the genome) of coverage (Figure 1, Figure S1, Table S1 and Table S4) [8]. Our metric was chosen to represent the probability of a genomic fragment to be sequenced in a sample. The abundance of a strain was divided by the total abundance of that sample to obtain the relative abundance of each strain in each sample. We represent these data as a series of heat maps showing the calculated relative abundance of each reference strain in the samples from our set (Figures S2, S3, S4 and S5). To inspect whether the coverage maps generated by this method show a reasonably even depth across most of the genome, we visually inspected three coverage maps for each of the 30 strains discussed below: one each for the samples in which the strain has the maximum, minimum, and median breadth of coverage (Figure S1B). While the map for the minimum coverage samples is often sparse, the maps for the median coverage samples generally appear even.

It is important to emphasize that the breadth of coverage is never 100% (the highest we found was 99.63%), because we are mapping the reads to reference strains whose genome sequences will differ from that of the organism in the sample and serve as a related (but not identical) stand-in in our analyses. Because we do not report strains with a depth of coverage below 1%, the reported relative abundance will differ by at most two orders of magnitude. However, reference strains with a low breadth but high depth of coverage most likely represent genomic fragments that are missing from the reference strains that otherwise best match the actual strains present in the sample and can lead to an overestimate of the number of total strains present in a sample. Conversely, the relative abundance of strains with a high depth and breadth of coverage will likely be underestimated. The number of strains in a sample can also be underestimated. For example, two strains that differ from each other but are both most similar to one strain in the reference genome database will be counted as one strain with the combined abundance of the both strains that are actual present.

The HMP dataset consists of 18 sample sites, but most of them comprise only a handful of subjects and have been excluded from our analysis. While the analyses described below cover six sample sites that have a minimum of 33 subjects, we have centered our discussion around the stool since, to date, experimental efforts on the human microbiota have focused predominantly on the gut community.

### Whole-genome shotgun data reveal strain-level details obscured by 16 S analysis

**Figure 2:**
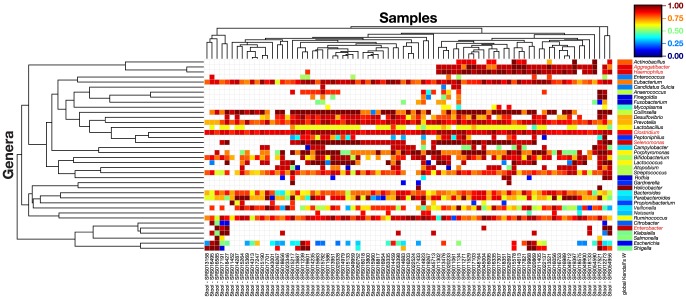
Samples that look similar by 16 S can be very different at the species level. For each 16(OTU), we calculated an average rank order of strain abundance in the WGS data and quantified the degree of concordance between the observed rank order in each sample and the average rank order (Kendall's coefficient of concordance, *W*). Kendall's *W* ranges from 0 (no agreement) to 1 (full agreement). The heat map shows Kendall's *W* for each OTU in each sample, and results are hierarchically clustered using Spearman rank correlation with average linkage. The overall concordance of all the samples is shown in the far right column. For most genera such as *Bacteroides*, the rank order of strains within the genus is highly variable among samples, suggesting that our WGS analysis provides much additional information beyond what can be deduced from the 16 S data. Genera with a high, statistically significant Kendall's *W* (>0.8) are colored red. For these 5 of the 38 total genera, 16 S data can be used to infer the rank order of abundance of strains within the genus. For the remaining genera, WGS data is essential to determine which are the dominant strains in each sample.

Several groups have reported on the agreement of metagenomic and 16 S data [25], [18], [26]. However, the additional information provided by the whole-genome shotgun (WGS) data as compared to the 16 S data (Figure S7A–G, Table S3) as it pertains to the composition of strains in a sample has not yet been explored quantitatively. Does WGS allow us to determine which specific strains are present in a sample or is this composition so invariable as that it can be deduced from 16 S data? To answer this question, we calculated an average rank order of strain abundance for each 16 S operational taxonomic unit (OTU) in the WGS data and quantified the degree of difference between the observed rank order and the average rank order (Figures 2 and S7H–L). For most genera such as *Bacteroides*, the rank order of strains within the genus is highly variable among samples, suggesting that a WGS analysis provides much additional information beyond what can be deduced from the 16 S data. For example, 16 of the stool samples have *Bacteroides stercoris* ATCC 43183 as the most abundant strain in the genus, while another 10 have *Bacteroides vulgatus* PC510 as the most abundant strain. These two strains share a core set of 2585 genes, while 1192 are unique to *B. stercoris* ATCC 43183 and 1371 are unique to *B. vulgatus* PC510 (as compared using the Integral Microbial Genome (IMG) Platform (http://img.jgi.doe.gov/) [27]). Communities dominated by different species of *Bacteroides* may therefore exhibit functional differences, so the ability to distinguish them using WGS data will likely prove important.

Interestingly, for a few genera such as *Clostridium*, the rank order of strain abundance varies little from sample to sample, suggesting that the *Clostridium* community composition may be unusually invariant (Figure 2). For these genera, our WGS data provide little additional information since the rank order of strain abundance could be estimated accurately from 16 S data by assuming that it matches the average.

### Strains with an unusually variable distribution pattern

**Figure 3:**
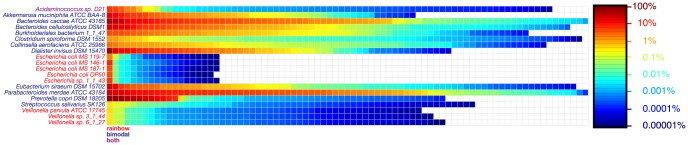
A subset of strains have unusually variable numbers across stool samples. Strains that are considered variable by the rainbow and/or bimodality metrics are ordered by their relative abundance across the sample set. Rainbow strains are outliers (1.5× higher than the interquartile range) in their coefficient of variation across all samples (Figure S8A). Bimodal strains are defined as strains that contain two groups of at least 5 samples that vary less than 2 orders of magnitude in their abundance within the group, but over 3 orders of magnitude between the two groups. One strain, *Acidaminococcus sp*. D21, is considered variable by both metrics in stool.

One striking result of our analysis is the detection of strains with unusual patterns of distribution, which could make these organisms intriguing candidates for future experimental study. Here, we focus on strains whose levels are unusually variable across our sample set. We used two methods to identify these strains: the first involved constructing a coefficient of variation distribution and identifying its outliers (Figure S8A), and the second sought to find strains whose rank order of abundance across the samples was bimodal (see methods). Any effect such a strain has on host physiology would be prominent in some people and nearly absent in others; thus, strains with highly variable distribution patterns could reveal bases for microbiota-linked variability in the human population (Figures 3, S8B and S9A–B).

We find that 12 of the 81 stool samples have a high abundance of *Prevotella copri* DSM 18205. While the distribution pattern of *Prevotella* was already known to be variable since the high abundance of *Prevotella* defines one of the recently described ‘enterotypes’ [28], our data show strikingly that the reference genome of *Prevotella copri* DSM 18205 recruits sequence reads from the WGS data in strong preference to the other 18 reference genomes of *Prevotella* represented in our database, suggesting that *P. copri* DSM 18205 is an ideal starting point for experiments seeking to characterize its properties within the gut community. Other reference strains with highly variable patterns of distribution come from a broad range of phyla, including members of the Bacteroidetes (*Bacteroides caccae* ATCC 43185, *Parabacteroides merdae* ATCC 43184) and Firmicutes (*Dialister invisus* DSM 15470, *Eubacterium siraeum* DSM 15702) as well as the Actinobacterium *Collinsella aerofaciens* ATCC 25986, the Verrucomicrobium *Akkermansia muciniphila* ATCC BAA-835, and the Proteobacterium *Burkholderiales bacterium* 1_1_47.

### The minimal microbiome and body site diversity

An emerging area of inquiry involves reductionist approaches to studying the function of the microbiota: how many strains are required to construct a minimal microbiome with key attributes of a more complex community [29]? This question is important for many experimental studies of the human microbiota, including those that use germ-free mice to probe the interplay among the microbiota, the host, and the diet [4], [30]–[32].

**Figure 4:**
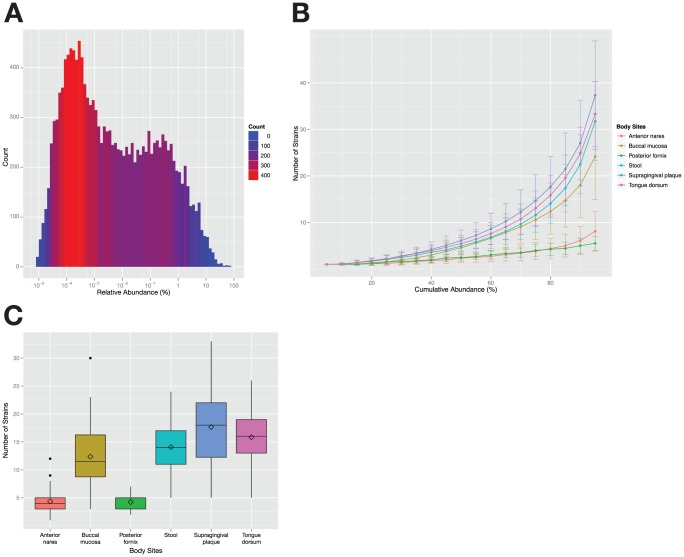
14 strains account for 80% of the cells in a typical stool sample. (A) The distribution of all the calculated relative abundances for each strain in each stool sample. The color and height of the bars represents the number of values that fall within this abundance bin. (B) Cumulative relative abundance of strains. This line graph shows for the 6 body sites how many strains on average it takes to constitute a percentage of the total abundance in a sample, ranging from 5% to 95%. The error bars represent the standard deviation. On average 125, 235, 115, 168, 373 and 376 strains are needed for 100% abundance in the anterior nares, buccal mucosa, posterior fornix, stool, supragingival plaque and tongue dorsum respectively. (C) The distribution of the number of strains needed for 80% abundance in the different body sites. Note that these distributions are similar to the distributions of diversity metrics (Figure S9A–B). The median is indicated by a horizontal line in the box (covering the 25th until the 75th percentile), and the diamond represents the average. The whiskers of the box are the lowest and highest observations of number of strains needed for 80% abundance.

**Figure 5:**
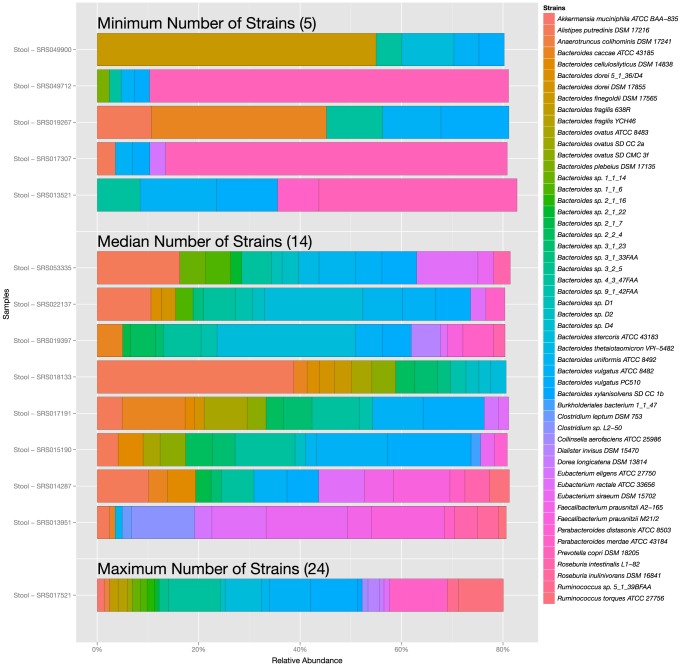
Stool samples can be dominated by a wide variety of bacterial species, including numerous *Bacteroides* species. The contribution of individual strains to samples that contain the minimum (5), maximum (24) and median (14) number of strains required for 80% abundance.

As a first step toward answering this question, we calculated the number of strains, in decreasing rank order of abundance, that together comprise 80% of the mapped reads from a sample (Figure 4A–C). Surprisingly, 14 strains account for 80% of the mapped reads in the average gut community, while for the oral communities of the cheek, tongue, and teeth, 12, 16 and 18 strains are needed respectively. As expected, the number is lower for samples from the anterior nares and posterior fornix (4). Among communities from the same body site, the 80% are sometimes few and sometimes many: in stool, samples range from a minimum of 5 to a maximum of 25 strains making up the 80% and in supragingival plaque, the range is 4–30 (Figure 5). Even in a community as complex as the gut, just 5 strains account for 50% of the reads in a typical sample (Figure 4C). If the analysis is performed at the species level instead of the strain level, the numbers are even more striking: 7.3 species account for 80% of the mapped reads in the average stool sample, and just 2.8 species account for 50% (Figure S6).

**Figure 6:**
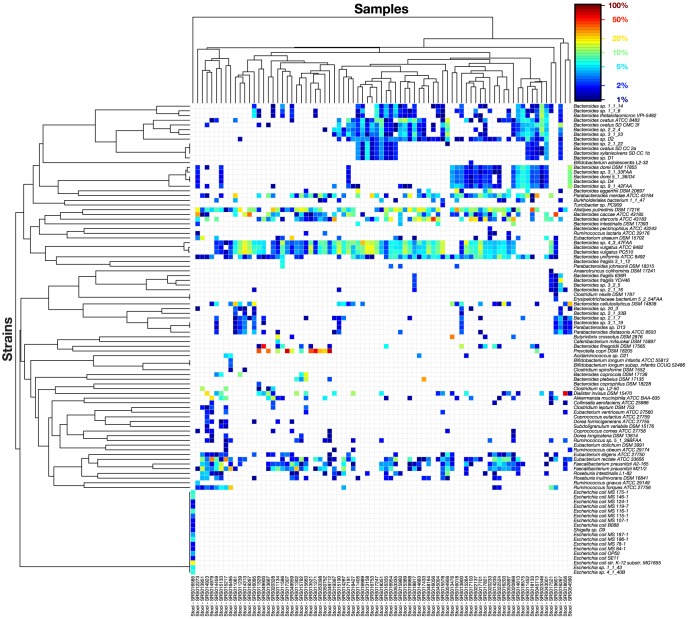
The distribution of strain abundances for the top 80% of each stool sample. To emphasize the differences in prevalence of the most abundant strains, strains are only shown if they make up 1% or more of the stool sample.

**Figure 7:**
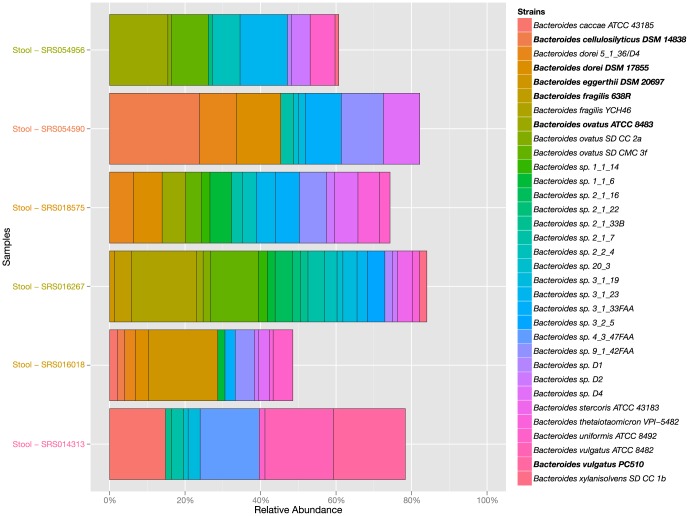
Six samples that are dominated by six different strains of *Bacteroides*. The contribution of 33 *Bacteroides* strains to the abundance of six samples are displayed in a bar chart. The six dominating *Bacteriodes* strains are *Bacteroides ovatus* ATCC 8483, *Bacteroides cellulosilyticus* DSM 14838, *Bacteroides dorei* DSM 17855, *Bacteroides fragilis* YCH46, *Bacteroides eggerthii* DSM 20697 and *Bacteroides vulgatus* PC510. Only strains with at least 1% relative abundance are plotted.

To determine the degree of variation among samples in their highly abundant strains, we constructed a heat map of strain abundance for the top 80% of mapped reads for each sample in the gut community (Figure 6); other communities are shown in Figure S9C–G. Strikingly, communities dominated by the same genus can differ quite dramatically at the species level. For example, two common configurations of *Bacteroides*-rich samples are dominated by *B. ovatus* or *B. vulgatus*, while rarer configurations feature *B. eggerthii*, *B. cellulosilyticus*, *B. fragilis*, or *B. dorei* as the most abundant strain (Figure 7). Notably, for a typical pair of *Bacteroides* species, one-third of the genes in each genome will be unique. Given the heterogeneity of *Bacteroides* species, a *B. vulgatus*-dominated community may well be as phenotypically different from a *B. ovatus*-dominated community as either one is from a *Prevotella*-dominated community. Since enterotypes are most usefully defined as communities with important phenotypic differences, our results indicate that there may be more enterotypes than are currently recognized, especially within *Bacteroides*-dominated communities.

Some strains are commonly found among the top 80%. For example, *Bacteroides vulgatus* ATCC 8482 was in the top 80% of 76.5% of stool samples [33], while four different strains – *Veillonella dispar* ATCC 17748, *Streptococcus parasanguinis* ATCC 15912, *Streptococcus salivarius* SK126, and *Prevotella melaninogenica* ATCC 25845 – were in the top 80% of 100%, 95.8%, 87.3% and 90.1% of tongue dorsum samples, respectively (Figure S9H). Other strains such as *Dialister invisus* DSM 15470 and *Bacteroides finegoldii* DSM 17565 are present less commonly, but when present, can dominate a community. Indeed, it appears surprisingly common to have a unique configuration of strains in the top 80%; more than a dozen diverse strains account for >20% of the abundance of just one (*Bacteroides plebeius* DSM 17135, *Eubacterium siraeum* DSM 15702, *Bacteroides finegoldii* DSM 17565, *Ruminococcus torques* ATCC 27756, *Butyrivibrio crossotus* DSM 2876, and *Bacteroides sp*. 4_3_47FAA), two (*Eubacterium rectale* ATCC 33656, *Bacteroides cellulosilyticus* DSM 14838, *Dialister invisus* DSM 15470 and *Faecalibacterium prausnitzii* M21/2) or three (*Alistipes putredinis* DSM 17216, *Bacteroides caccae* ATCC 43185, and *Parabacteroides merdae* ATCC 43184) out of the 81 samples. In other words, 23 out of 81 samples are dominated by 13 different strains, while the remaining 58 samples have a more even spread of strain abundances, where no one strain is present at >20% abundance.

A strain's abundance is not the ideal proxy for its importance; low-abundance members of a community can play key and very diverse roles in community function and host biology [34], [35]. Indeed, we could find strains that had a consistently low abundance across most of the sample set, indicating that they might play an important role in community function despite their low numbers (Figure S10A–C).

Nevertheless, it is reasonable to assume that the highly abundant strains are key players in community function. The fact that 14 strains can account for 80% of the reads in a sample (a proxy for cell density) suggests that the function of a gut or an oral community may not be irreducibly complex, and that experimental approaches involving synthetic communities of limited size will be highly instructive in discovering interspecies interactions important for community robustness and function [36]–[38].

### The most broadly distributed species have not been the focus of experimental study

**Figure 8:**
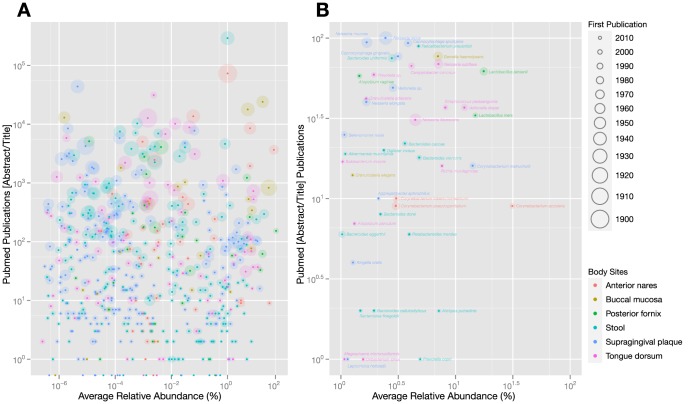
Some of the most abundant strains in the human microbiota are poorly studied. (A) Publications in PubMed versus abundance. The number of publications in PubMed [Abstract/Title] for each species (until April 2012) is plotted against the average abundance of each species. The year that the species first appeared in the abstract or title of an article in the PubMed database is indicated by the size of the circle. The color of the circle indicates the body site where the species is most abundant. (B) Expanded view of Publications in PubMed versus abundance. An expanded view of the lower-right corner of the scatterplot showing the species that have a high average relative abundance (top 100) but a low publication count (≤100).

A primary goal of the HMP is to enumerate the ‘normal’ human microbiota in a way that enables future studies of the key reference species, each of which are publicly available from the BEI repository (http://www.beiresources.org). To generate a crude estimate of how well the most broadly distributed species in our samples have been studied, we searched the PubMed database for the number of references that list the genus and species names of each strain in the title or abstract (Figure 8B). Surprisingly, of the 100 most abundant species, only 11 have more than 1000 publications; 64 have 100 or fewer publications. 44 of these 64 make up >1% of an average body site community (Figure 8B), including 13 common species from the gut. These numbers are remarkably small in comparison to the number of publications for common pathogens (*Staphylococcus aureus*, 73,134; *Pseudomonas aeruginosa*, 43,883) and model organisms (*Bacillus subtilis*, 20,362; *Escherichia coli*, 292,086). Importantly, the most well-studied species in this set had their genomes sequenced prior to the start of the HMP reference genome sequencing effort in 2007 [39], and in each case the number of publications rose quickly thereafter. Our heat maps of strain and species abundance will be a useful resource for guiding experimentalists to the key reference organisms for future study, including those with an unusually broad or variable distribution in the human population.

## Materials and Methods

### Data processing

The samples used in this study were part of the HMP project and were collected as previously described [40]. At the time of our analysis only 380 samples were available for the 6 body sites. Sequencing and analytical processing of the 16 S RNA and whole genome shotgun metagenomic data was done as previously described [9], [10]. Alignments to the reference genomes were made using a random top-hit strategy which involves reporting only a single, best hit per query, and in the case of a query having multiple equally strong best hits (i.e. mapping quality 0; [41]), one of those hits was chosen at random. The method also includes optimized parameters enabling accurate taxonomic assignments of WGS reads and a detection cutoff of 1% breadth and 0.01× depth of coverage [24]. The database of reference genomes used for taxonomic classification of the WGS reads has undergone a process of removing highly redundant bacterial strains [24]. In summary, i) the complete and draft genomes were categorized on a species level, resulting in categories that range from single strains to many strains per species; ii) For pairs of genomes with over 90% similarity on a genome-wide level based on genome-wide similarity at a pair-wise level, the genome that is longer and provides the most unique sequence was kept; iii) in the case of a large numbers of strains, a slightly relaxed homology (as low as 83%) was used and iv) bacterial strains that were collected from humans as part of the HMP were retained without being subject to redundancy removal, because these strains were deemed informative to profile human-associated microbiomes.

The breadth (defined as the percentage of covered bases over the length of the reference genome) and depth (defined as the sum of the depths of each covered base divided by the length of the genome) of coverage were calculated based on all alignments of each genome represented in the database using RefCov (http://gmt.genome.wustl.edu/gmt-refcov). To allow for the different number of input base pairs per sample and normalize across samples, the depth of coverage was normalized per 100 million base pair. In short, the average depth of coverage per reference genome was calculated using Refcov by taking the sum of the lengths of all the reads that align to that reference, divided by the total length of that reference. Because a single genome can span many references, the average coverage value was aggregated on a per genome basis. To obtain the normalized value, the total number of bases that mapped to the genome was divided by 100 million and applied to the average depth values as a modifier.

All data was analyzed using custom Python, R and Processing scripts and visualized using Processing (http://processing.org) and the ggplot2 R library (graphs, box plots and histograms) [42]. Matrix clustering was performed using Cluster [43] and visualized using TreeView [44].

### Calculating relative abundance based on whole genome shotgun metagenomic data

The abundance of each strain in each sample was estimated by calculating the product of its breadth and depth of coverage. The abundance of a strain was divided by the total abundance of that sample to obtain the relative abundance of each strain in each sample. Species-level estimates were calculated by taking the sum of the relative abundance of all the strains of a species in a sample.

### Whole-genome shotgun data reveal strain-level details obscured by 16 S analysis

For each genus that was represented by 3 or more strains in the whole genome shotgun data, Kendall's coefficient of concordance (*W*) [45] was calculated using the vegan R package. Kendall's *W* was used to determine the agreement of samples on the rank order of the strains in the genus with the average rank order of strains in the genus for all the samples. Genera with a significantly high (p<0.01 after multiple testing correction) Kendall's *W* (>0.8) were considered to be in agreement on the ordering of the strains in the genus.

### Strains with an unusually variable distribution pattern

Two metrics were used to identify strains that are highly variable among samples: Rainbow strains were defined by calculating the coefficient of variation of the relative abundance across all samples and identifying the strains that are outliers (1.5 times higher than the interquartile range) (Figure S8A). Bimodal strains contain two groups of at least 5 samples that vary over 3 orders of magnitude between the two groups, but vary less than 2 orders of magnitude in their abundance within the group.

### Top 80% by numbers

To determine the number of strains that are sufficient to accumulate a certain percentage of total abundance in a sample, the strains were ordered by their abundance and summed until the total constitutes a percentage of the total abundance in the sample. The number of strains was calculated for each sample and each body site allowing for the calculation of the mean and the standard deviation for each target abundance by percentage.

### PubMed analysis

PubMed data was obtained in April 2012. The PubMed database (http://www.ncbi.nlm.nih.gov/pubmed) was queried for the number of articles with the species name (no strain information was used) in either the title or the abstract using the [title/abstract] search tag. To identify the first publication with the species name in the abstract or title, the date of publication [DP] search tag was used with a decade as the date range.

## Supporting Information

Figure S1 **Genome coverage plots.** (A) Genome coverage for the 30 strains mentioned in the text visualized in a boxplot. The box indicates the 25^th^–75^th^ percentile, and the median coverage is indicated by a horizontal line in the box. The diamond represents the average and the outliers are visualized using dots. The whiskers of the box are the lowest and highest observation of coverage. (B) Read recruitment to the genomes of all the 30 strains mentioned in the text. For each strain, we show a coverage map for three subjects: the stool samples with the maximum, minimum and median coverage. Each peak represents the average coverage for a 100 bp bin both in color and length (on a log scale). All genomes are draft sequences, and consist of 3 contigs (*Akkermansia muciniphila ATCC BAA-835, Bacteroides vulgatus ATCC 8482* and *Eubacterium rectale ATCC 33656)* to 1575 contigs (*Bacteroides cellulosilyticus DSM 14838*). Here, the contigs from draft genome sequences are ordered by their average coverage (from high to low) as determined from the sample with the maximum coverage.
(PDF)

Figure S2 **Heat map of clustered strain abundance in individual body sites.** These figures show a heat map representation of the relative abundance of each strain (y-axis) for each sample (x-axis) for the 6 body sites individually as determined by depth X breadth of coverage (see Methods). The abundances are hierarchically clustered using Spearman rank correlation with average linkage.
(PDF)

Figure S3 **Heat maps of ordered strain abundance for all body sites combined.** A heat map representation of the relative abundance of each strain (y-axis) for each sample (x-axis) for the 6 body sites combined as determined by depth X breadth of coverage (see Methods). The abundances are hierarchically clustered using Spearman rank correlation with average linkage.
(PDF)

Figure S4 **Heat maps of ordered strain abundance.** Heat map visualization of the relative abundances with the samples ordered by the relative abundance for each strain. Species are listed alphabetically.
(PDF)

Figure S5 **Heat maps of ordered species abundance.** Heat map representation of the relative abundance of each species ordered by the relative abundance for each species (for each sample the abundances of strains within a species were summed to calculate the species total). Species are listed alphabetically.
(PDF)

Figure S6 **Top species by abundance.** This line graph shows how many species on average it takes to constitute a percentage of the total abundance in a sample, ranging from 5% to 95%. Strains were summed to calculate the total for each species, and the error bars represent the standard deviation. On average, 74, 154, 90, 92, 277 and 255 species are needed for 100% in the anterior nares, buccal mucosa, posterior fornix, stool, supragingival plaque and tongue dorsum, respectively.
(PDF)

Figure S7 **16 S heat maps and comparison with WGS data.** (A–G) These figures show a heat map representation of the relative abundance of each genus (y-axis) for each sample (x-axis) for six body sites, both individually (A–F) and together (G), as determined by 16 S sequence data. The abundances are hierarchically clustered using Spearman rank correlation with average linkage. (H–L) For each genus that was represented by 3 or more species in the whole genome shotgun data, the rank order for each sample was determined and compared to the average rank order of the genus using Kendall's *W*. The correlations are hierarchically clustered using Spearman rank correlation with average linkage. The overall concordance of all the samples is shown in the far right column. Genera with a high, statistically significant Kendall's *W* (>0.8) are colored red.
(PDF)

Figure S8 **Variability in strain abundance across samples.** (A) Distribution of the coefficient of variation (CV) for each strain across the samples for each individual body site. The distribution shows that the variation of the abundances of individual strains is the lowest in tongue dorsum and the highest in the posterior fornix (based on the median), while the distribution is the widest for the stool. The median diversity is indicated by a horizontal line in the box (covering the 25th until the 75th percentile), the diamond represents the average and the outliers are visualized using dots. The whiskers of the box are the lowest and highest observation of variation. The points that make up the distribution are plotted in the color of the body site. The points beyond the whiskers are considered outliers and define the rainbow strains (see methods). (B) Strains that show an unusually variable pattern across multiple samples based on two metrics. The samples are ordered by their relative abundance to show the variable distribution. The strains are color coded to indicate the metric by which they are considered to be unusually variable (red for rainbow strains, blue for bimodal strains, and purple for both metrics).
(PDF)

Figure S9 **Strain diversity and top 80% by abundance.** (A–B) Sample diversity (alpha diversity) visualized in a boxplot using the Simpson index (A) and the Shannon index (B) for each body site. The median diversity is indicated by a horizontal line in the box (covering the 25th until the 75th percentile), the diamond represents the average and the outliers are visualized using dots. The whiskers of the box are the lowest and highest observation of diversity. The Simpson index equals the probability that two strains taken at random from the data set are the same. A high Simpson index equals low diversity. Here the more common 1—Simpson Index is plotted. The Shannon index quantifies the uncertainty (entropy) in predicting the identity of a strain taken at random from the data set. A low Shannon index indicates low strain diversity. The Shannon index was calculated with *e* as the base of the logarithm. Low diversity can be caused either by a small number of total strains in the data set or by strain domination of the body site. High diversity indicates either a large number of total strains in the data set or a very even distribution of abundance of strains. (C–G) Heat map visualization of the distribution of abundances of the species that are part of the top 80% in at least one sample in the five remaining body sites (see Figure 6 for stool): (C) anterior nares, (D) buccal mucosa, (E) posterior fornix, (F) supragingival plaque and (G) tongue dorsum. Only abundances of 1% or more are visualized to emphasize the differences in prevalence of the most abundant strains. The values are hierarchically clustered using Spearman rank correlation with average linkage. (H) Strain participation in the top 80%. For all the strains that are in the top 80% in at least one sample in a body site the participation over all the samples is given as a percentage for each body site. The heat map of participation percentages is hierarchically clustered using Spearman rank correlation with average linkage.
(PDF)

Figure S10 **Stable low abundance strains.** To find the strains that are present stably at low abundance, strains were identified that differed by only two orders of magnitude over 90% of the subjects (including subjects where the strain was not present) for each of the six body sites. The upper limit of the relative abundance is 0.01% (A), 0.1% (B) and 1% (C), each excluding the strains found for the preceding condition. No stable low abundance strains were identified for anterior nares and posterior fornix. The stable low abundance strains that were identified are visualized in a heat map of the relative abundances with the subjects ordered by the relative abundance for each strain.
(PDF)

Table S1 **Relative abundance of reference strains for all body sites (WGS).** The abundance of each reference strain in each sample was estimated by calculating the product of its breadth and depth of coverage. The abundance of a strain was divided by the total abundance of that sample to obtain the relative abundance of each strain in each sample. The relative abundance is presented as a percentage.
(XLSX)

Table S2 **Relative abundance of species for all body sites (WGS).** Species-level estimates were calculated by taking the sum of the relative abundance of all the strains of a species in a sample. The relative abundance is presented as a percentage.
(XLSX)

Table S3 **Relative abundance of generas for all body sites (16 S).** The 16 S data was generated as previously described [9], [10]. The abundance of an OTU was divided by the total abundance of that sample to obtain the relative abundance of each OTU in each sample. The relative abundance is presented as a percentage.
(XLSX)

Table S4 **Breadth and depth of coverage of reference strains for all body sites (WGS).** The breadth and depth of coverage used to estimate the abundance of each reference strain in each sample. The breadth (defined as the percentage of covered bases over the length of the reference genome) and depth (defined as the sum of the depths of each covered base divided by the length of the genome) of coverage were calculated based on all alignments of each genome represented in the database using RefCov (http://gmt.genome.wustl.edu/gmt-refcov). The breadth of coverage is presented as a percentage.
(XLSX)
