# Supplementary material for: The Prevalence of Species and Strains in the Human Microbiome: A Resource for Experimental Efforts
Source: PLoS One. 2014 May 14;9(5):e97279. doi: 10.1371/journal.pone.0097279 (PMC4020798; doi:10.1371/journal.pone.0097279)

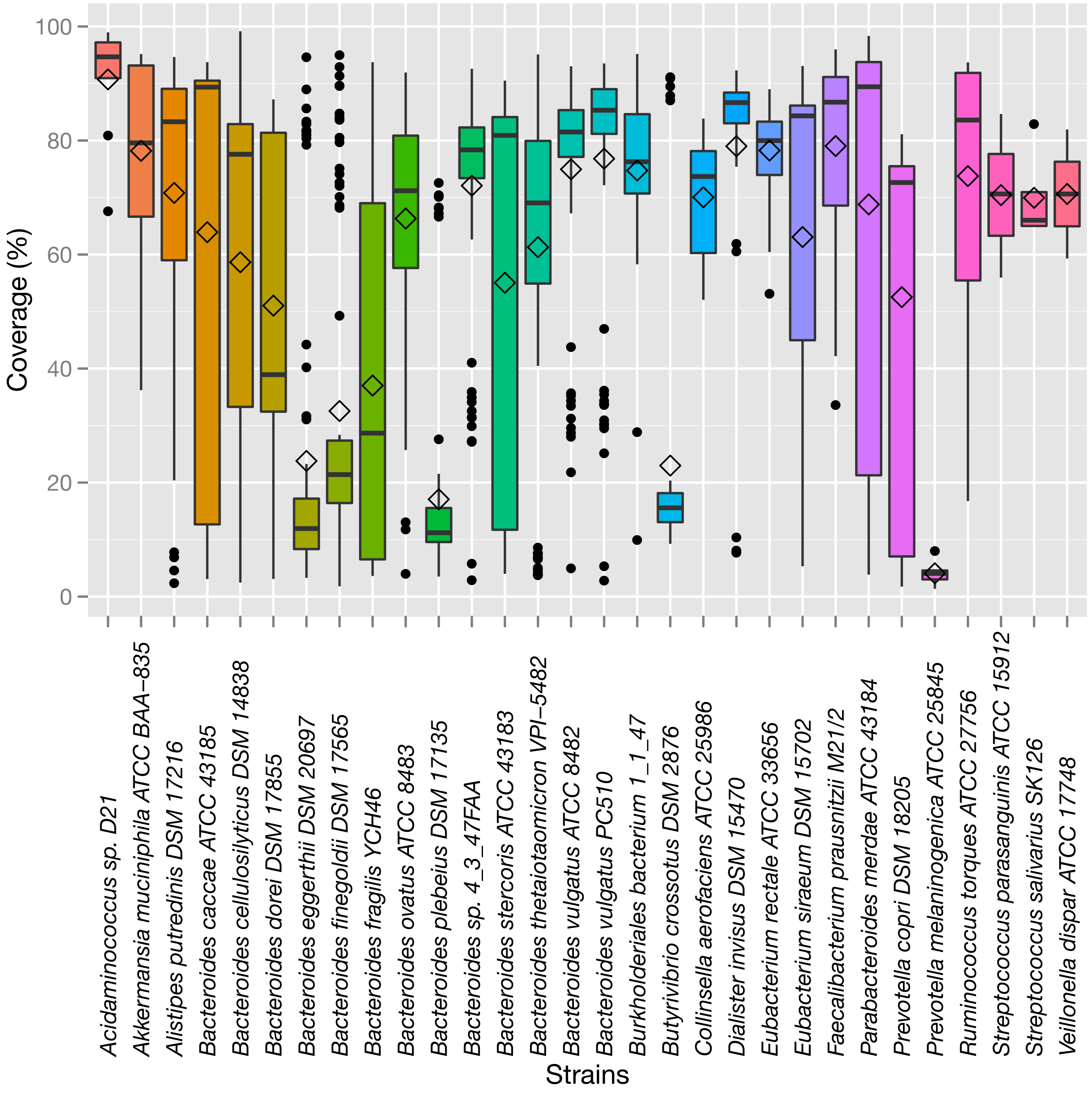
Coverage (%)
100
80
60
40
20
0
Acidaminococcus sp. D21
Akkermansia muciniphila ATCC BAA−835
Alistipes putredinis DSM 17216
Bacteroides caccae ATCC 43185
Bacteroides cellulosilyticus DSM 14838
Bacteroides dorei DSM 17855
Bacteroides eggerthii DSM 20697
Bacteroides finegoldii DSM 17565
Bacteroides fragilis YCH46
Bacteroides ovatus ATCC 8483
Bacteroides plebeius DSM 17135
Bacteroides sp. 4_3_47FAA
Bacteroides stercoris ATCC 43183
Bacteroides thetaiotaomicron VPI−5482
Bacteroides vulgatus ATCC 8482
Bacteroides vulgatus PC510
Burkholderiales bacterium 1_1_47
Butyrivibrio crossotus DSM 2876
Collinsella aerofaciens ATCC 25986
Dialister invisus DSM 15470
Eubacterium rectale ATCC 33656
Eubacterium siraeum DSM 15702
Faecalibacterium prausnitzii M21/2
Parabacteroides merdae ATCC 43184
Prevotella copri DSM 18205
Prevotella melaninogenica ATCC 25845
Ruminococcus torques ATCC 27756
Streptococcus parasanguinis ATCC 15912
Streptococcus salivarius SK126
Veillonella dispar ATCC 17748
Strains

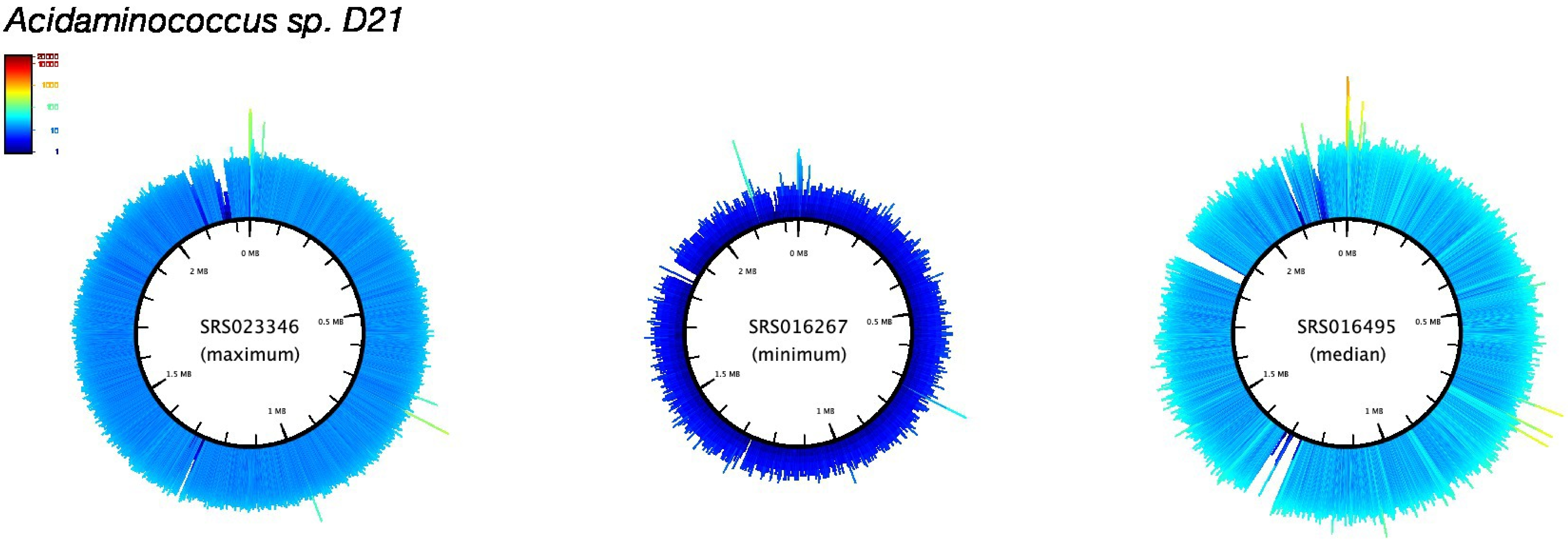

Acidaminococcus sp. D21
20000
10000
1000
100
10
1
0 MB
0.5 MB
1 MB
1.5 MB
2 MB
SRS023346
(maximum)
SRS016267
(minimum)
SRS016495
(median)

Akkermansia muciniphila ATCC BAA-835

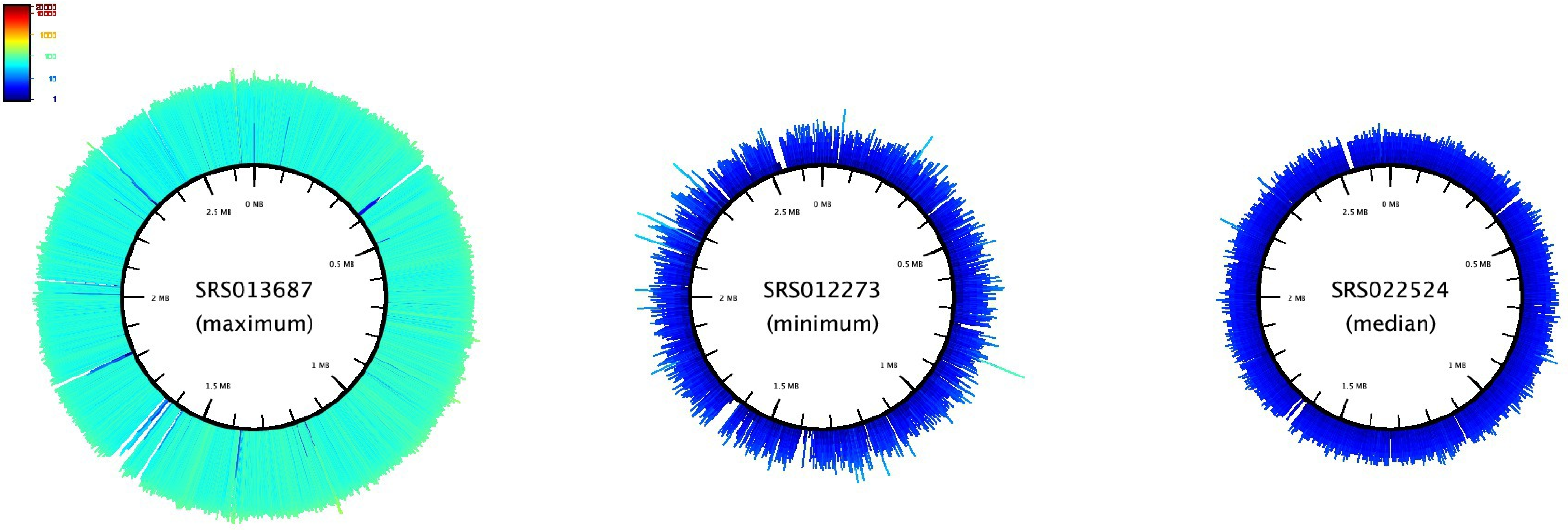

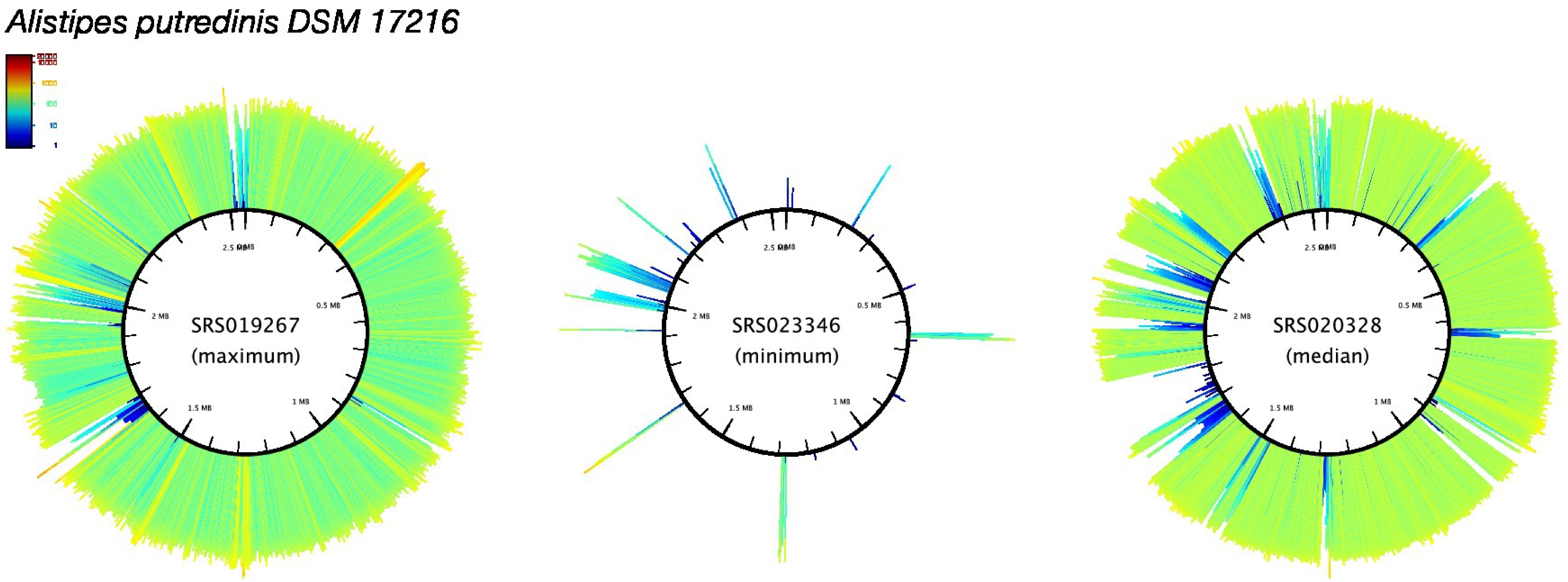

Alistipes putredinis DSM 17216
20000
10000
1000
100
10
1
SRS019267
(maximum)
SRS023346
(minimum)
SRS020328
(median)
0.5 MB
1 MB
1.5 MB
2 MB
2.5 MB

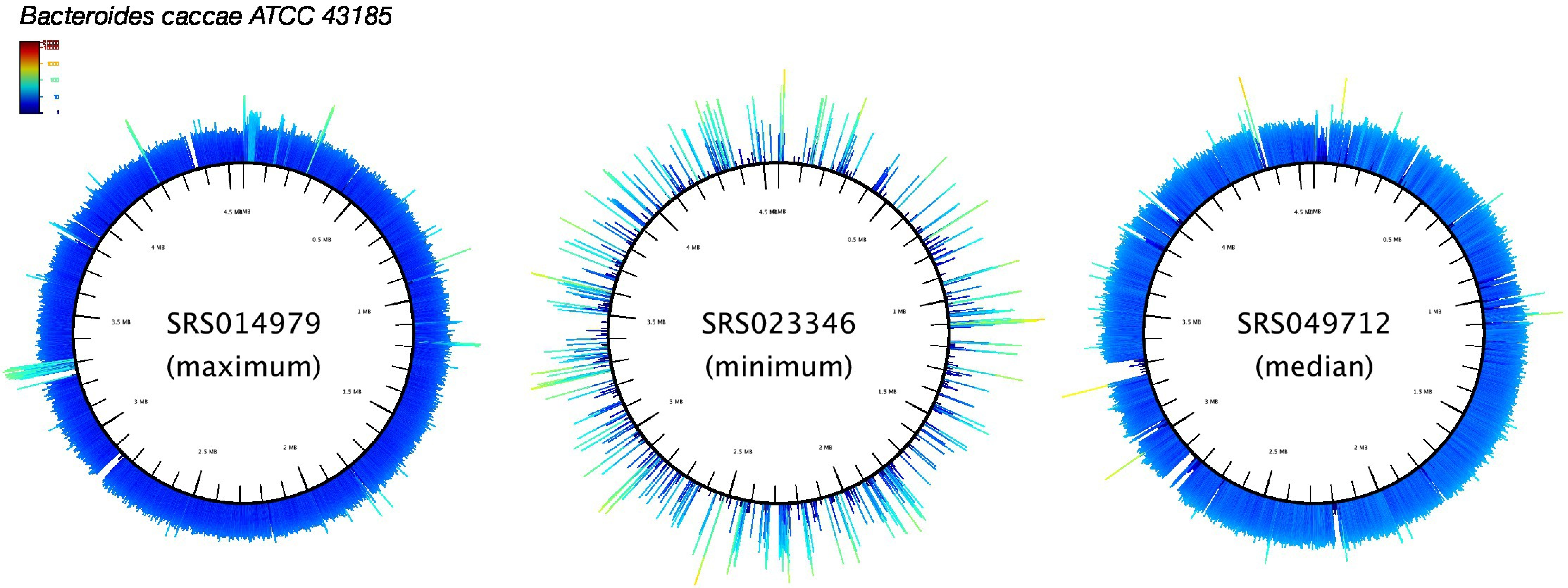
Bacteroides caccae ATCC 43185
SRS014979
(maximum)
SRS023346
(minimum)
SRS049712
(median)
4.5 MB
0.5 MB
4 MB
1 MB
3.5 MB
1.5 MB
3 MB
2 MB
2.5 MB

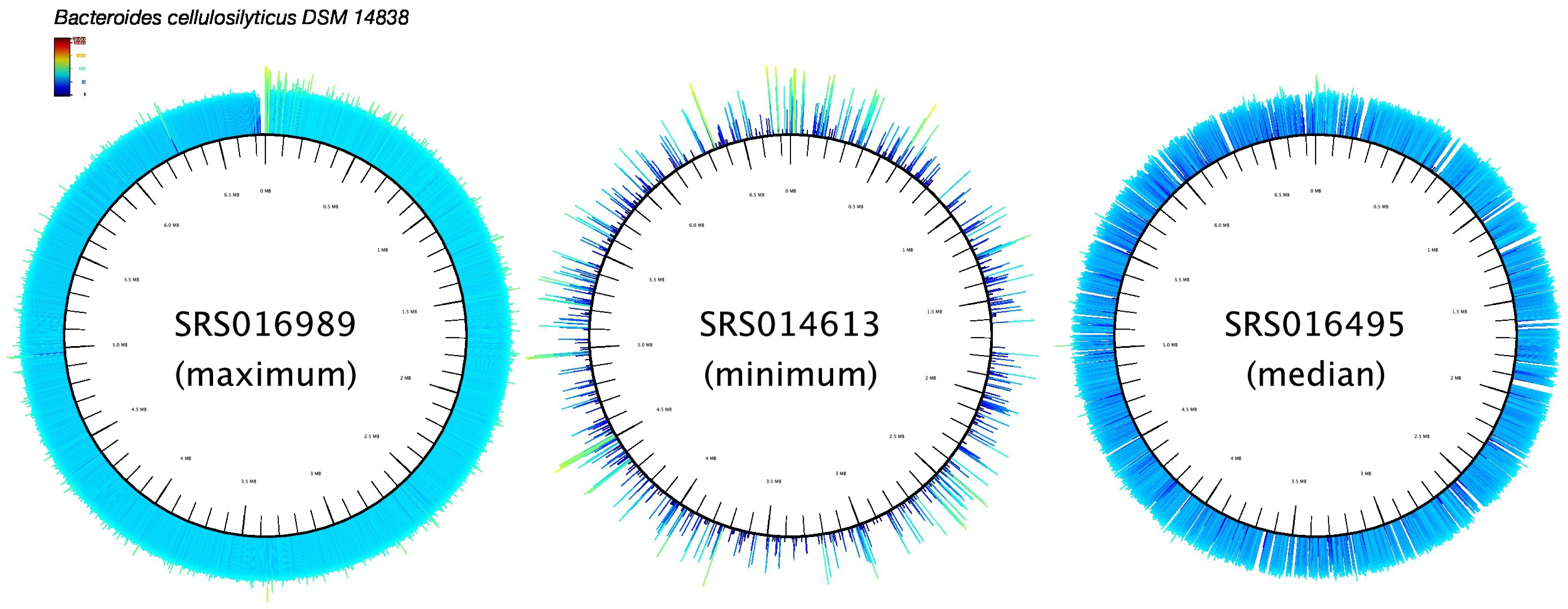

Bacteroides cellulosilyticus DSM 14838
SRS016989
(maximum)
SRS014613
(minimum)
SRS016495
(median)

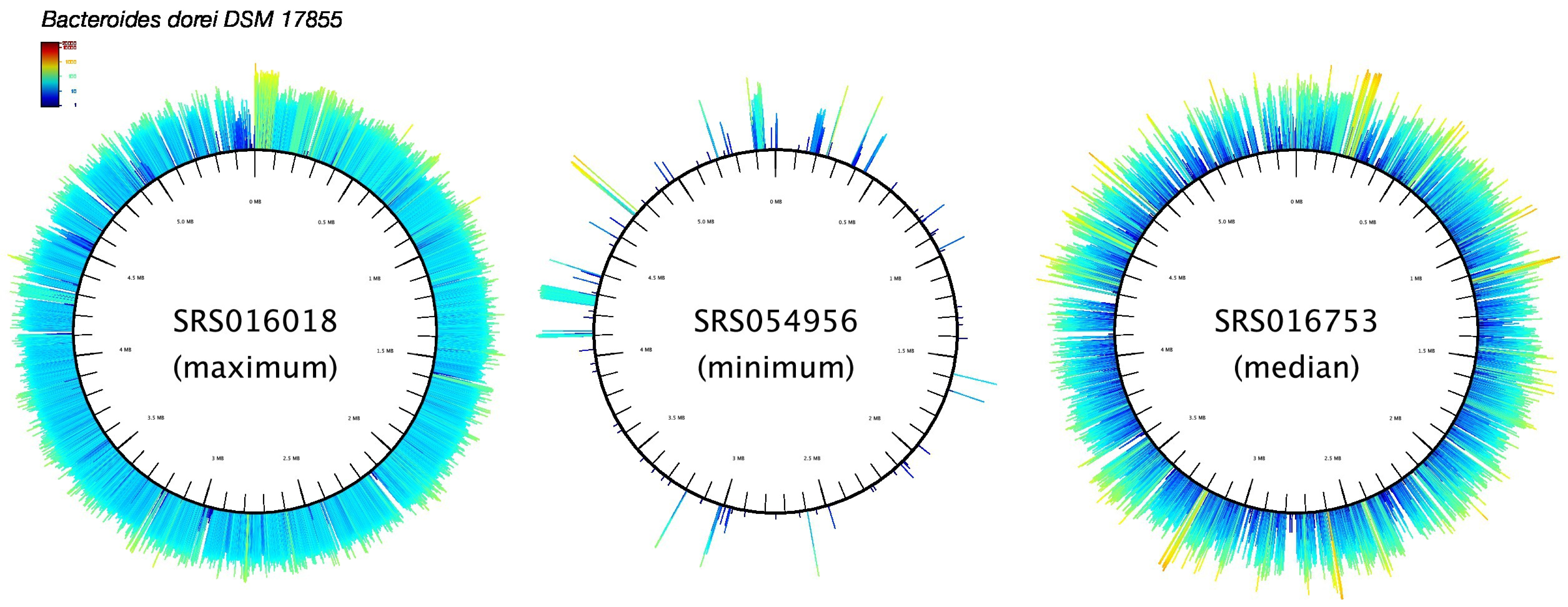

Bacteroides dorei DSM 17855
20000
10000
1000
100
10
1
0 MB
0.5 MB
1 MB
1.5 MB
2 MB
2.5 MB
3 MB
3.5 MB
4 MB
4.5 MB
5.0 MB
SRS016018
(maximum)
SRS054956
(minimum)
SRS016753
(median)

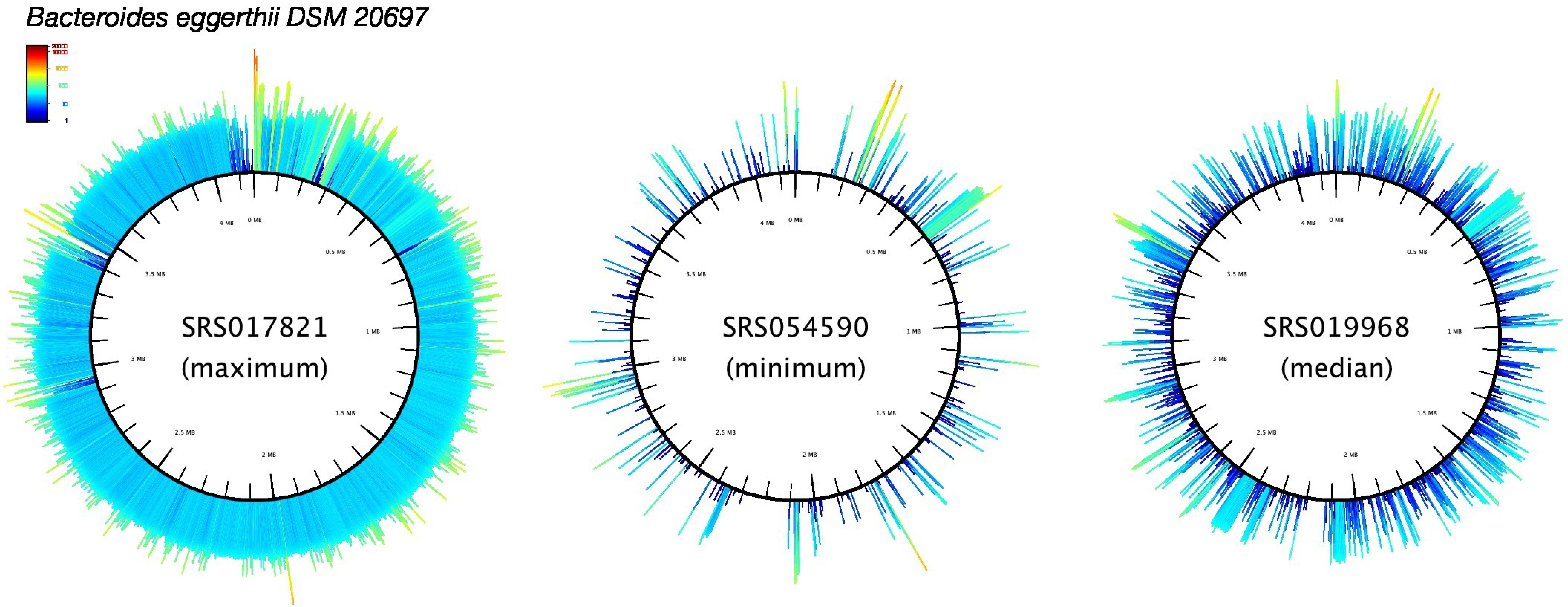
Bacteroides eggerthii DSM 20697
SRS017821
(maximum)
SRS054590
(minimum)
SRS019968
(median)
0 MB
0.5 MB
1 MB
1.5 MB
2 MB
2.5 MB
3 MB
3.5 MB
4 MB

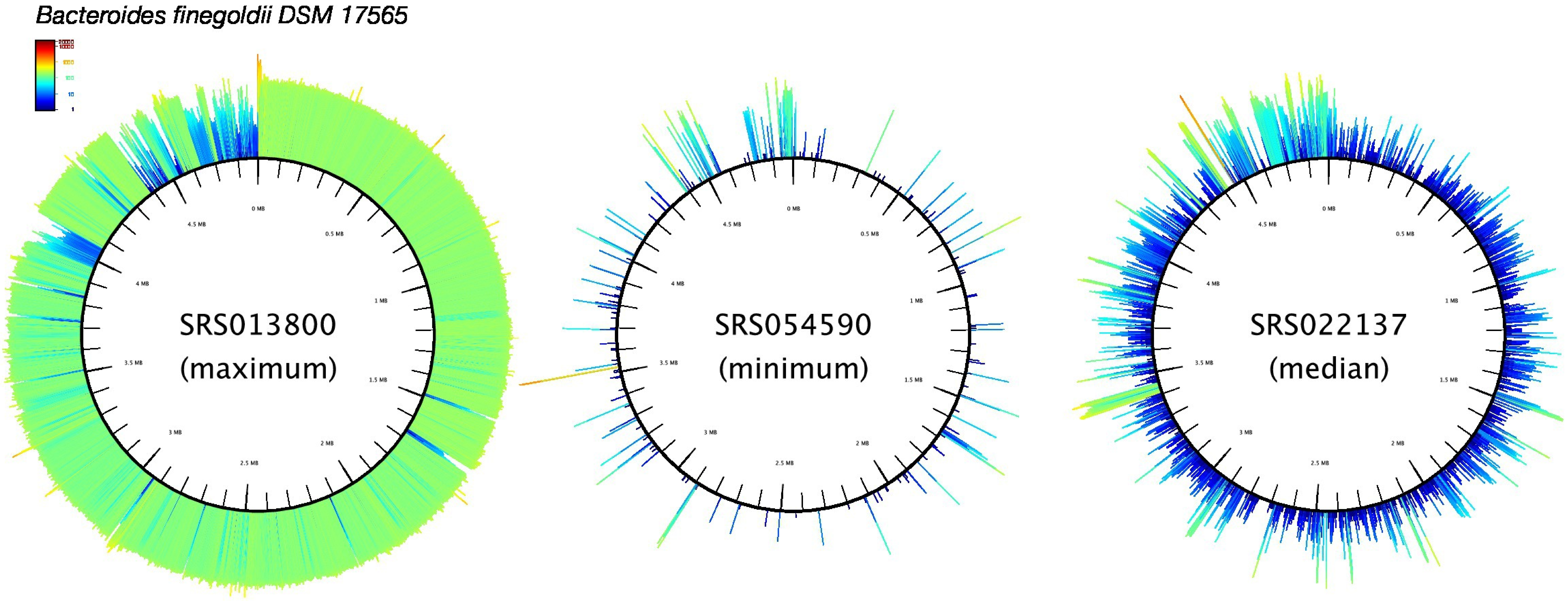

Bacteroides finegoldii DSM 17565
SRS013800
(maximum)
SRS054590
(minimum)
SRS022137
(median)

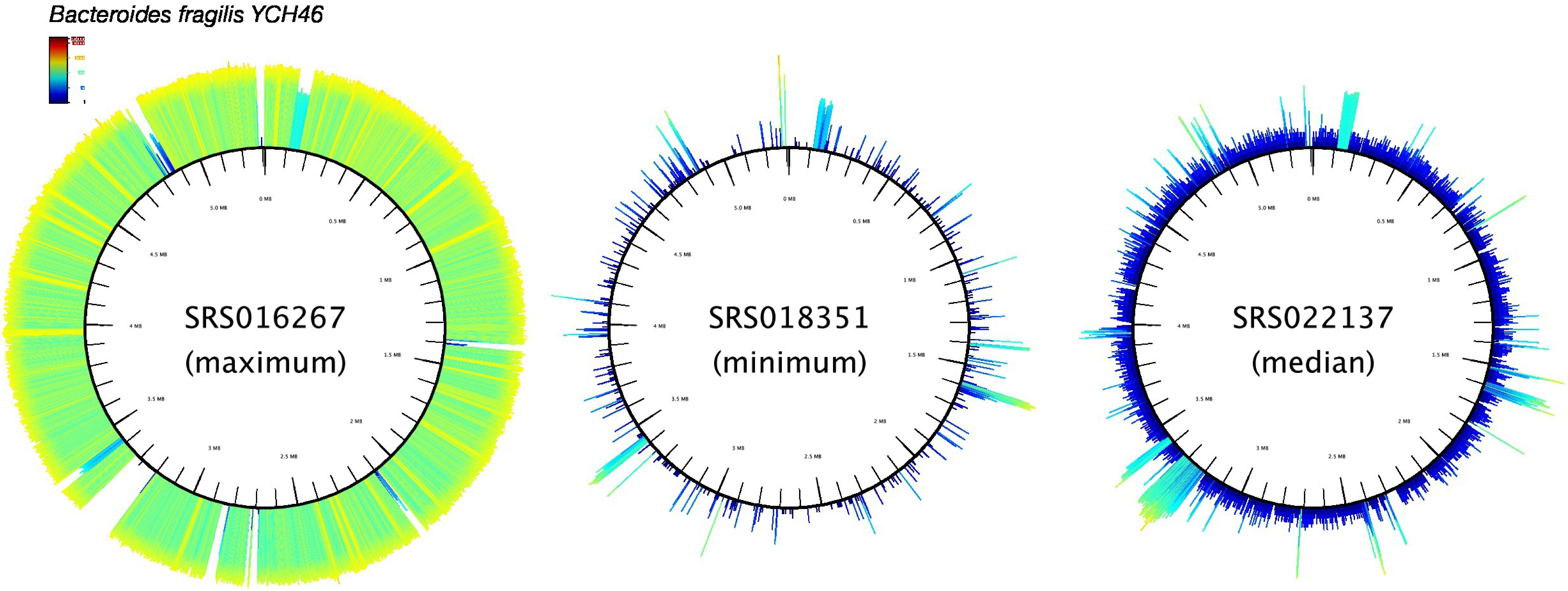

Bacteroides fragilis YCH46
SRS016267
(maximum)
SRS018351
(minimum)
SRS022137
(median)
0 MB
0.5 MB
1 MB
1.5 MB
2 MB
2.5 MB
3 MB
3.5 MB
4 MB
4.5 MB
5.0 MB

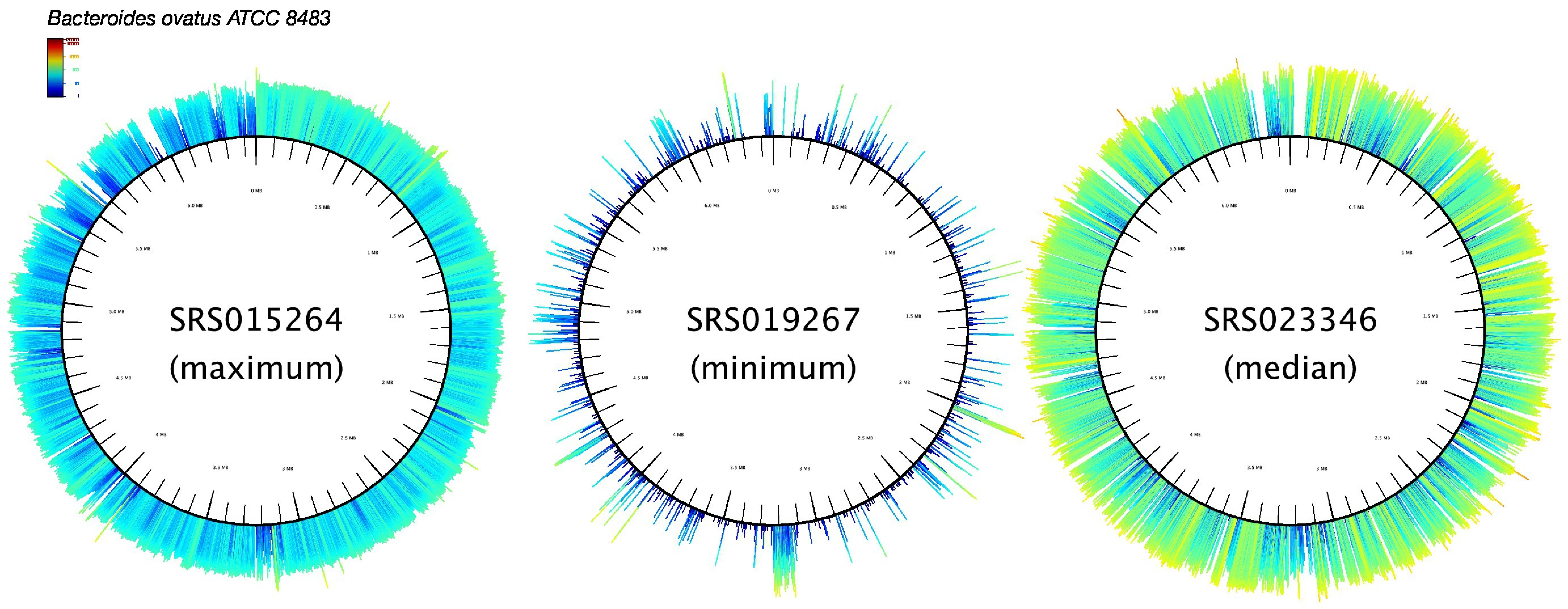

Bacteroides ovatus ATCC 8483
SRS015264
(maximum)
SRS019267
(minimum)
SRS023346
(median)

Bacteroides plebeius DSM 17135

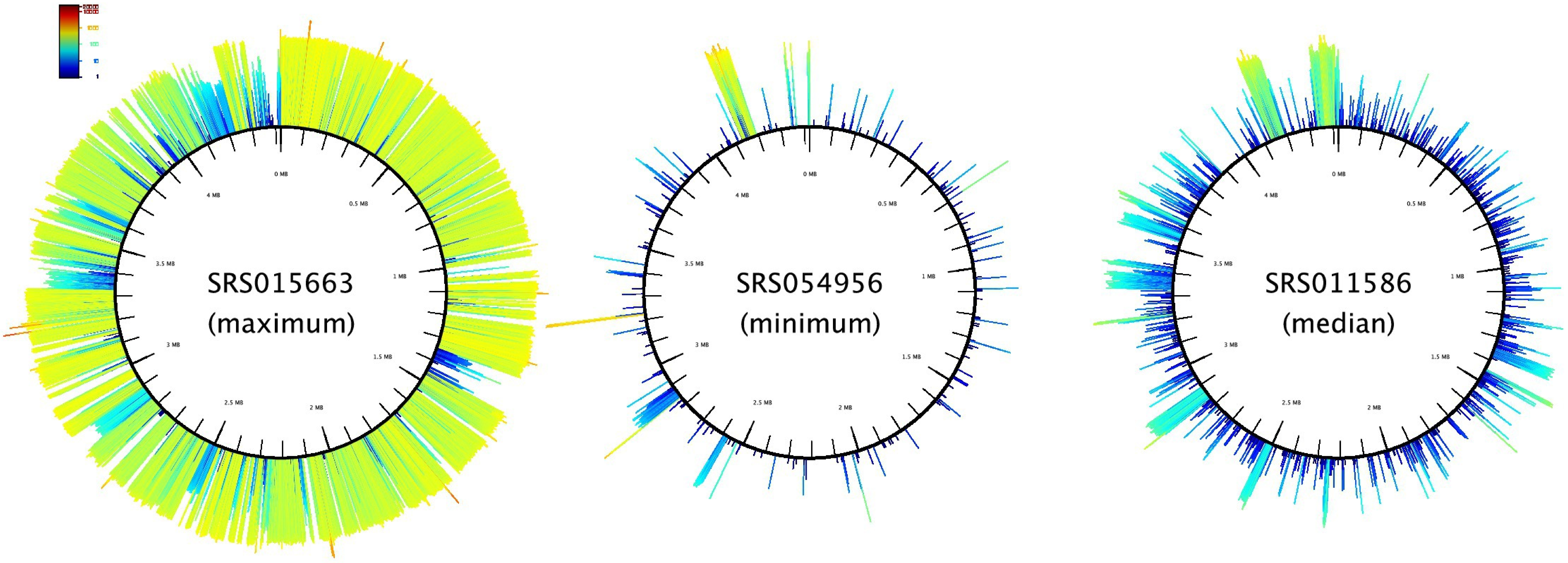

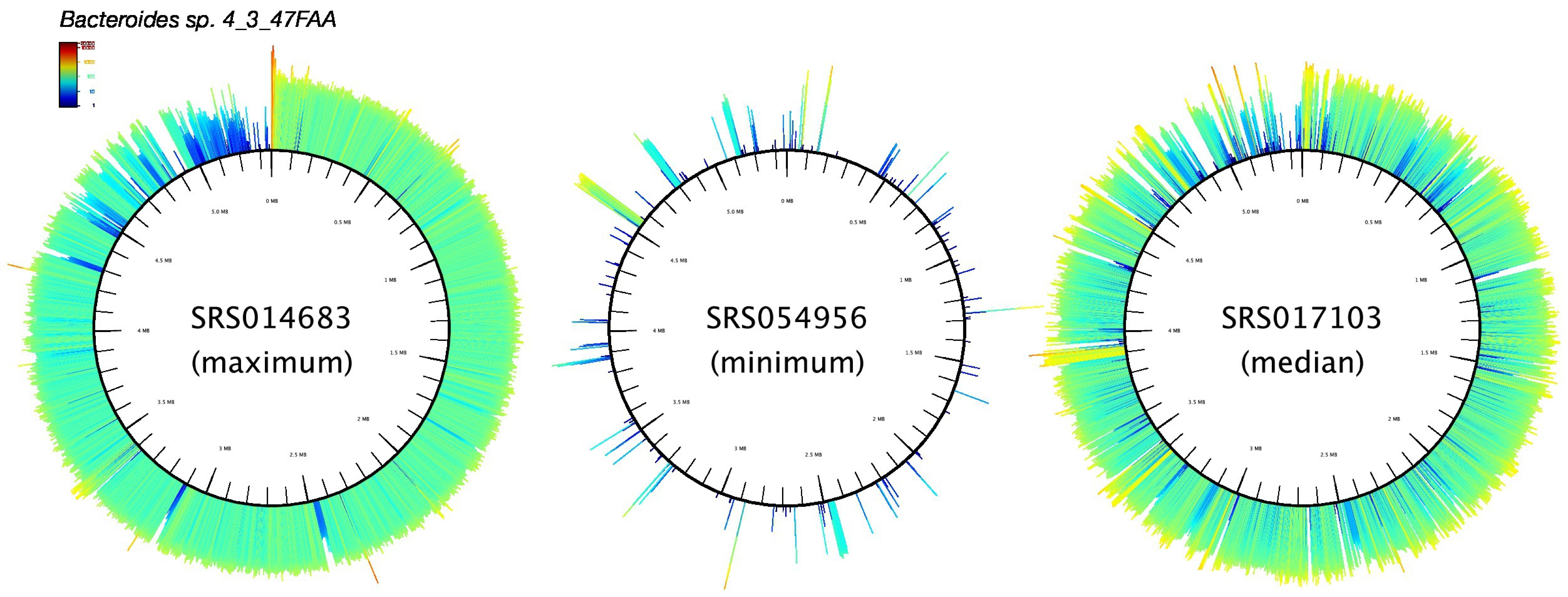

Bacteroides sp. 4_3_47FAA
SRS014683
(maximum)
SRS054956
(minimum)
SRS017103
(median)
0 MB
0.5 MB
1 MB
1.5 MB
2 MB
2.5 MB
3 MB
3.5 MB
4 MB
4.5 MB
5.0 MB

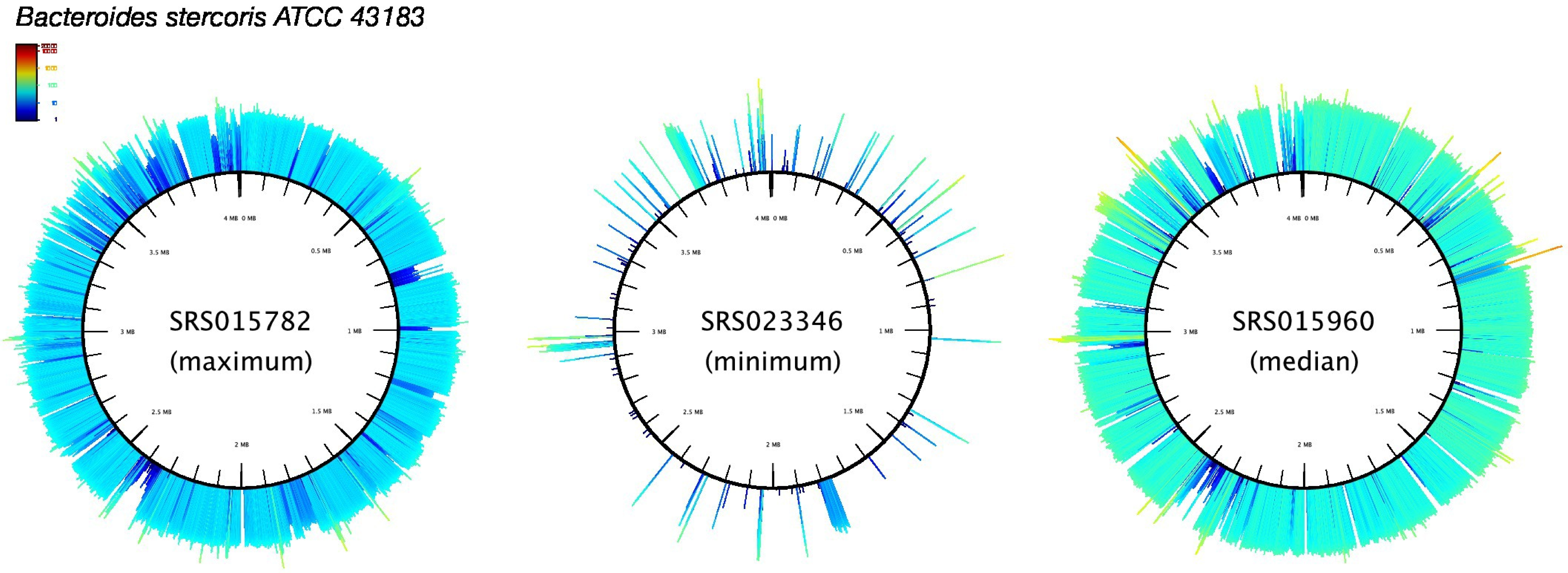
Bacteroides stercoris ATCC 43183
SRS015782
(maximum)
SRS023346
(minimum)
SRS015960
(median)
4 MB
0 MB
0.5 MB
1 MB
1.5 MB
2 MB
2.5 MB
3 MB
3.5 MB

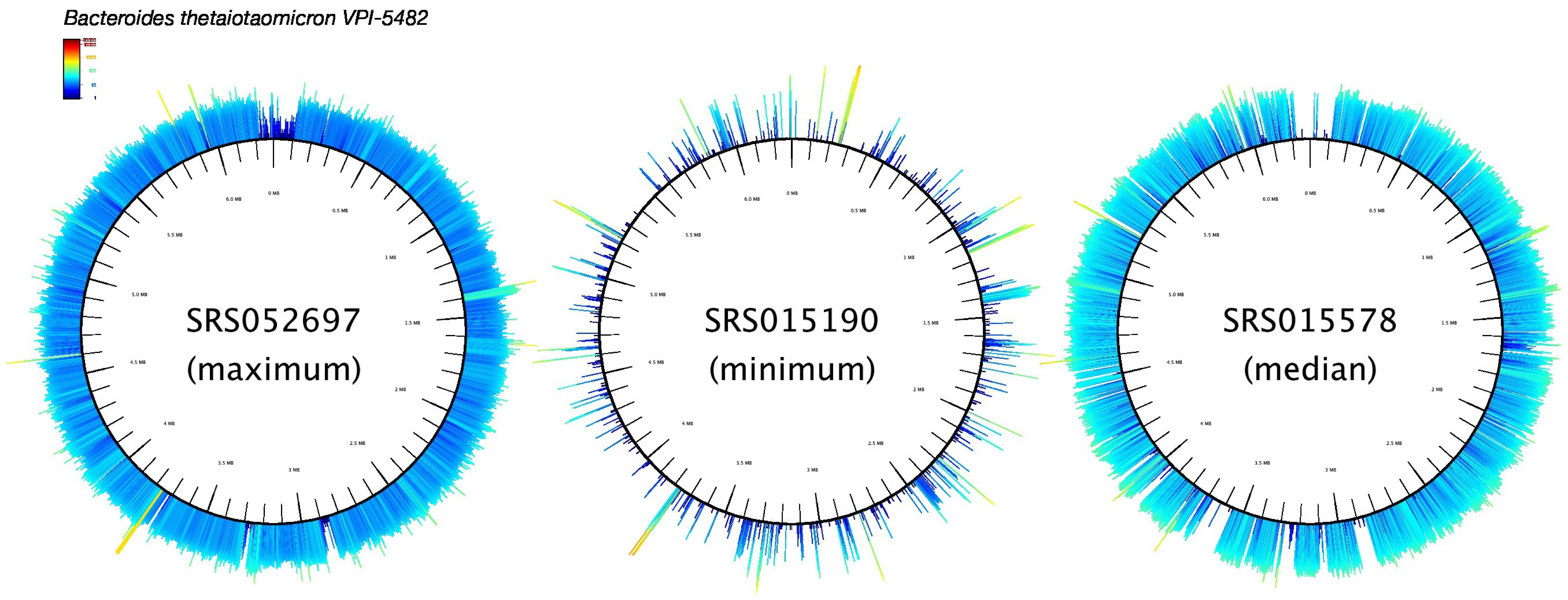
Bacteroides thetaiotaomicron VPI-5482
SRS052697
(maximum)
SRS015190
(minimum)
SRS015578
(median)
0 MB
0.5 MB
1 MB
1.5 MB
2 MB
2.5 MB
3 MB
3.5 MB
4 MB
4.5 MB
5.0 MB
5.5 MB
6.0 MB

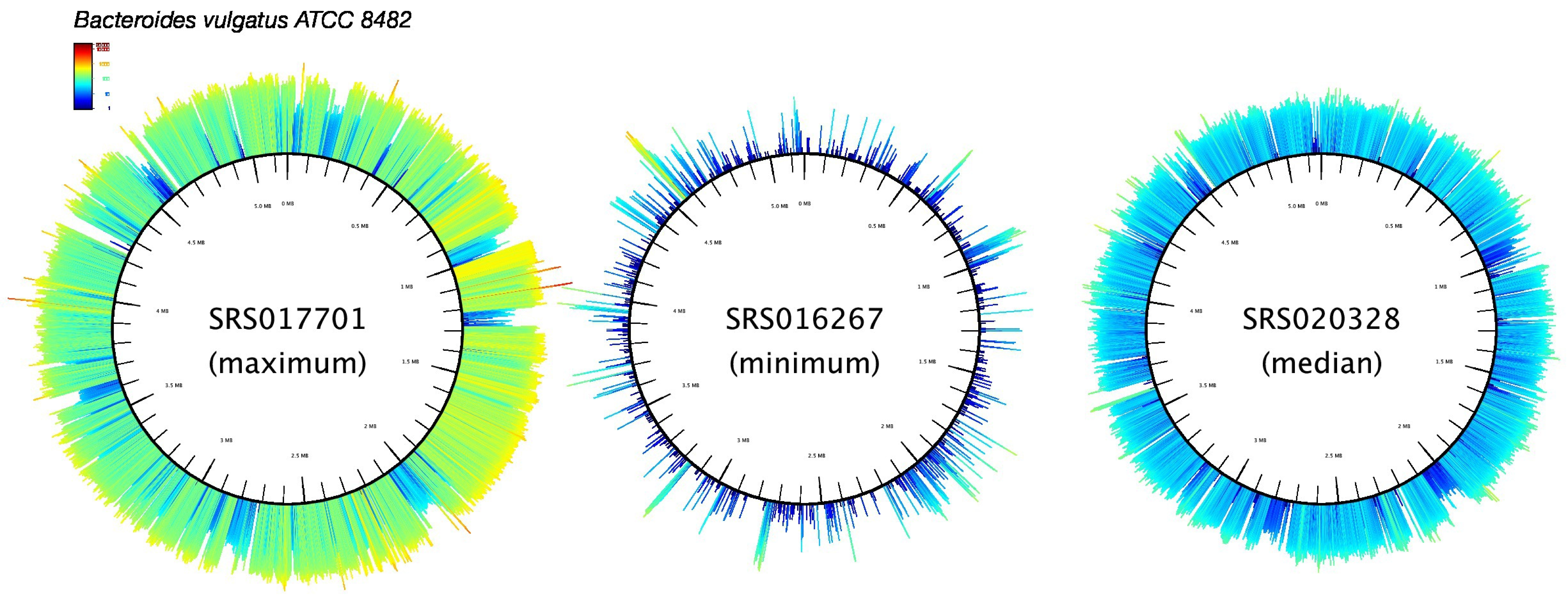
Bacteroides vulgatus ATCC 8482
SRS017701
(maximum)
SRS016267
(minimum)
SRS020328
(median)
0 MB
0.5 MB
1 MB
1.5 MB
2 MB
2.5 MB
3 MB
3.5 MB
4 MB
4.5 MB
5.0 MB

Bacteroides vulgatus PC510

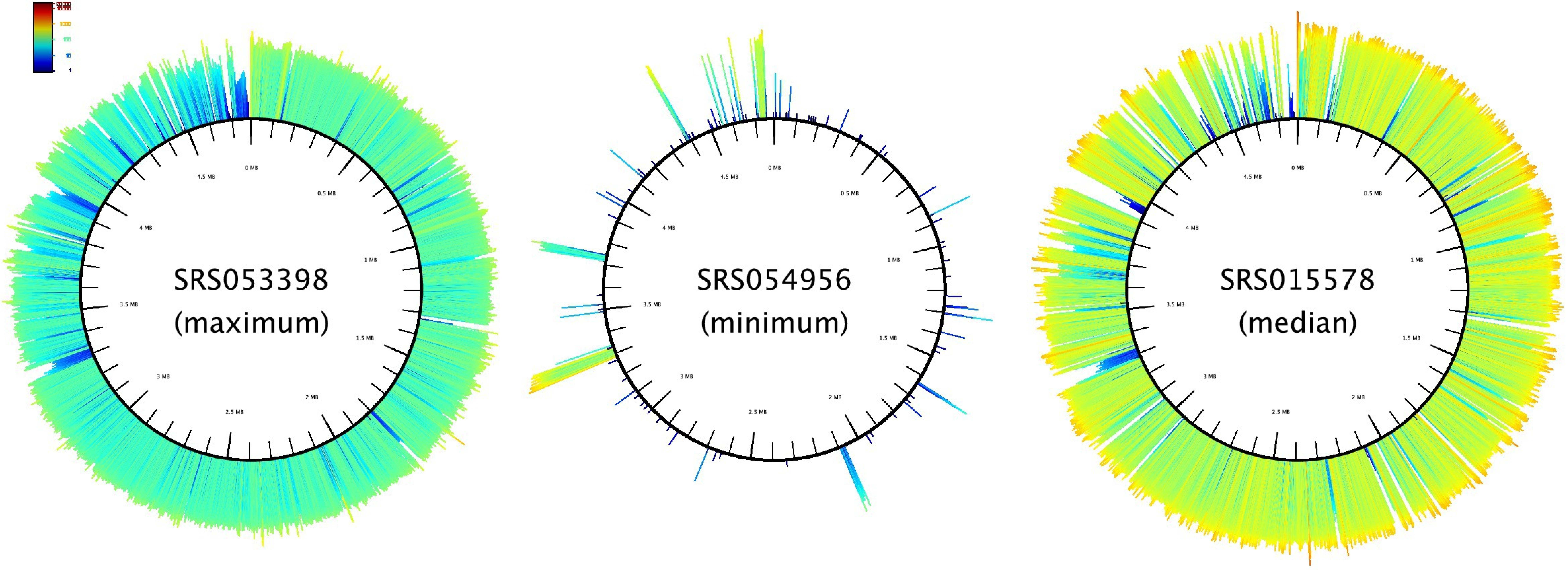

Burkholderiales bacterium 1_1_47

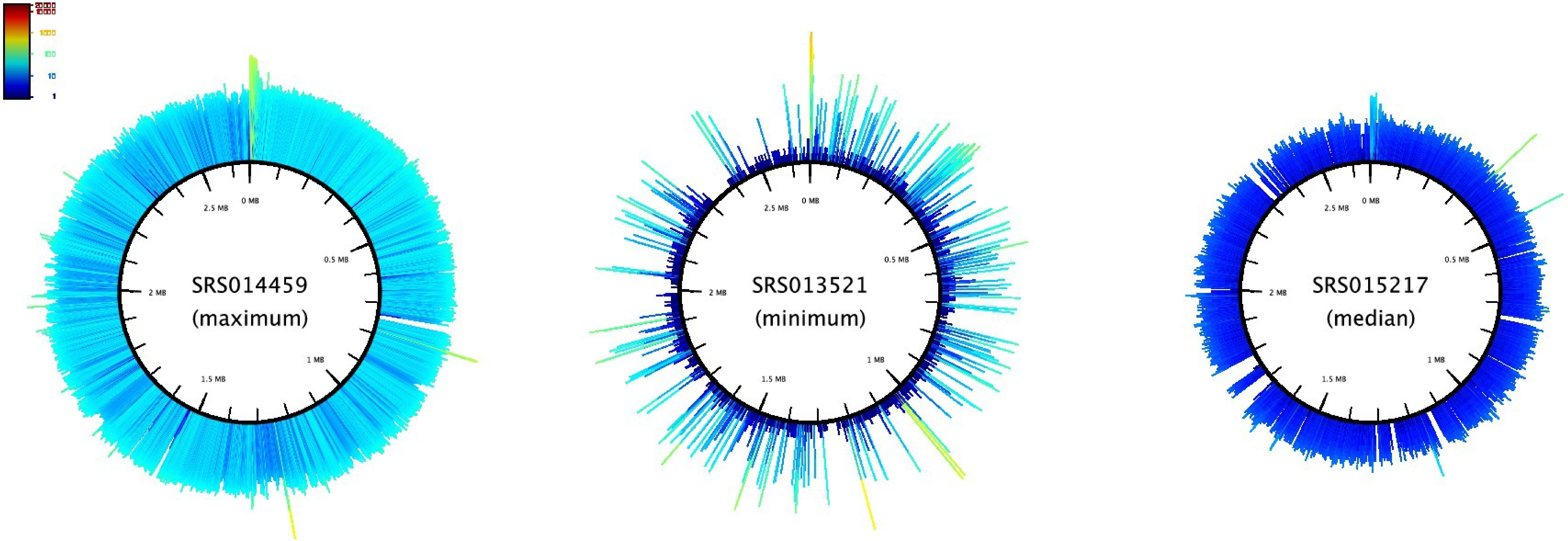

Butyrivibrio crossotus DSM 2876

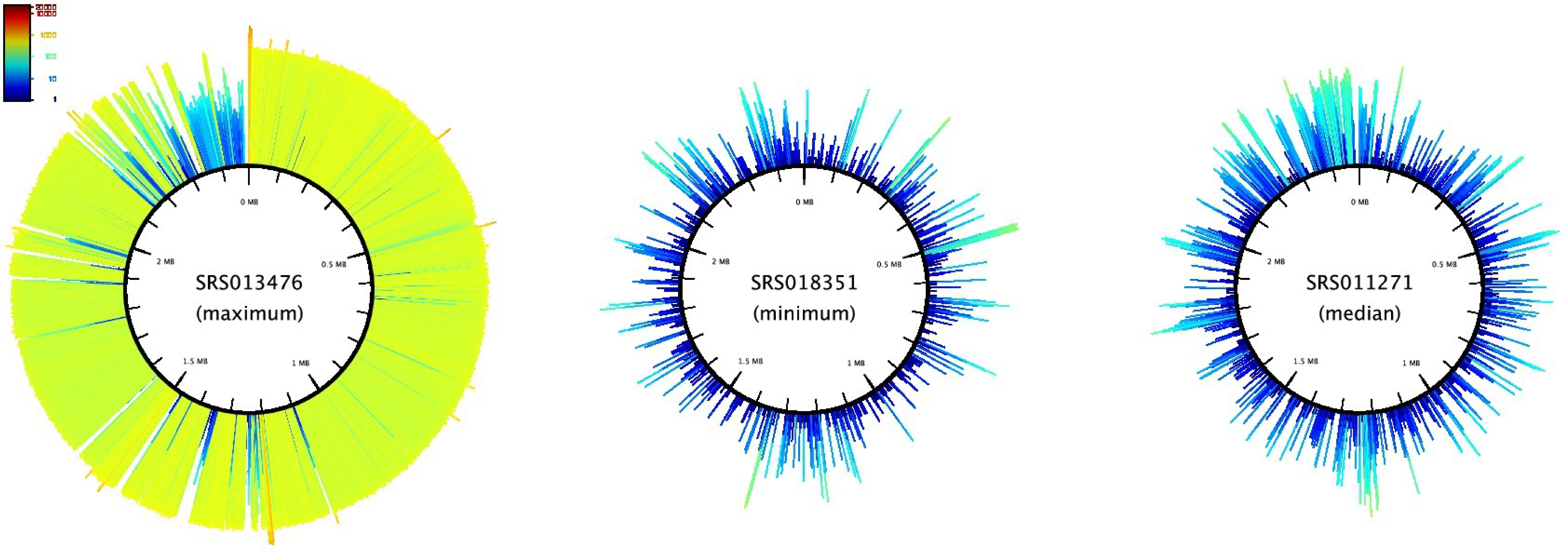

Collinsella aerofaciens ATCC 25986

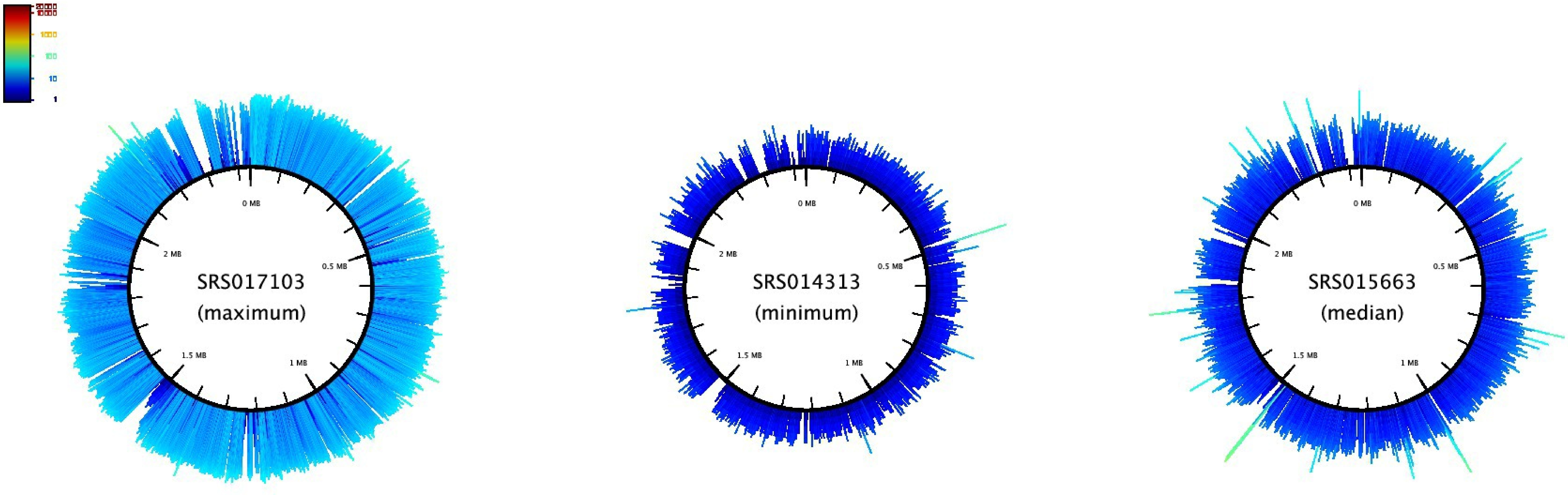

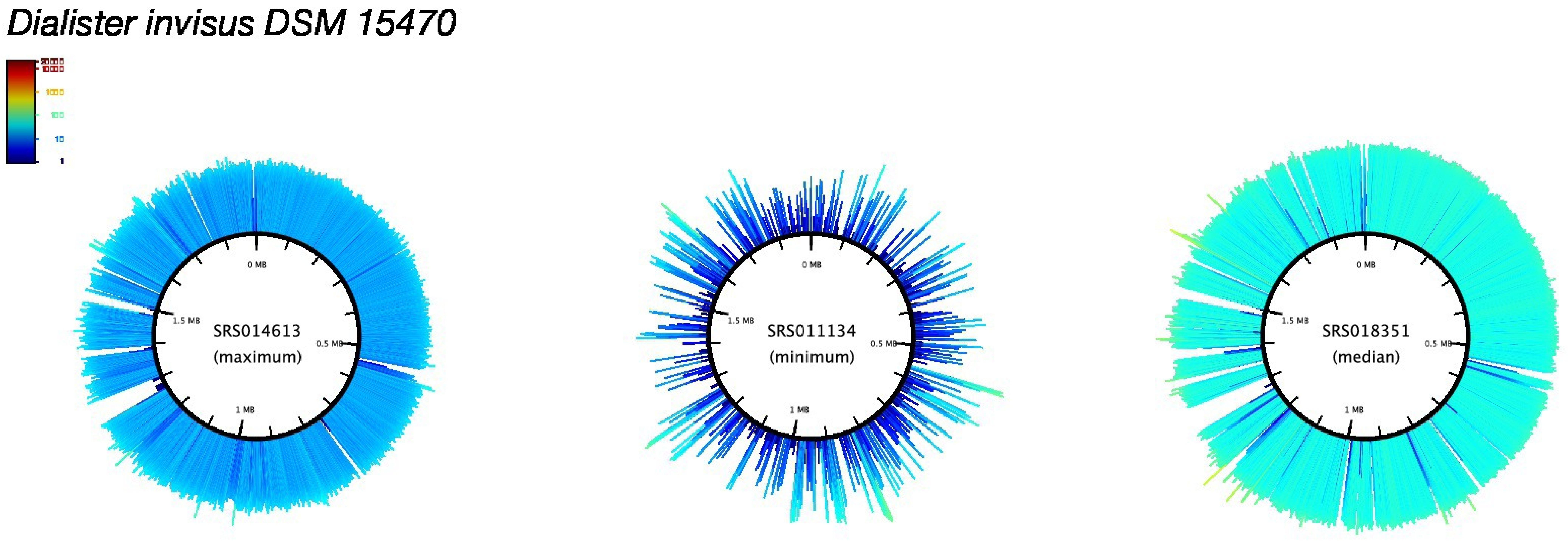
Dialister invisus DSM 15470
20000
10000
1000
100
10
1
0 MB
0.5 MB
1 MB
1.5 MB
SRS014613
(maximum)
SRS011134
(minimum)
SRS018351
(median)

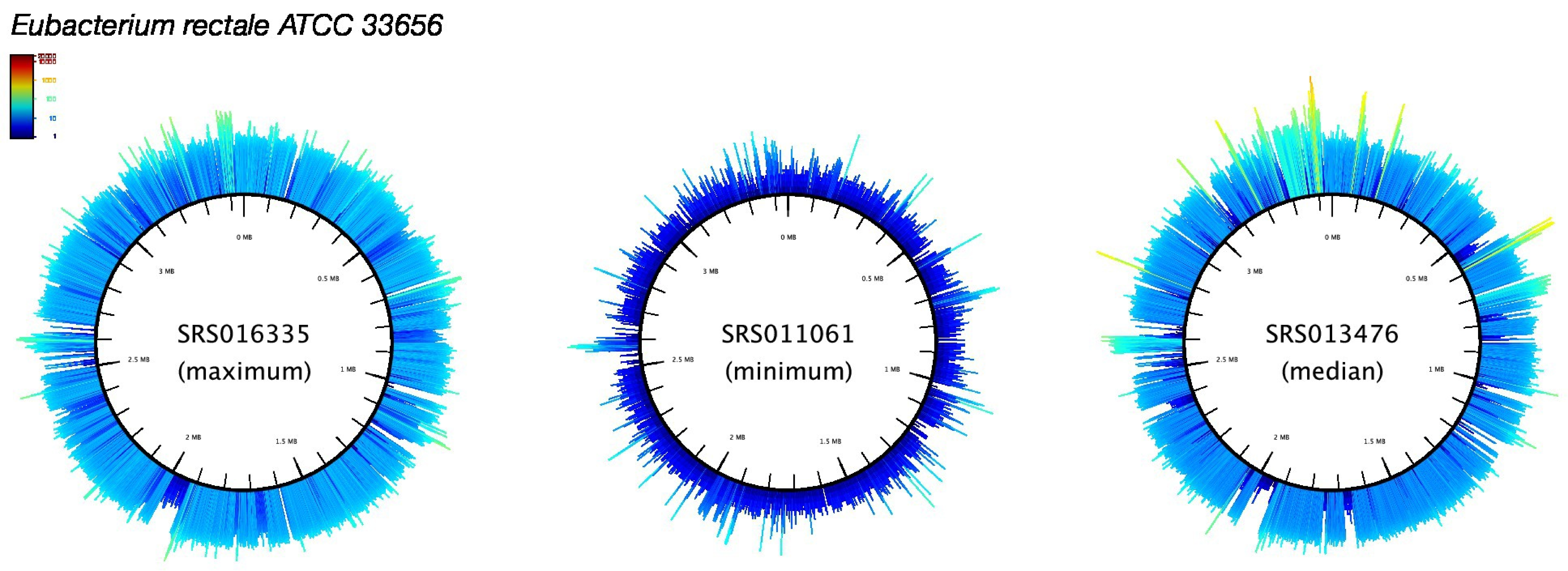
Eubacterium rectale ATCC 33656
1000
100
10
1
0 MB
0.5 MB
1 MB
1.5 MB
2 MB
2.5 MB
3 MB
SRS016335
(maximum)
SRS011061
(minimum)
SRS013476
(median)

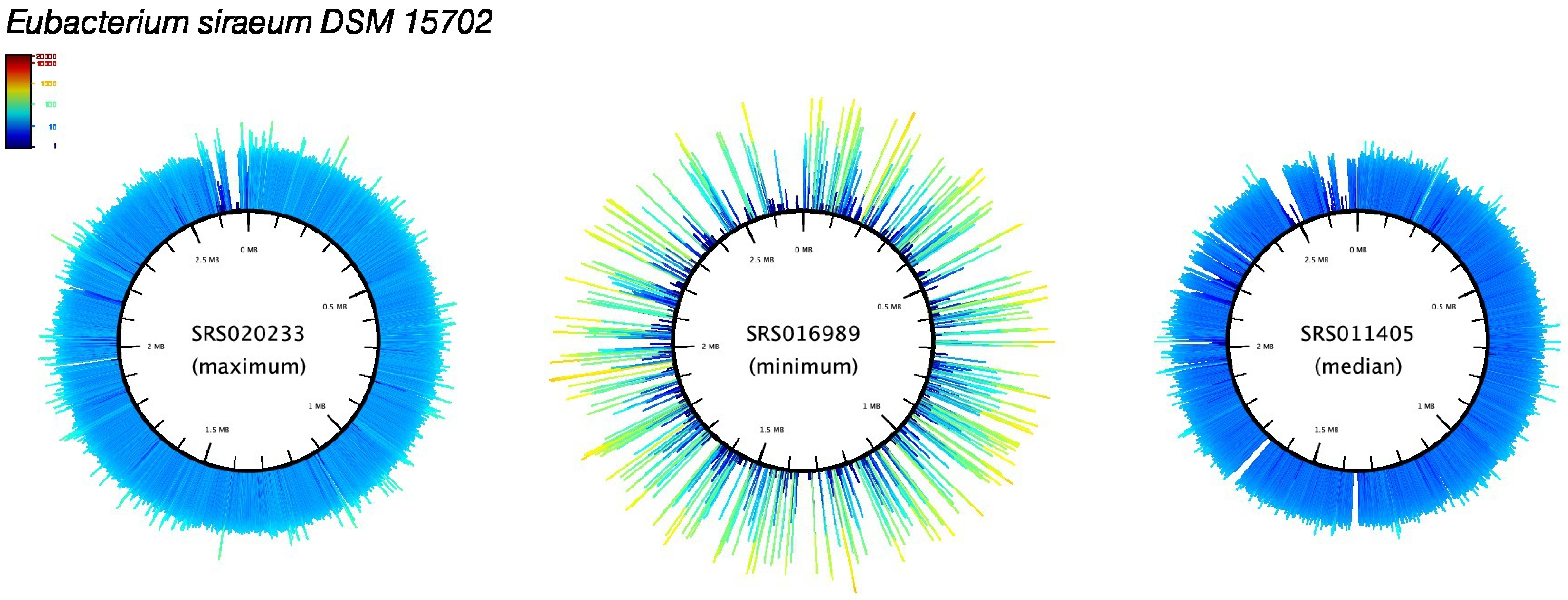
Eubacterium siraeum DSM 15702
20000
10000
1000
100
10
1
0 MB
0.5 MB
1 MB
1.5 MB
2 MB
2.5 MB
SRS020233
(maximum)
SRS016989
(minimum)
SRS011405
(median)

Faecalibacterium prausnitzii M21/2

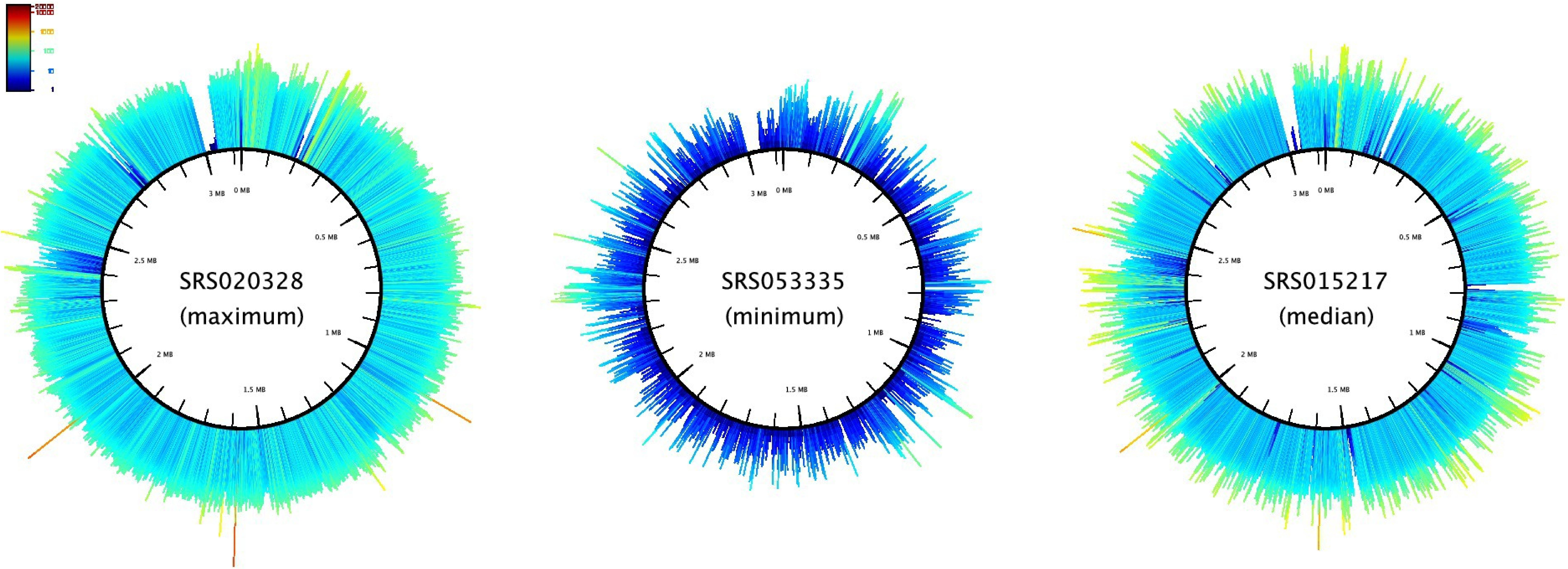

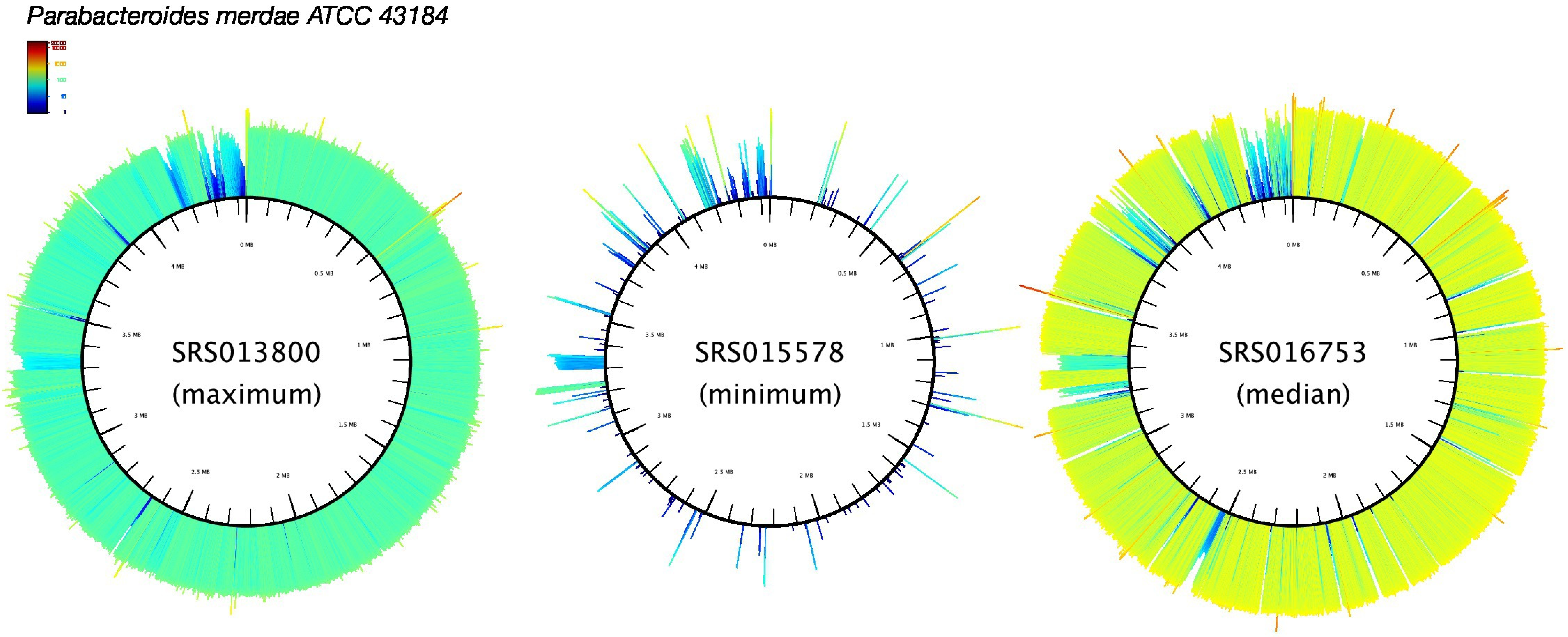
Parabacteroides merdae ATCC 43184
SRS013800
(maximum)
SRS015578
(minimum)
SRS016753
(median)
0 MB
0.5 MB
1 MB
1.5 MB
2 MB
2.5 MB
3 MB
3.5 MB
4 MB

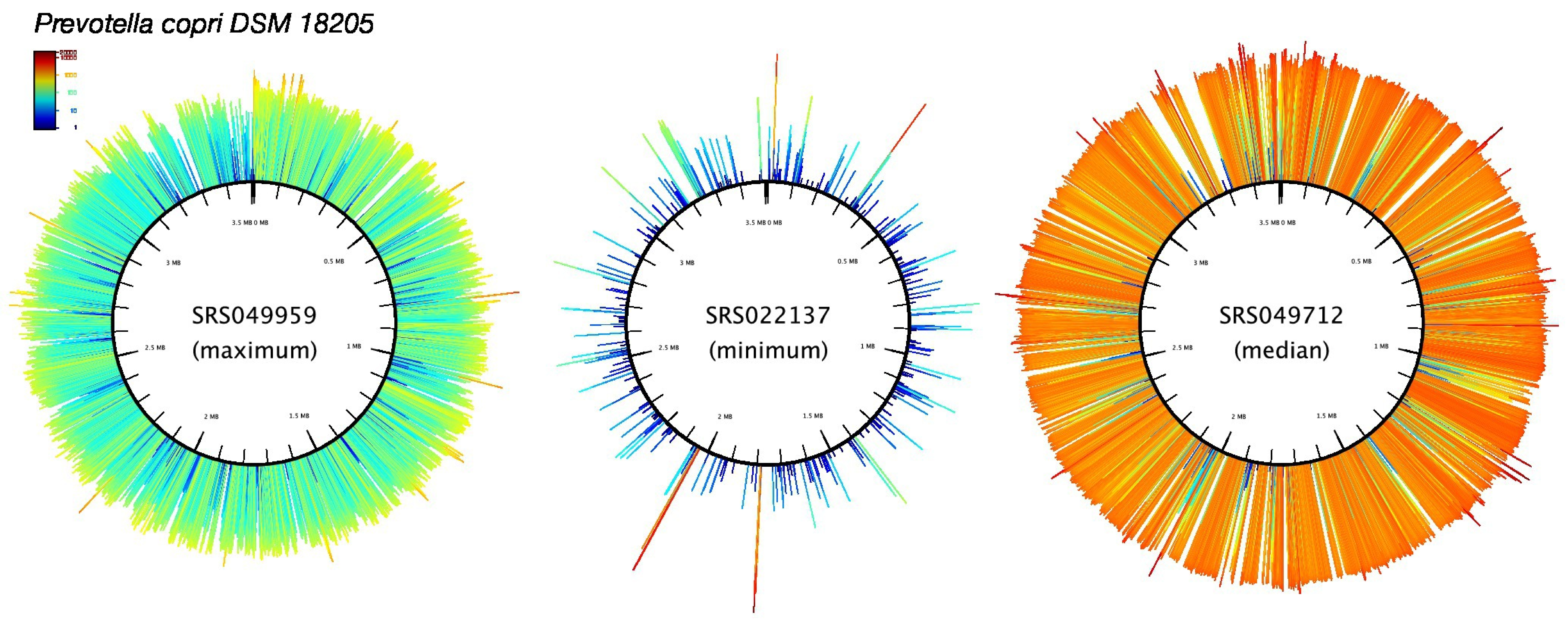

Prevotella copri DSM 18205
20000
10000
1000
100
10
1
3.5 MB 0 MB
0.5 MB
1 MB
1.5 MB
2 MB
2.5 MB
3 MB
SRS049959
(maximum)
SRS022137
(minimum)
SRS049712
(median)

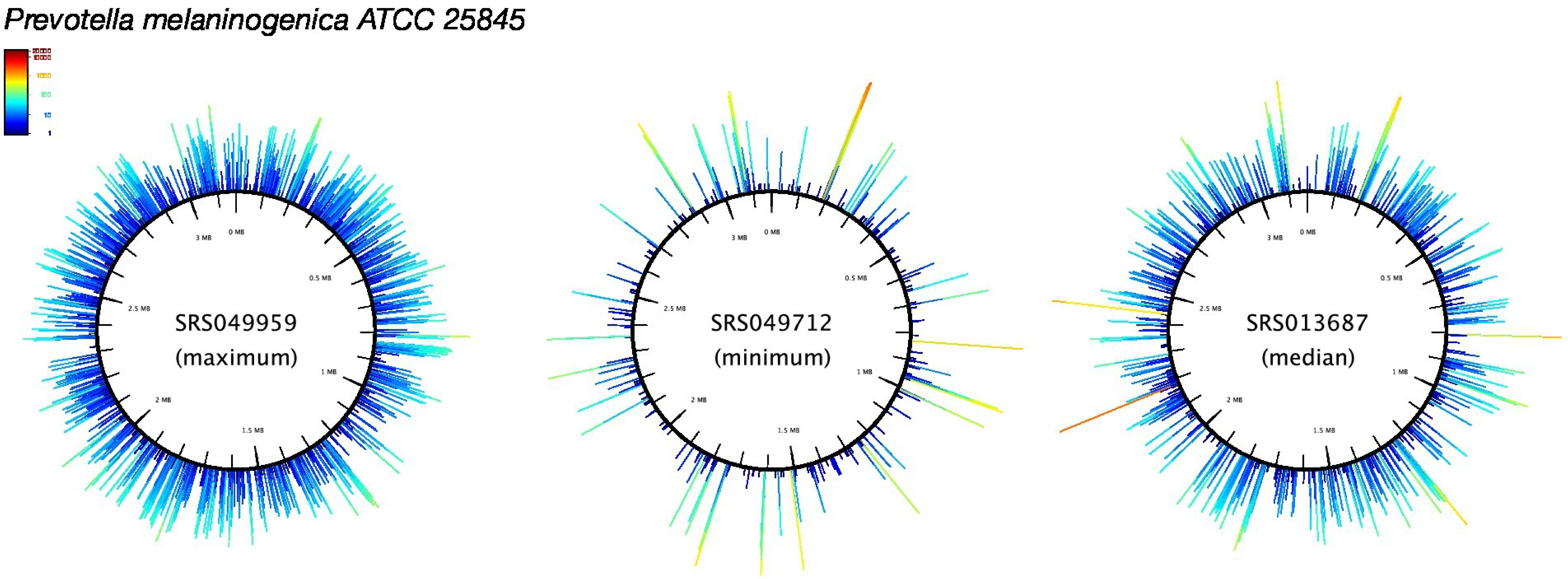

Prevotella melaninogenica ATCC 25845
20000
10000
1000
100
10
1
0 MB
0.5 MB
1 MB
1.5 MB
2 MB
2.5 MB
3 MB
SRS049959
(maximum)
SRS049712
(minimum)
SRS013687
(median)

Ruminococcus torques ATCC 27756

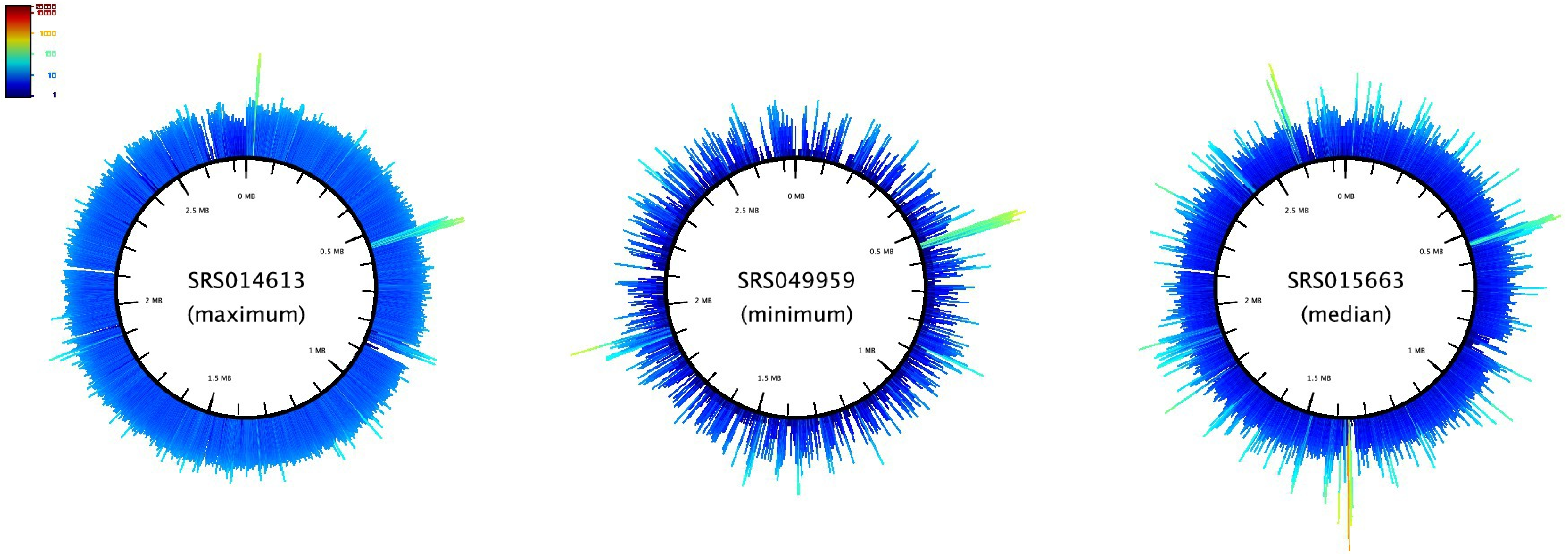

Veillonella dispar ATCC 17748

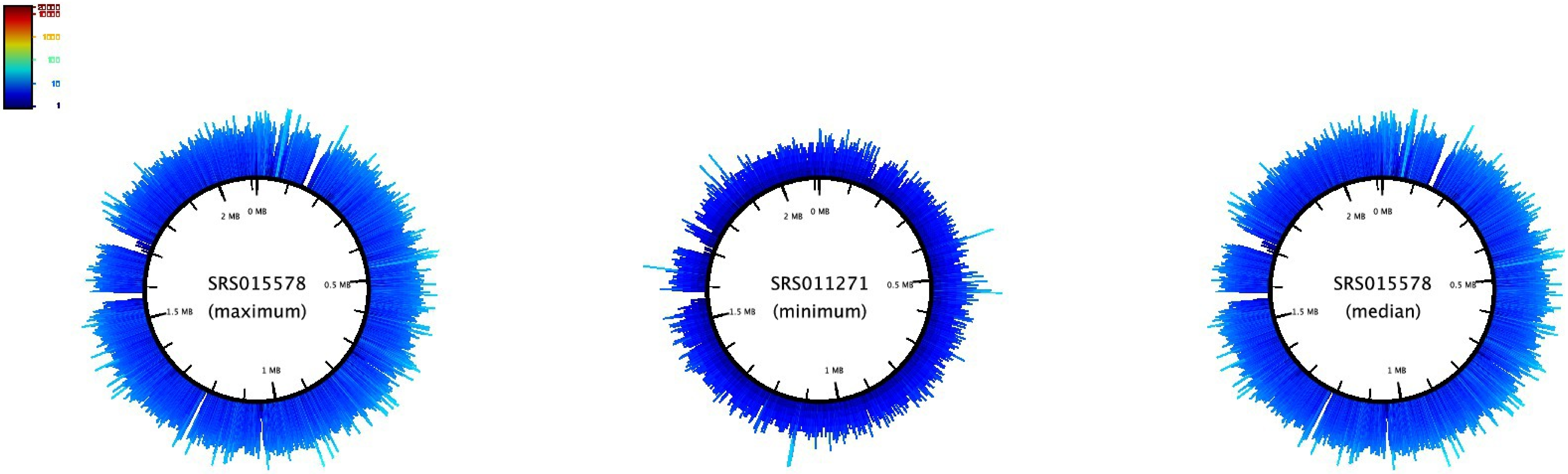

Streptococcus parasanguinis ATCC 15912

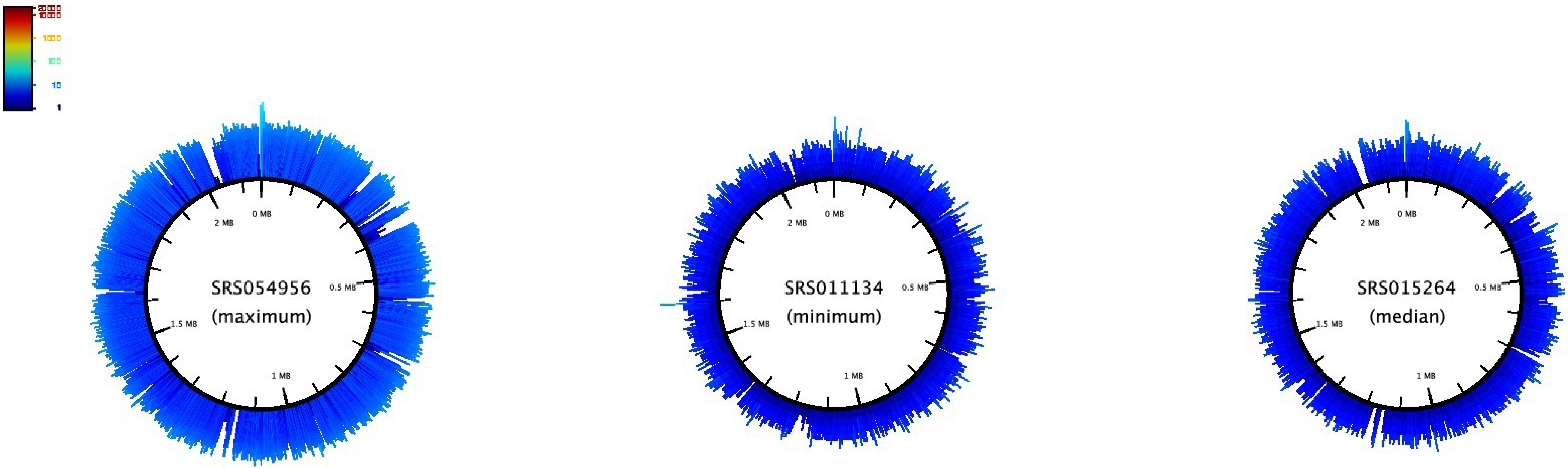

Streptococcus salivarius SK126

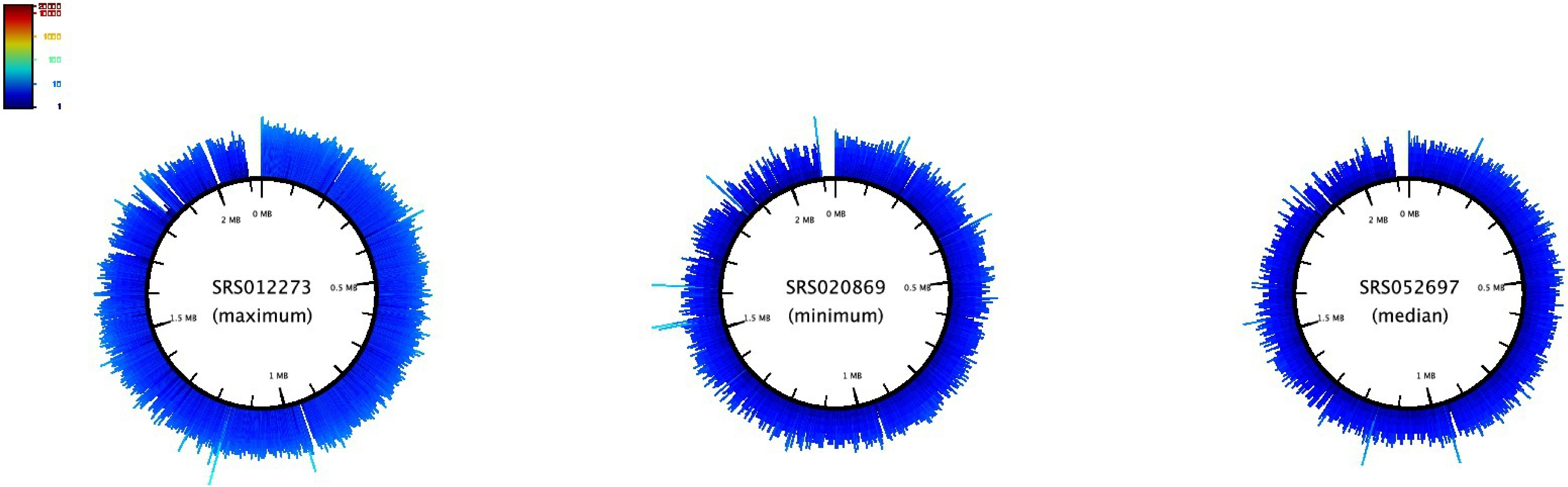

Supplement: Figure S1 — Genome coverage plots. (A) Genome coverage for the 30 strains mentioned in the text visualized in a boxplot. The box indicates the 25th–75th percentile, and the median coverage is indicated by a horizontal line in the box. The diamond represents the average and the outliers are visualized using dots. The whiskers of the box are the lowest and highest observation of coverage. (B) Read recruitment to the genomes of all the 30 strains mentioned in the text. For each strain, we show a coverage map for three subjects: the stool samples with the maximum, minimum and median coverage. Each peak represents the average coverage for a 100 bp bin both in color and length (on a log scale). All genomes are draft sequences, and consist of 3 contigs (Akkermansia muciniphila ATCC BAA-835, Bacteroides vulgatus ATCC 8482 and Eubacterium rectale ATCC 33656) to 1575 contigs (Bacteroides cellulosilyticus DSM 14838). Here, the contigs from draft genome sequences are ordered by their average coverage (from high to low) as determined from the sample with the maximum coverage. (PDF) [file pone.0097279.s001.pdf]
