# Supplementary material for: The Prevalence of Species and Strains in the Human Microbiome: A Resource for Experimental Efforts
Source: PLoS One. 2014 May 14;9(5):e97279. doi: 10.1371/journal.pone.0097279 (PMC4020798; doi:10.1371/journal.pone.0097279)
Supplement: Figure S2 — Heat map of clustered strain abundance in individual body sites. These figures show a heat map representation of the relative abundance of each strain (y-axis) for each sample (x-axis) for the 6 body sites individually as determined by depth X breadth of coverage (see Methods). The abundances are hierarchically clustered using Spearman rank correlation with average linkage. (PDF) [file pone.0097279.s002.pdf]

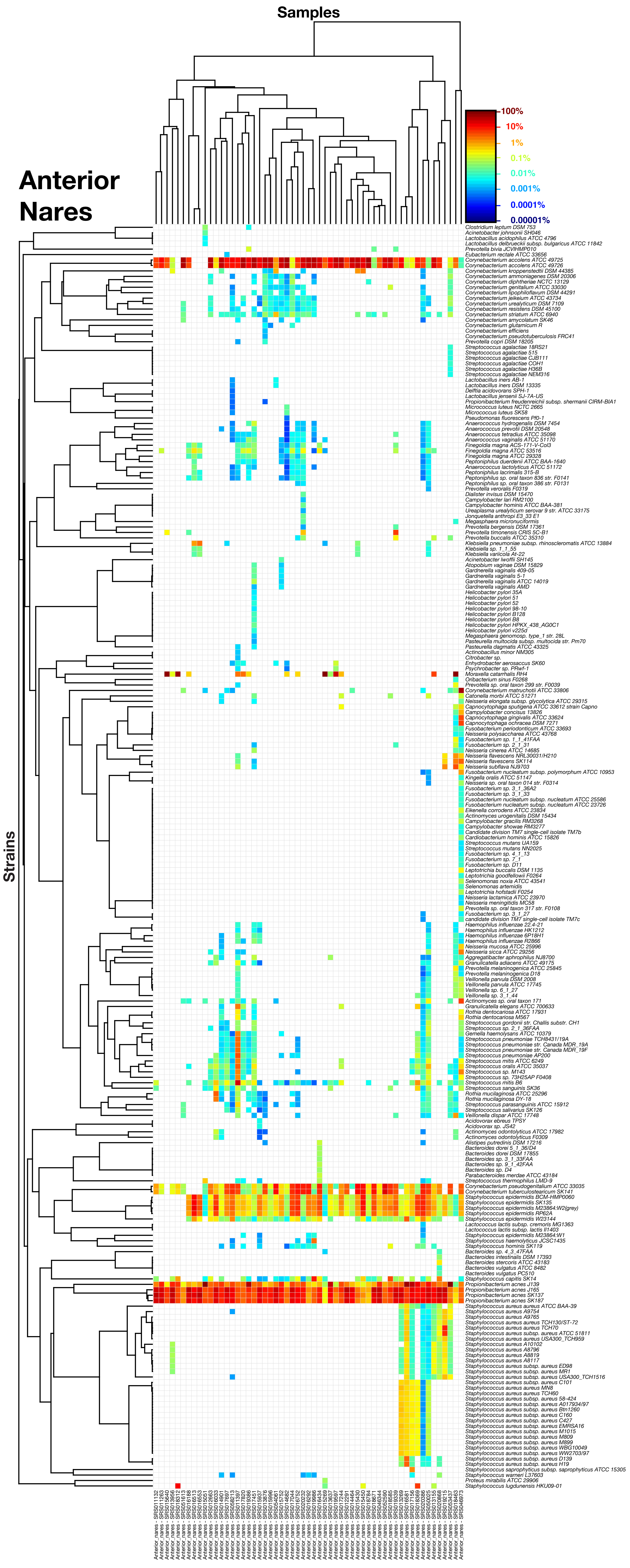

Samples
Anterior Nares
Strains
100%
10%
1%
0.1%
0.01%
0.001%
0.0001%
0.00001%
Clostridium leptum DSM 753
Acinetobacter johnsonii SH046
Lactobacillus acidophilus ATCC 4796
Lactobacillus delbrueckii subsp. bulgaricus ATCC 11842
Prevotella bivia JCVIHMP010
Eubacterium rectale ATCC 33656
Corynebacterium accolens ATCC 49725
Corynebacterium accolens ATCC 49726
Corynebacterium kroppenstedtii DSM 44385
Corynebacterium ammoniagenes DSM 20306
Corynebacterium diphtheriae NCTC 13129
Corynebacterium genitalium ATCC 33030
Corynebacterium lipophiloflavum DSM 44291
Corynebacterium jeikeium ATCC 43734
Corynebacterium urealyticum DSM 7109
Corynebacterium resistens DSM 45100
Corynebacterium striatum ATCC 6940
Corynebacterium amycolatum SK46
Corynebacterium glutamicum R
Corynebacterium efficiens
Corynebacterium pseudotuberculosis FRC41
Prevotella copri DSM 18205
Streptococcus agalactiae 18RS21
Streptococcus agalactiae 515
Streptococcus agalactiae CJB111
Streptococcus agalactiae COH1
Streptococcus agalactiae H36B
Streptococcus agalactiae NEM316
Lactobacillus iners AB-1
Lactobacillus iners DSM 13335
Delftia acidovorans SPH-1
Lactobacillus jensenii SJ-7A-US
Propionibacterium freudenreichii subsp. shermanii CIRM-BIA1
Micrococcus luteus NCTC 2665
Micrococcus luteus SK58
Pseudomonas fluorescens Pf0-1
Anaerococcus hydrogenalis DSM 7454
Anaerococcus prevotii DSM 20548
Anaerococcus tetradius ATCC 35098
Anaerococcus vaginalis ATCC 51170
Finegoldia magna ACS-171-V-Col3
Finegoldia magna ATCC 53516
Finegoldia magna ATCC 29328
Peptoniphilus duerdenii ATCC BAA-1640
Anaerococcus lactolyticus ATCC 51172
Peptoniphilus lacrimalis 315-B
Peptoniphilus sp. oral taxon 836 str. F0141
Peptoniphilus sp. oral taxon 386 str. F0131
Prevotella veroralis F0319
Dialister invisus DSM 15470
Campylobacter lari RM2100
Campylobacter hominis ATCC BAA-381
Ureaplasma urealyticum serovar 9 str. ATCC 33175
Jonquetella anthropi E3_33 E1
Megasphaera micronuciformis
Prevotella bergensis DSM 17361
Prevotella timonensis CRIS 5C-B1
Prevotella buccalis ATCC 35310
Klebsiella pneumoniae subsp. rhinoscleromatis ATCC 13884
Klebsiella sp. 1_1_55
Klebsiella variicola At-22
Acinetobacter lwoffii SH145
Atopobium vaginae DSM 15829
Gardnerella vaginalis 409-05
Gardnerella vaginalis 5-1
Gardnerella vaginalis ATCC 14019
Gardnerella vaginalis AMD
Helicobacter pylori 35A
Helicobacter pylori 51
Helicobacter pylori 52
Helicobacter pylori 98-10
Helicobacter pylori B128
Helicobacter pylori B8
Helicobacter pylori HPKX_438_AG0C1
Helicobacter pylori v225d
Megasphaera genomosp. type_1 str. 28L
Pasteurella multocida subsp. multocida str. Pm70
Pasteurella dagmatis ATCC 43325
Actinobacillus minor NM305
Citrobacter sp.
Enhydrobacter aerosaccus SK60
Psychrobacter sp. PRwf-1
Moraxella catarrhalis RH4
Oribacterium sinus F0268
Prevotella sp. oral taxon 299 str. F0039
Corynebacterium matruchotii ATCC 33806
Catonella morbi ATCC 51271
Neisseria elongata subsp. glycolytica ATCC 29315
Capnocytophaga sputigena ATCC 33612 strain Capno
Campylobacter concisus 13826
Capnocytophaga gingivalis ATCC 33624
Capnocytophaga ochracea DSM 7271
Fusobacterium periodonticum ATCC 33693
Neisseria polysaccharea ATCC 43768
Fusobacterium sp. 1_1_41FAA
Fusobacterium sp. 2_1_31
Neisseria cinerea ATCC 14685
Neisseria flavescens NRL30031/H210
Neisseria flavescens SK114
Neisseria subflava NJ9703
Fusobacterium nucleatum subsp. polymorphum ATCC 10953
Kingella oralis ATCC 51147
Neisseria sp. oral taxon 014 str. F0314
Fusobacterium sp. 3_1_36A2
Fusobacterium sp. 3_1_33
Fusobacterium nucleatum subsp. nucleatum ATCC 25586
Fusobacterium nucleatum subsp. nucleatum ATCC 23726
Eikenella corrodens ATCC 23834
Actinomyces urogenitalis DSM 15434
Campylobacter gracilis RM3268
Campylobacter showae RM3277
Candidate division TM7 single-cell isolate TM7b
Cardiobacterium hominis ATCC 15826
Streptococcus mutans UA159
Streptococcus mutans NN2025
Fusobacterium sp. 4_1_13
Fusobacterium sp. 7_1
Fusobacterium sp. D11
Leptotrichia buccalis DSM 1135
Leptotrichia goodfellowii F0264
Selenomonas noxia ATCC 43541
Selenomonas artemidis
Leptotrichia hofstadii F0254
Neisseria lactamica ATCC 23970
Neisseria meningitidis MC58
Prevotella sp. oral taxon 317 str. F0108
Fusobacterium sp. 3_1_27
candidate division TM7 single-cell isolate TM7c
Haemophilus influenzae 22.4-21
Haemophilus influenzae HK1212
Haemophilus influenzae 6P18H1
Haemophilus influenzae R2866
Neisseria mucosa ATCC 25996
Neisseria sicca ATCC 29256
Aggregatibacter aphrophilus NJ8700
Granulicatella adiacens ATCC 49175
Prevotella melaninogenica ATCC 25845
Prevotella melaninogenica D18
Veillonella parvula DSM 2008
Veillonella parvula ATCC 17745
Veillonella sp. 6_1_27
Veillonella sp. 3_1_44
Actinomyces sp. oral taxon 171
Granulicatella elegans ATCC 700633
Rothia dentocariosa ATCC 17931
Rothia dentocariosa M567
Streptococcus gordonii str. Challis substr. CH1
Streptococcus sp. 2_1_36FAA
Gemella haemolysans ATCC 10379
Streptococcus pneumoniae TCH8431/19A
Streptococcus pneumoniae str. Canada MDR_19A
Streptococcus pneumoniae str. Canada MDR_19F
Streptococcus pneumoniae AP200
Streptococcus mitis ATCC 6249
Streptococcus oralis ATCC 35037
Streptococcus sp. M143
Streptococcus sp. 73H25AP F0408
Streptococcus mitis B6
Streptococcus sanguinis SK36
Rothia mucilaginosa ATCC 25296
Rothia mucilaginosa DY-18
Streptococcus parasanguinis ATCC 15912
Streptococcus salivarius SK126
Veillonella dispar ATCC 17748
Acidovorax ebreus TPSY
Acidovorax sp. JS42
Actinomyces odontolyticus ATCC 17982
Actinomyces odontolyticus F0309
Alistipes putredinis DSM 17216
Bacteroides dorei 5_1_36/D4
Bacteroides dorei DSM 17855
Bacteroides sp. 3_1_33FAA
Bacteroides sp. 9_1_42FAA
Bacteroides sp. D4
Parabacteroides merdae ATCC 43184
Streptococcus thermophilus LMD-9
Corynebacterium pseudogenitalium ATCC 33035
Corynebacterium tuberculostearicum SK141
Staphylococcus epidermidis BCM-HMP0060
Staphylococcus epidermidis SK135
Staphylococcus epidermidis M23864:W2(grey)
Staphylococcus epidermidis RP62A
Staphylococcus epidermidis W23144
Lactococcus lactis subsp. cremoris MG1363
Lactococcus lactis subsp. lactis Il1403
Staphylococcus epidermidis M23864:W1
Staphylococcus haemolyticus JCSC1435
Staphylococcus hominis SK119
Bacteroides sp. 4_3_47FAA
Bacteroides intestinalis DSM 17393
Bacteroides stercoris ATCC 43183
Bacteroides vulgatus ATCC 8482
Bacteroides vulgatus PC510
Staphylococcus capitis SK14
Propionibacterium acnes J139
Propionibacterium acnes J165
Propionibacterium acnes SK137
Propionibacterium acnes SK187
Staphylococcus aureus aureus ATCC BAA-39
Staphylococcus aureus A9754
Staphylococcus aureus A9765
Staphylococcus aureus aureus TCH130/ST-72
Staphylococcus aureus aureus TCH70
Staphylococcus aureus subsp. aureus ATCC 51811
Staphylococcus aureus aureus USA300_TCH959
Staphylococcus aureus A10102
Staphylococcus aureus A8796
Staphylococcus aureus A8819
Staphylococcus aureus A8117
Staphylococcus aureus subsp. aureus ED98
Staphylococcus aureus subsp. aureus MR1
Staphylococcus aureus subsp. aureus USA300_TCH1516
Staphylococcus aureus subsp. aureus C101
Staphylococcus aureus aureus MN8
Staphylococcus aureus aureus TCH60
Staphylococcus aureus subsp. aureus 58-424
Staphylococcus aureus subsp. aureus A017934/97
Staphylococcus aureus subsp. aureus Btn1260
Staphylococcus aureus subsp. aureus C160
Staphylococcus aureus subsp. aureus C427
Staphylococcus aureus subsp. aureus EMRSA16
Staphylococcus aureus subsp. aureus M1015
Staphylococcus aureus subsp. aureus M809
Staphylococcus aureus subsp. aureus M899
Staphylococcus aureus subsp. aureus WBG10049
Staphylococcus aureus subsp. aureus WW2703/97
Staphylococcus aureus subsp. aureus D139
Staphylococcus aureus subsp. aureus H19
Staphylococcus saprophyticus subsp. saprophyticus ATCC 15305
Staphylococcus warneri L37603
Proteus mirabilis ATCC 29906
Staphylococcus lugdunensis HKU09-01

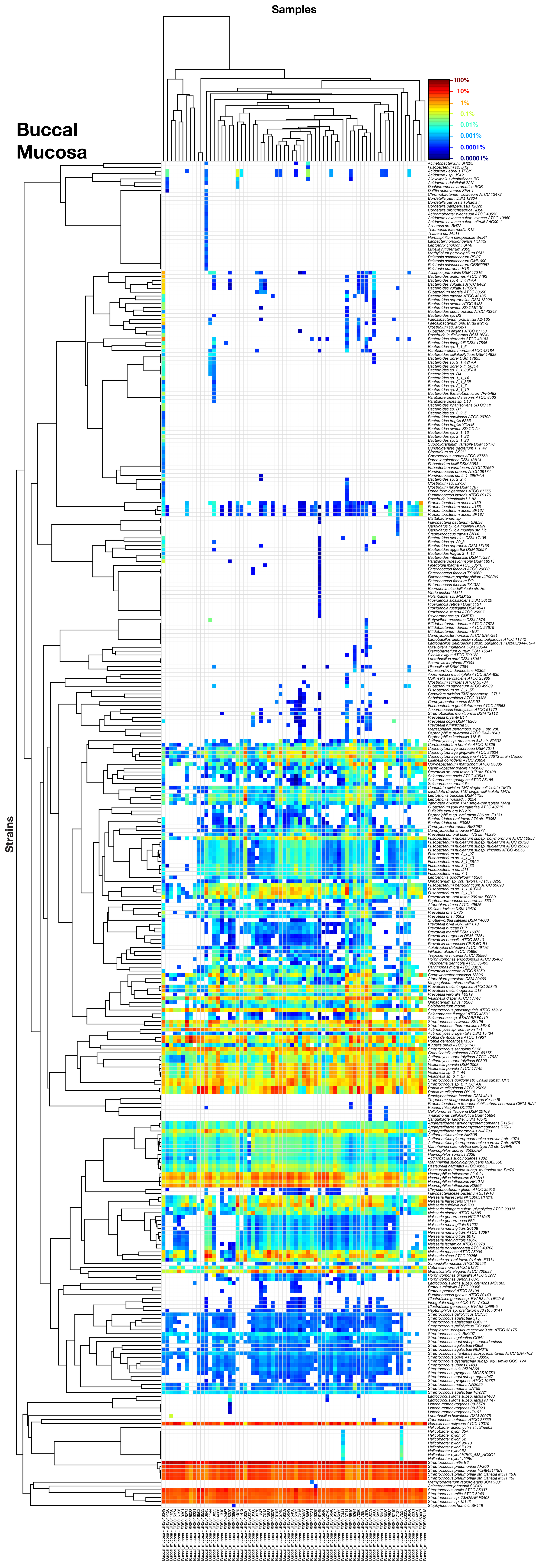
Samples
Buccal Mucosa
Strains
100%
10%
1%
0.1%
0.01%
0.001%
0.0001%
0.00001%

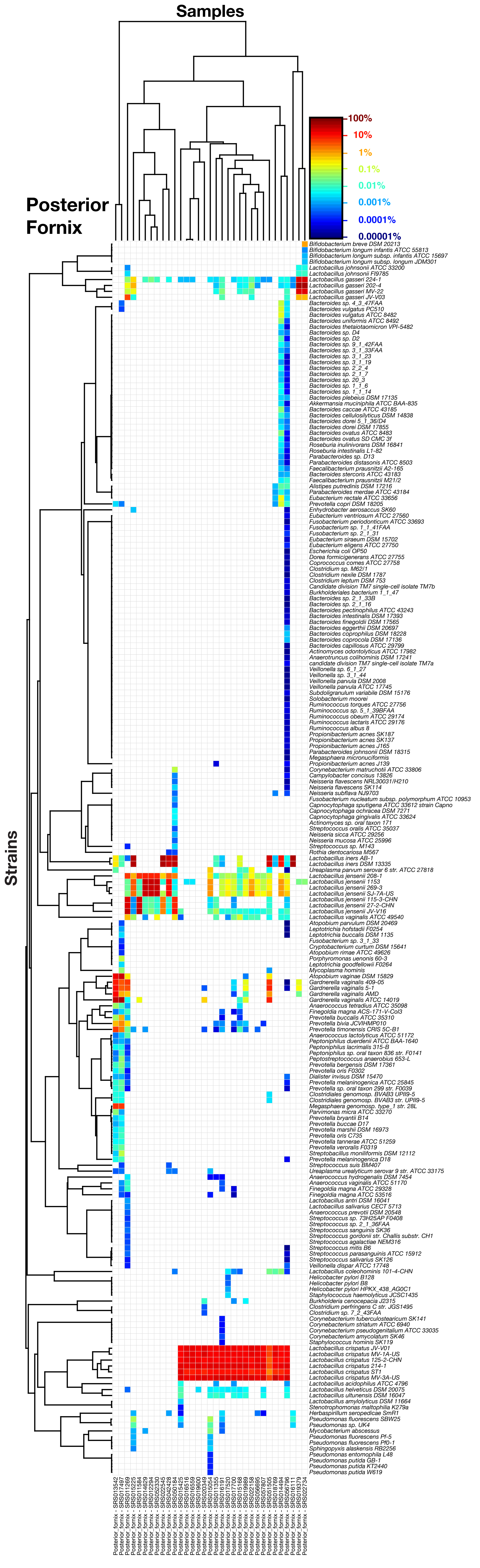

Samples
Posterior Fornix
100%
10%
1%
0.1%
0.01%
0.001%
0.0001%
0.00001%
Strains
Bifidobacterium breve DSM 20213
Bifidobacterium longum infantis ATCC 55813
Bifidobacterium longum subsp. infantis ATCC 15697
Bifidobacterium longum subsp. longum JDM301
Lactobacillus johnsonii ATCC 33200
Lactobacillus johnsonii FI9785
Lactobacillus gasseri 224-1
Lactobacillus gasseri 202-4
Lactobacillus gasseri MV-22
Lactobacillus gasseri JV-V03
Bacteroides sp. 4_3_47FAA
Bacteroides vulgatus PC510
Bacteroides vulgatus ATCC 8482
Bacteroides uniformis ATCC 8492
Bacteroides thetaiotaomicron VPI-5482
Bacteroides sp. D4
Bacteroides sp. D2
Bacteroides sp. 9_1_42FAA
Bacteroides sp. 3_1_33FAA
Bacteroides sp. 3_1_23
Bacteroides sp. 3_1_19
Bacteroides sp. 2_2_4
Bacteroides sp. 2_1_7
Bacteroides sp. 20_3
Bacteroides sp. 1_1_6
Bacteroides sp. 1_1_14
Bacteroides plebeius DSM 17135
Akkermansia muciniphila ATCC BAA-835
Bacteroides caccae ATCC 43185
Bacteroides cellulosilyticus DSM 14838
Bacteroides dorei 5_1_36/D4
Bacteroides dorei DSM 17855
Bacteroides ovatus ATCC 8483
Bacteroides ovatus SD CMC 3f
Roseburia inulinivorans DSM 16841
Roseburia intestinalis L1-82
Parabacteroides sp. D13
Parabacteroides distasonis ATCC 8503
Faecalibacterium prausnitzii A2-165
Bacteroides stercoris ATCC 43183
Faecalibacterium prausnitzii M21/2
Alistipes putredinis DSM 17216
Parabacteroides merdae ATCC 43184
Eubacterium rectale ATCC 33656
Prevotella copri DSM 18205
Enhydrobacter aerosaccus SK60
Eubacterium ventriosum ATCC 27560
Fusobacterium periodonticum ATCC 33693
Fusobacterium sp. 1_1_41FAA
Fusobacterium sp. 2_1_31
Eubacterium siraeum DSM 15702
Eubacterium eligens ATCC 27750
Escherichia coli OP50
Dorea formicigenerans ATCC 27755
Coprococcus comes ATCC 27758
Clostridium sp. M62/1
Clostridium nexile DSM 1787
Clostridium leptum DSM 753
Candidate division TM7 single-cell isolate TM7b
Burkholderiales bacterium 1_1_47
Bacteroides sp. 2_1_33B
Bacteroides sp. 2_1_16
Bacteroides pectinophilus ATCC 43243
Bacteroides intestinalis DSM 17393
Bacteroides finegoldii DSM 17565
Bacteroides eggerthii DSM 20697
Bacteroides coprophilus DSM 18228
Bacteroides coprocola DSM 17136
Bacteroides capillosus ATCC 29799
Actinomyces odontolyticus ATCC 17982
Anaerotruncus colihominis DSM 17241
candidate division TM7 single-cell isolate TM7a
Veillonella sp. 6_1_27
Veillonella sp. 3_1_44
Veillonella parvula DSM 2008
Veillonella parvula ATCC 17745
Subdoligranulum variabile DSM 15176
Solobacterium moorei
Ruminococcus torques ATCC 27756
Ruminococcus sp. 5_1_39BFAA
Ruminococcus obeum ATCC 29174
Ruminococcus lactaris ATCC 29176
Ruminococcus albus 8
Propionibacterium acnes SK187
Propionibacterium acnes SK137
Propionibacterium acnes J165
Parabacteroides johnsonii DSM 18315
Megasphaera micronuciformis
Propionibacterium acnes J139
Corynebacterium matruchotii ATCC 33806
Campylobacter concisus 13826
Neisseria flavescens NRL30031/H210
Neisseria flavescens SK114
Neisseria subflava NJ9703
Fusobacterium nucleatum subsp. polymorphum ATCC 10953
Capnocytophaga sputigena ATCC 33612 strain Capno
Capnocytophaga ochracea DSM 7271
Capnocytophaga gingivalis ATCC 33624
Actinomyces sp. oral taxon 171
Streptococcus oralis ATCC 35037
Neisseria sicca ATCC 29256
Neisseria mucosa ATCC 25996
Streptococcus sp. M143
Rothia dentocariosa M567
Lactobacillus iners AB-1
Lactobacillus iners DSM 13335
Ureaplasma parvum serovar 6 str. ATCC 27818
Lactobacillus jensenii 208-1
Lactobacillus jensenii 1153
Lactobacillus jensenii 269-3
Lactobacillus jensenii SJ-7A-US
Lactobacillus jensenii 115-3-CHN
Lactobacillus jensenii 27-2-CHN
Lactobacillus jensenii JV-V16
Lactobacillus vaginalis ATCC 49540
Atopobium parvulum DSM 20469
Leptotrichia hofstadii F0254
Leptotrichia buccalis DSM 1135
Fusobacterium sp. 3_1_33
Cryptobacterium curtum DSM 15641
Atopobium rimae ATCC 49626
Porphyromonas uenonis 60-3
Leptotrichia goodfellowii F0264
Mycoplasma hominis
Atopobium vaginae DSM 15829
Gardnerella vaginalis 409-05
Gardnerella vaginalis 5-1
Gardnerella vaginalis AMD
Gardnerella vaginalis ATCC 14019
Anaerococcus tetradius ATCC 35098
Finegoldia magna ACS-171-V-Col3
Prevotella buccalis ATCC 35310
Prevotella bivia JCVIHMP010
Prevotella timonensis CRIS 5C-B1
Anaerococcus lactolyticus ATCC 51172
Peptoniphilus duerdenii ATCC BAA-1640
Peptoniphilus lacrimalis 315-B
Peptoniphilus sp. oral taxon 836 str. F0141
Peptostreptococcus anaerobius 653-L
Prevotella bergensis DSM 17361
Prevotella oris F0302
Dialister invisus DSM 15470
Prevotella melaninogenica ATCC 25845
Prevotella sp. oral taxon 299 str. F0039
Clostridiales genomosp. BVAB3 UPII9-5
Clostridiales genomosp. BVAB3 str. UPII9-5
Megasphaera genomosp. type_1 str. 28L
Parvimonas micra ATCC 33270
Prevotella bryantii B14
Prevotella buccae D17
Prevotella marshii DSM 16973
Prevotella oris C735
Prevotella tannerae ATCC 51259
Prevotella veroralis F0319
Streptobacillus moniliformis DSM 12112
Prevotella melaninogenica D18
Streptococcus suis BM407
Ureaplasma urealyticum serovar 9 str. ATCC 33175
Anaerococcus hydrogenalis DSM 7454
Anaerococcus vaginalis ATCC 51170
Finegoldia magna ATCC 29328
Finegoldia magna ATCC 53516
Lactobacillus antri DSM 16041
Lactobacillus salivarius CECT 5713
Anaerococcus prevotii DSM 20548
Streptococcus sp. 73H25AP F0408
Streptococcus sp. 2_1_36FAA
Streptococcus sanguinis SK36
Streptococcus gordonii str. Challis substr. CH1
Streptococcus agalactiae NEM316
Streptococcus mitis B6
Streptococcus parasanguinis ATCC 15912
Streptococcus salivarius SK126
Veillonella dispar ATCC 17748
Lactobacillus coleohominis 101-4-CHN
Helicobacter pylori B128
Helicobacter pylori B8
Helicobacter pylori HPKX_438_AG0C1
Staphylococcus haemolyticus JCSC1435
Burkholderia cenocepacia J2315
Clostridium perfringens C str. JGS1495
Clostridium sp. 7_2_43FAA
Corynebacterium tuberculostearicum SK141
Corynebacterium striatum ATCC 6940
Corynebacterium pseudogenitalium ATCC 33035
Corynebacterium amycolatum SK46
Staphylococcus hominis SK119
Lactobacillus crispatus JV-V01
Lactobacillus crispatus MV-1A-US
Lactobacillus crispatus 125-2-CHN
Lactobacillus crispatus 214-1
Lactobacillus crispatus ST1
Lactobacillus crispatus MV-3A-US
Lactobacillus acidophilus ATCC 4796
Lactobacillus helveticus DSM 20075
Lactobacillus ultunensis DSM 16047
Lactobacillus amylolyticus ATCC 11664
Stenotrophomonas maltophilia K279a
Herbaspirillum seropedicae SmR1
Pseudomonas fluorescens SBW25
Pseudomonas sp. UK4
Mycobacterium abscessus
Pseudomonas fluorescens Pf-5
Pseudomonas fluorescens Pf0-1
Sphingopyxis alaskensis RB2256
Pseudomonas entomophila L48
Pseudomonas putida GB-1
Pseudomonas putida KT2440
Pseudomonas putida W619
Posterior_fornix - SRS013542
Posterior_fornix - SRS017497
Posterior_fornix - SRS011269
Posterior_fornix - SRS015225
Posterior_fornix - SRS011584
Posterior_fornix - SRS014629
Posterior_fornix - SRS012294
Posterior_fornix - SRS052330
Posterior_fornix - SRS022545
Posterior_fornix - SRS042428
Posterior_fornix - SRS050184
Posterior_fornix - SRS015425
Posterior_fornix - SRS016516
Posterior_fornix - SRS016559
Posterior_fornix - SRS019600
Posterior_fornix - SRS020349
Posterior_fornix - SRS015054
Posterior_fornix - SRS011355
Posterior_fornix - SRS016191
Posterior_fornix - SRS017520
Posterior_fornix - SRS017700
Posterior_fornix - SRS015168
Posterior_fornix - SRS019989
Posterior_fornix - SRS022158
Posterior_fornix - SRS056695
Posterior_fornix - SRS057807
Posterior_fornix - SRS051505
Posterior_fornix - SRS018769
Posterior_fornix - SRS014494
Posterior_fornix - SRS056796
Posterior_fornix - SRS016111
Posterior_fornix - SRS019379
Posterior_fornix - SRS022734

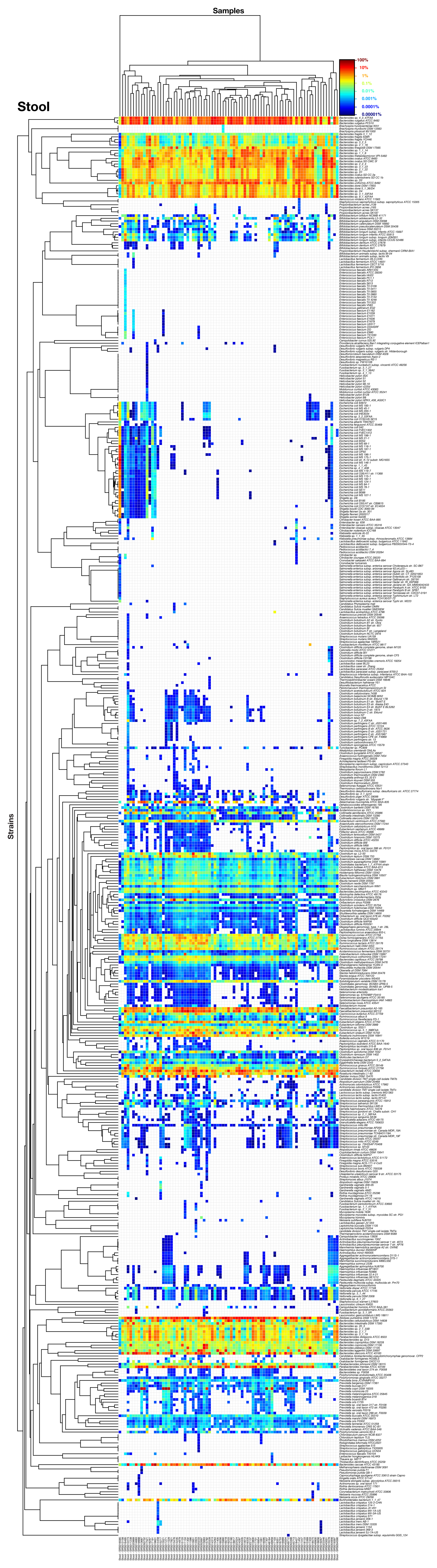
Samples
Stool
Strains
100%
10%
1%
0.1%
0.01%
0.001%
0.0001%
0.00001%

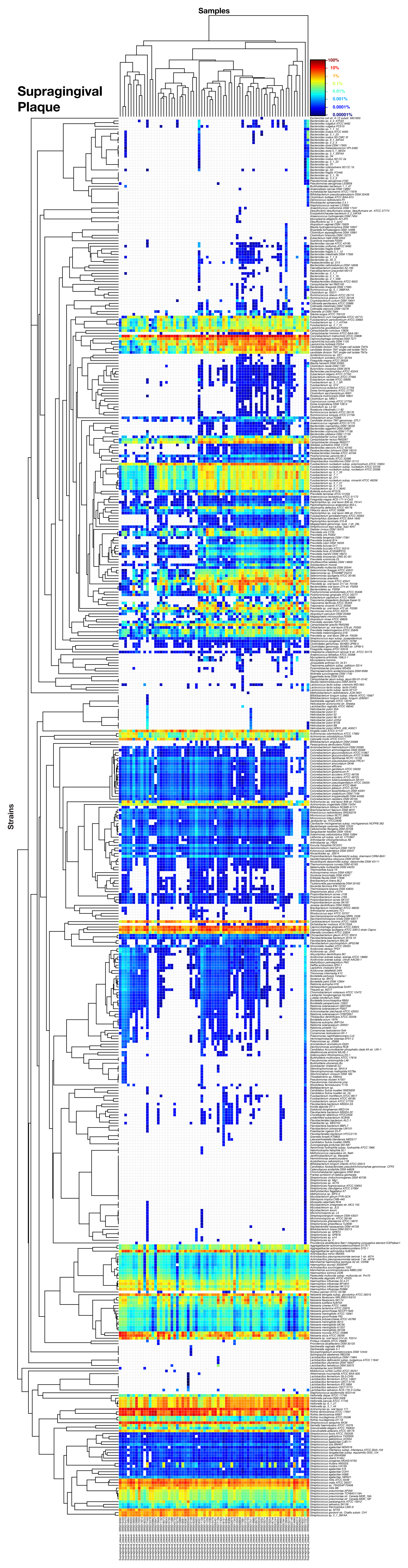
Samples
Supragingival Plaque
Strains
100%
10%
1%
0.1%
0.01%
0.001%
0.0001%
0.00001%

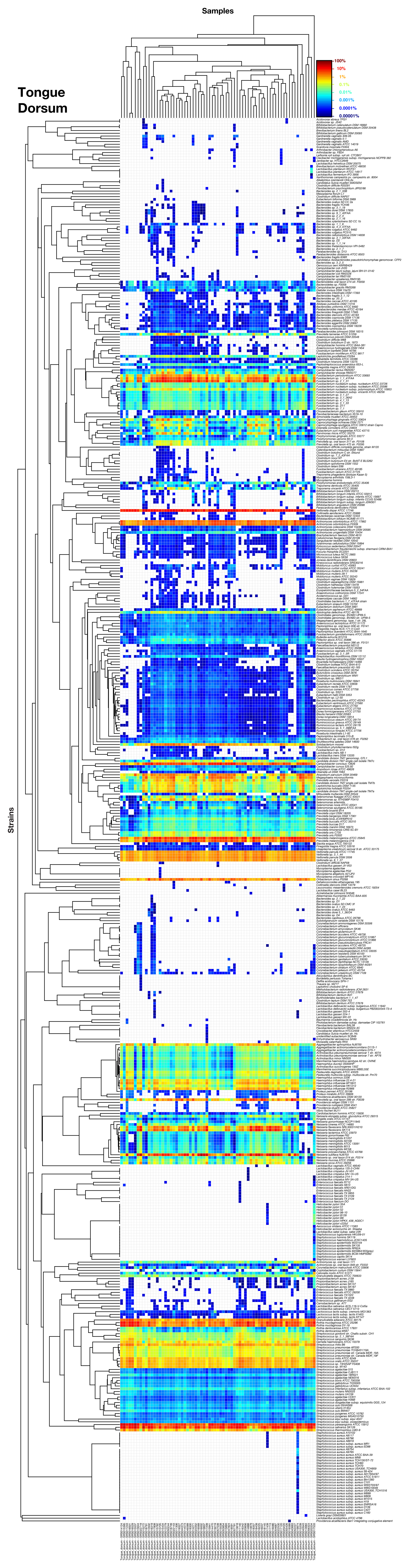
Samples
Tongue Dorsum
Strains
100%
10%
1%
0.1%
0.01%
0.001%
0.0001%
0.00001%
