# Supplementary figures and images for: The Prevalence of Species and Strains in the Human Microbiome: A Resource for Experimental Efforts
Source: PLoS One. 2014 May 14;9(5):e97279. doi: 10.1371/journal.pone.0097279 (PMC4020798; doi:10.1371/journal.pone.0097279)

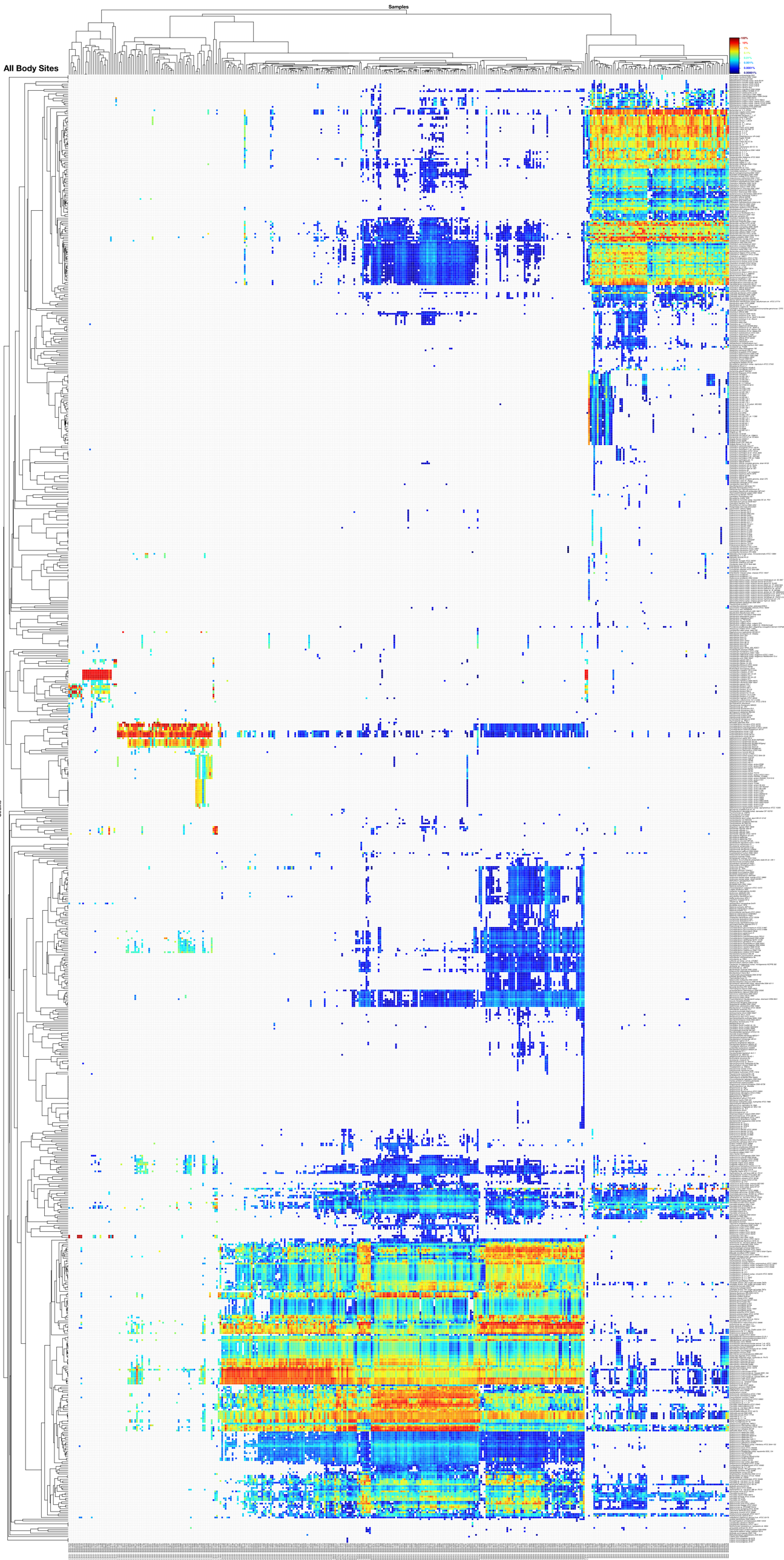

Supplement: Figure S3 — Heat maps of ordered strain abundance for all body sites combined. A heat map representation of the relative abundance of each strain (y-axis) for each sample (x-axis) for the 6 body sites combined as determined by depth X breadth of coverage (see Methods). The abundances are hierarchically clustered using Spearman rank correlation with average linkage. (PDF) [file pone.0097279.s003.pdf]

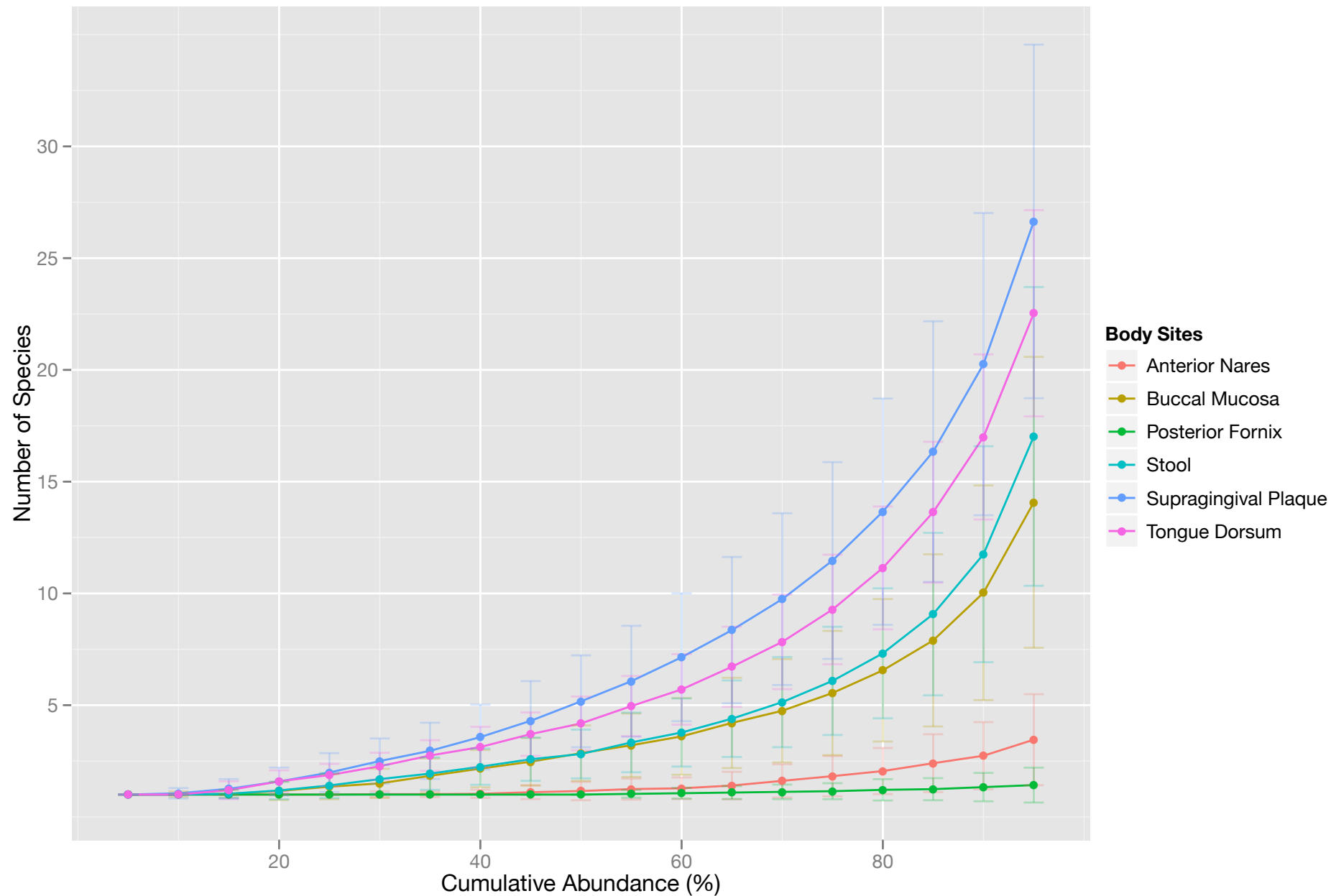

Supplement: Figure S6 — Top species by abundance. This line graph shows how many species on average it takes to constitute a percentage of the total abundance in a sample, ranging from 5% to 95%. Strains were summed to calculate the total for each species, and the error bars represent the standard deviation. On average, 74, 154, 90, 92, 277 and 255 species are needed for 100% in the anterior nares, buccal mucosa, posterior fornix, stool, supragingival plaque and tongue dorsum, respectively. (PDF) [file pone.0097279.s006.pdf]

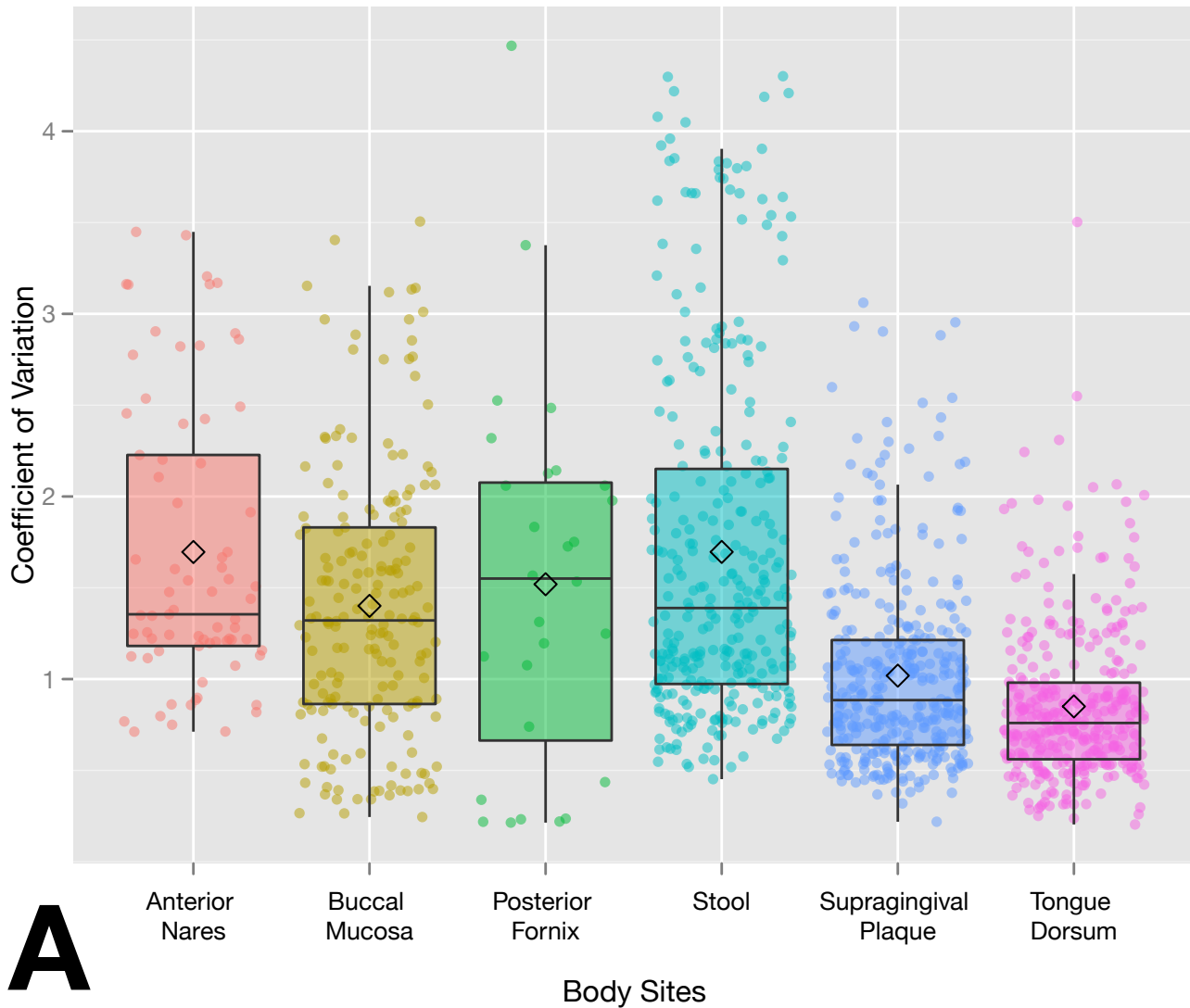

# Buccal Mucosa

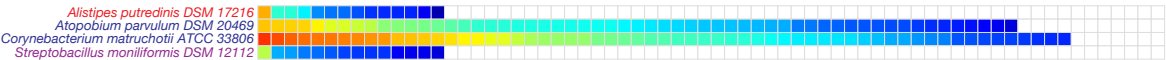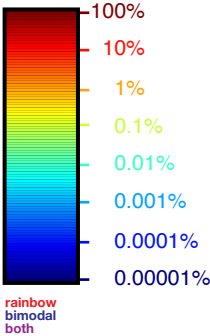

# Posterior Fornix

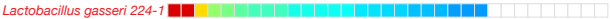

# Stool

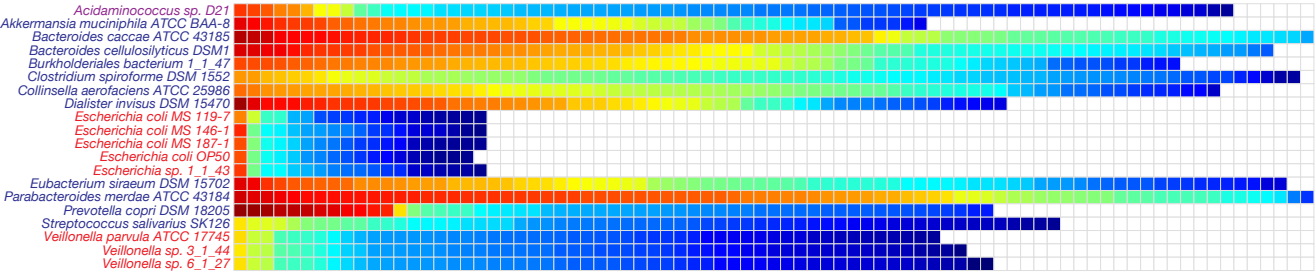

# Supragingival Plaque

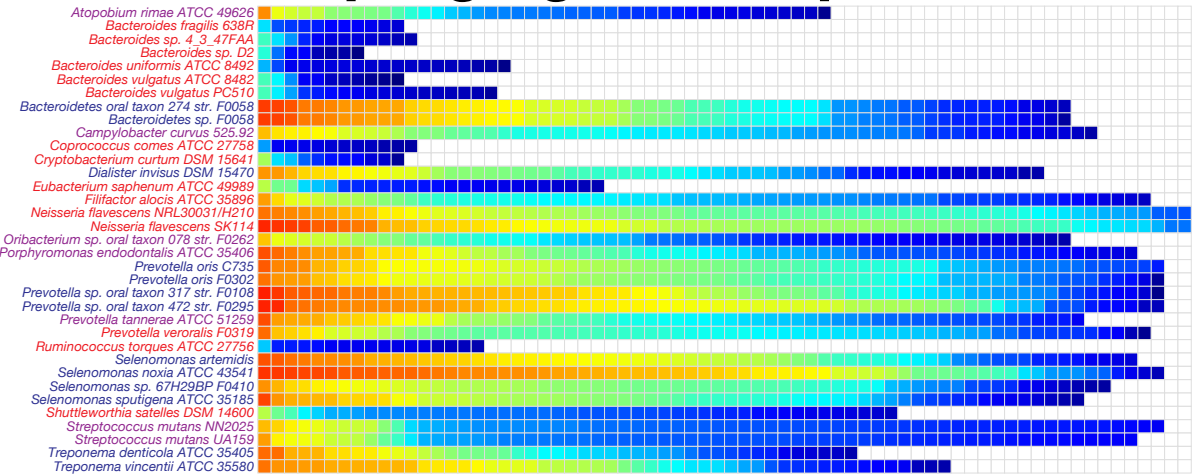

# Tongue Dorsum

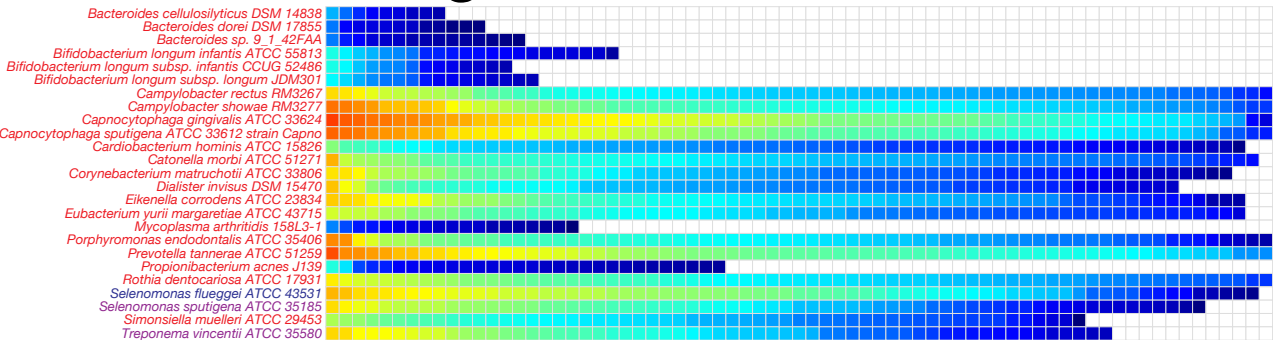

B

Supplement: Figure S8 — Variability in strain abundance across samples. (A) Distribution of the coefficient of variation (CV) for each strain across the samples for each individual body site. The distribution shows that the variation of the abundances of individual strains is the lowest in tongue dorsum and the highest in the posterior fornix (based on the median), while the distribution is the widest for the stool. The median diversity is indicated by a horizontal line in the box (covering the 25th until the 75th percentile), the diamond represents the average and the outliers are visualized using dots. The whiskers of the box are the lowest and highest observation of variation. The points that make up the distribution are plotted in the color of the body site. The points beyond the whiskers are considered outliers and define the rainbow strains (see methods). (B) Strains that show an unusually variable pattern across multiple samples based on two metrics. The samples are ordered by their relative abundance to show the variable distribution. The strains are color coded to indicate the metric by which they are considered to be unusually variable (red for rainbow strains, blue for bimodal strains, and purple for both metrics). (PDF) [file pone.0097279.s008.pdf]
