# Supplementary material for: The Prevalence of Species and Strains in the Human Microbiome: A Resource for Experimental Efforts
Source: PLoS One. 2014 May 14;9(5):e97279. doi: 10.1371/journal.pone.0097279 (PMC4020798; doi:10.1371/journal.pone.0097279)

Anterior Nares

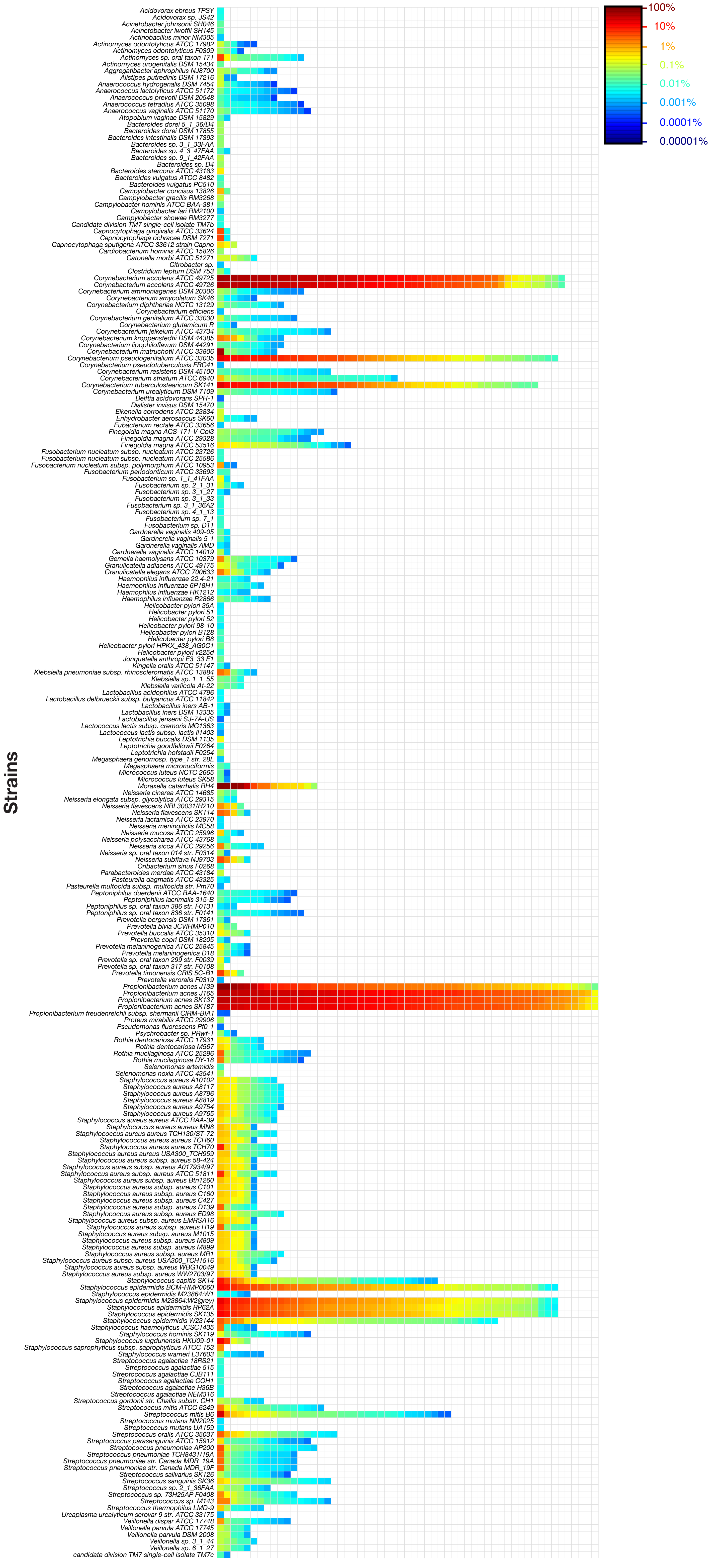

Buccal Mucosa

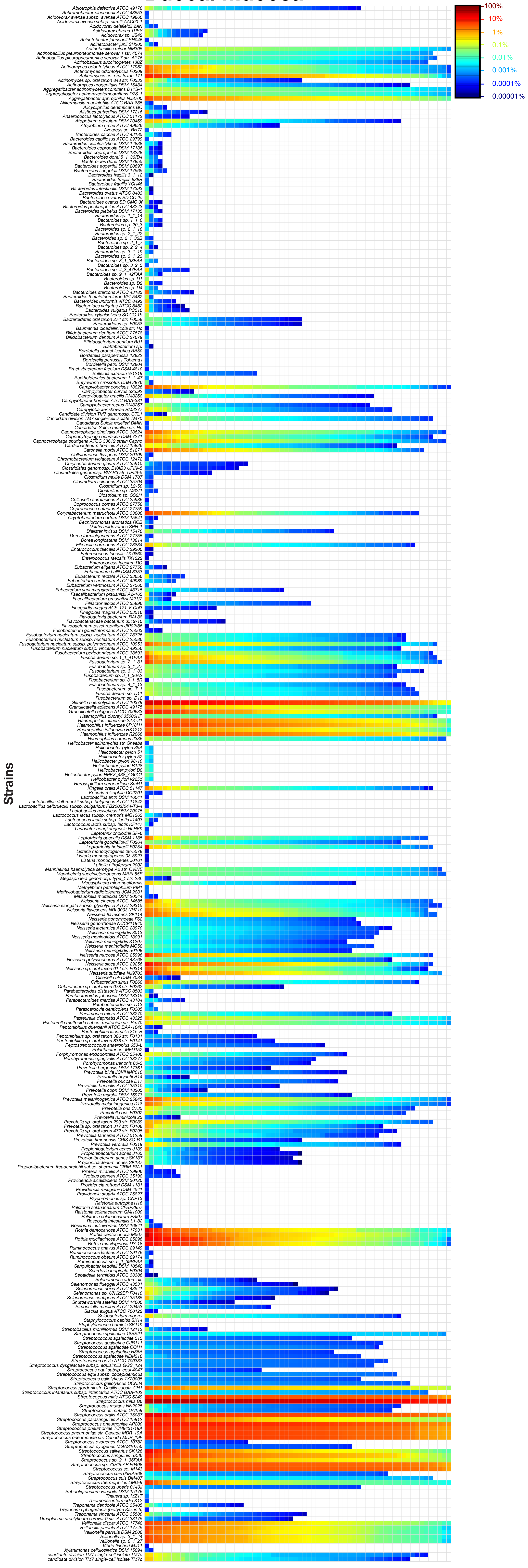

Stool

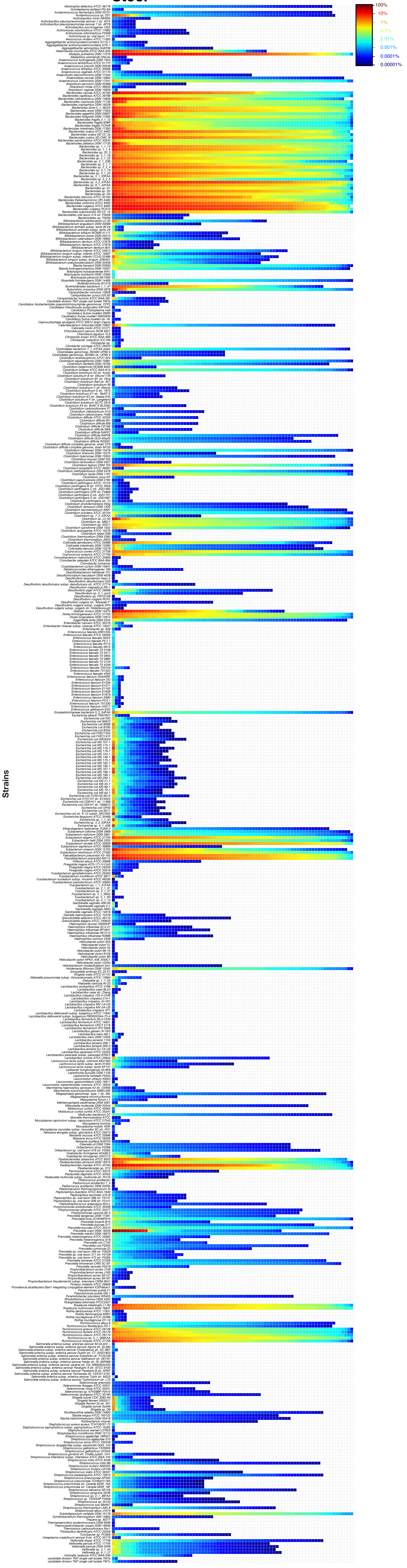

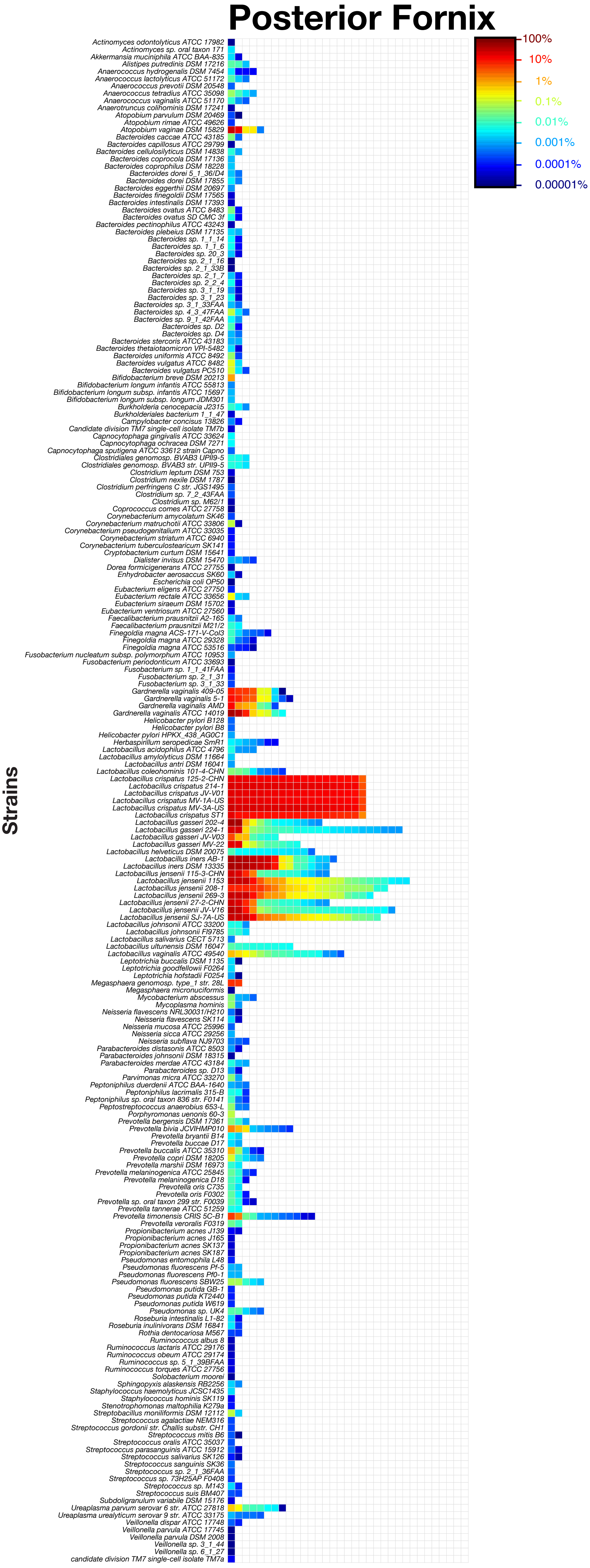
Posterior Fornix
100%
10%
1%
0.1%
0.01%
0.001%
0.0001%
0.00001%
Strains
Actinomyces odontolyticus ATCC 17982
Actinomyces sp. oral taxon 171
Akkermansia muciniphila ATCC BAA-835
Alistipes putredinis DSM 17216
Anaerococcus hydrogenalis DSM 7454
Anaerococcus lactolyticus ATCC 51172
Anaerococcus prevotii DSM 20548
Anaerococcus tetradius ATCC 35098
Anaerococcus vaginalis ATCC 51170
Anaerotruncus colihominis DSM 17241
Atopobium parvulum DSM 20469
Atopobium rimae ATCC 49626
Atopobium vaginae DSM 15829
Bacteroides caccae ATCC 43185
Bacteroides capillosus ATCC 29799
Bacteroides cellulosilyticus DSM 14838
Bacteroides coprocola DSM 17136
Bacteroides coprophilus DSM 18228
Bacteroides dorei 5_1_36/D4
Bacteroides dorei DSM 17855
Bacteroides eggerthii DSM 20697
Bacteroides finegoldii DSM 17565
Bacteroides intestinalis DSM 17393
Bacteroides ovatus ATCC 8483
Bacteroides ovatus SD CMC 3f
Bacteroides pectinophilus ATCC 43243
Bacteroides plebeius DSM 17135
Bacteroides sp. 1_1_14
Bacteroides sp. 1_1_6
Bacteroides sp. 20_3
Bacteroides sp. 2_1_16
Bacteroides sp. 2_1_33B
Bacteroides sp. 2_1_7
Bacteroides sp. 2_2_4
Bacteroides sp. 3_1_19
Bacteroides sp. 3_1_23
Bacteroides sp. 3_1_33FAA
Bacteroides sp. 4_3_47FAA
Bacteroides sp. 9_1_42FAA
Bacteroides sp. D2
Bacteroides sp. D4
Bacteroides stercoris ATCC 43183
Bacteroides thetaiotaomicron VPI-5482
Bacteroides uniformis ATCC 8492
Bacteroides vulgatus ATCC 8482
Bacteroides vulgatus PC510
Bifidobacterium breve DSM 20213
Bifidobacterium longum infantis ATCC 55813
Bifidobacterium longum subsp. infantis ATCC 15697
Bifidobacterium longum subsp. longum JDM301
Burkholderia cenocepacia J2315
Burkholderiales bacterium 1_1_47
Campylobacter concisus 13826
Candidate division TM7 single-cell isolate TM7b
Capnocytophaga gingivalis ATCC 33624
Capnocytophaga ochracea DSM 7271
Capnocytophaga sputigena ATCC 33612 strain Capno
Clostridiales genomosp. BVAB3 UPII9-5
Clostridiales genomosp. BVAB3 str. UPII9-5
Clostridium leptum DSM 753
Clostridium nexile DSM 1787
Clostridium perfringens C str. JGS1495
Clostridium sp. 7_2_43FAA
Clostridium sp. M62/1
Coprococcus comes ATCC 27758
Corynebacterium amycolatum SK46
Corynebacterium matruchotii ATCC 33806
Corynebacterium pseudogenitalium ATCC 33035
Corynebacterium striatum ATCC 6940
Corynebacterium tuberculostearicum SK141
Cryptobacterium curtum DSM 15641
Dialister invisus DSM 15470
Dorea formicigenerans ATCC 27755
Enhydrobacter aerosaccus SK60
Escherichia coli OP50
Eubacterium eligens ATCC 27750
Eubacterium rectale ATCC 33656
Eubacterium siraeum DSM 15702
Eubacterium ventriosum ATCC 27560
Faecalibacterium prausnitzii A2-165
Faecalibacterium prausnitzii M21/2
Finegoldia magna ACS-171-V-Col3
Finegoldia magna ATCC 29328
Finegoldia magna ATCC 53516
Fusobacterium nucleatum subsp. polymorphum ATCC 10953
Fusobacterium periodonticum ATCC 33693
Fusobacterium sp. 1_1_41FAA
Fusobacterium sp. 2_1_31
Fusobacterium sp. 3_1_33
Gardnerella vaginalis 409-05
Gardnerella vaginalis 5-1
Gardnerella vaginalis AMD
Gardnerella vaginalis ATCC 14019
Helicobacter pylori B128
Helicobacter pylori B8
Helicobacter pylori HPKX_438_AG0C1
Herbaspirillum seropedicae SmR1
Lactobacillus acidophilus ATCC 4796
Lactobacillus amylolyticus DSM 11664
Lactobacillus antri DSM 16041
Lactobacillus coleohominis 101-4-CHN
Lactobacillus crispatus 125-2-CHN
Lactobacillus crispatus 214-1
Lactobacillus crispatus JV-V01
Lactobacillus crispatus MV-1A-US
Lactobacillus crispatus MV-3A-US
Lactobacillus crispatus ST1
Lactobacillus gasseri 202-4
Lactobacillus gasseri 224-1
Lactobacillus gasseri JV-V03
Lactobacillus gasseri MV-22
Lactobacillus helveticus DSM 20075
Lactobacillus iners AB-1
Lactobacillus iners DSM 13335
Lactobacillus jensenii 115-3-CHN
Lactobacillus jensenii 1153
Lactobacillus jensenii 208-1
Lactobacillus jensenii 269-3
Lactobacillus jensenii 27-2-CHN
Lactobacillus jensenii JV-V16
Lactobacillus jensenii SJ-7A-US
Lactobacillus johnsonii ATCC 33200
Lactobacillus johnsonii FI9785
Lactobacillus salivarius CECT 5713
Lactobacillus ultunensis DSM 16047
Lactobacillus vaginalis ATCC 49540
Leptotrichia buccalis DSM 1135
Leptotrichia goodfellowii F0264
Leptotrichia hofstadii F0254
Megasphaera genomosp. type_1 str. 28L
Megasphaera micronuciformis
Mycobacterium abscessus
Mycoplasma hominis
Neisseria flavescens NRL30031/H210
Neisseria flavescens SK114
Neisseria mucosa ATCC 25996
Neisseria sicca ATCC 29256
Neisseria subflava NJ9703
Parabacteroides distasonis ATCC 8503
Parabacteroides johnsonii DSM 18315
Parabacteroides merdae ATCC 43184
Parabacteroides sp. D13
Parvimonas micra ATCC 33270
Peptoniphilus duerdenii ATCC BAA-1640
Peptoniphilus lacrimalis 315-B
Peptoniphilus sp. oral taxon 836 str. F0141
Peptostreptococcus anaerobius 653-L
Porphyromonas uenonis 60-3
Prevotella bergensis DSM 17361
Prevotella bivia JCVIHMP010
Prevotella bryantii B14
Prevotella buccae D17
Prevotella buccalis ATCC 35310
Prevotella copri DSM 18205
Prevotella marshii DSM 16973
Prevotella melaninogenica ATCC 25845
Prevotella melaninogenica D18
Prevotella oris C735
Prevotella oris F0302
Prevotella sp. oral taxon 299 str. F0039
Prevotella tannerae ATCC 51259
Prevotella timonensis CRIS 5C-B1
Prevotella veroralis F0319
Propionibacterium acnes J139
Propionibacterium acnes J165
Propionibacterium acnes SK137
Propionibacterium acnes SK187
Pseudomonas entomophila L48
Pseudomonas fluorescens Pf-5
Pseudomonas fluorescens Pf0-1
Pseudomonas fluorescens SBW25
Pseudomonas putida GB-1
Pseudomonas putida KT2440
Pseudomonas putida W619
Pseudomonas sp. UK4
Roseburia intestinalis L1-82
Roseburia inulinivorans DSM 16841
Rothia dentocariosa M567
Ruminococcus albus 8
Ruminococcus lactaris ATCC 29176
Ruminococcus obeum ATCC 29174
Ruminococcus sp. 5_1_39BFAA
Ruminococcus torques ATCC 27756
Solobacterium moorei
Sphingopyxis alaskensis RB2256
Staphylococcus haemolyticus JCSC1435
Staphylococcus hominis SK119
Stenotrophomonas maltophilia K279a
Streptobacillus moniliformis DSM 12112
Streptococcus agalactiae NEM316
Streptococcus gordonii str. Challis substr. CH1
Streptococcus mitis B6
Streptococcus oralis ATCC 35037
Streptococcus parasanguinis ATCC 15912
Streptococcus salivarius SK126
Streptococcus sanguinis SK36
Streptococcus sp. 2_1_36FAA
Streptococcus sp. 73H25AP F0408
Streptococcus sp. M143
Streptococcus suis BM407
Subdoligranulum variabile DSM 15176
Ureaplasma parvum serovar 6 str. ATCC 27818
Ureaplasma urealyticum serovar 9 str. ATCC 33175
Veillonella dispar ATCC 17748
Veillonella parvula ATCC 17745
Veillonella parvula DSM 2008
Veillonella sp. 3_1_44
Veillonella sp. 6_1_27
candidate division TM7 single-cell isolate TM7a

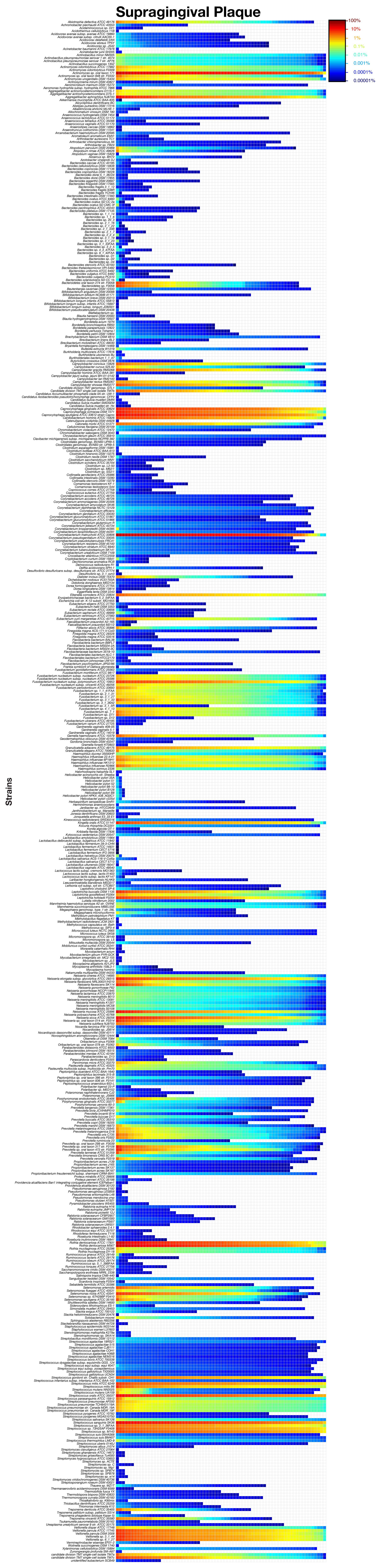

Supragingival Plaque
100%
10%
1%
0.1%
0.01%
0.001%
0.0001%
0.00001%
Strains

Tongue Dorsum

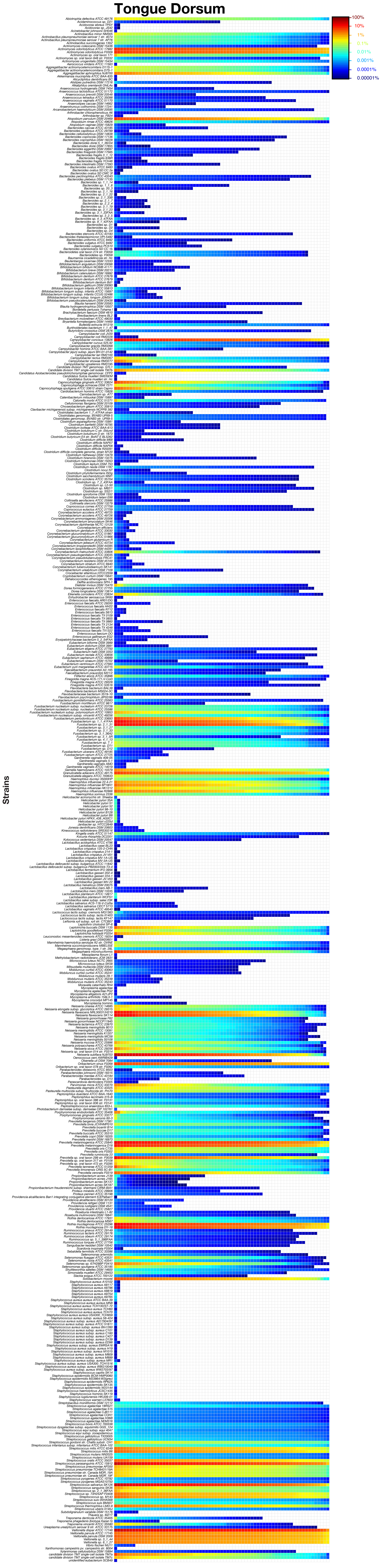

Supplement: Figure S4 — Heat maps of ordered strain abundance. Heat map visualization of the relative abundances with the samples ordered by the relative abundance for each strain. Species are listed alphabetically. (PDF) [file pone.0097279.s004.pdf]
