# Supplementary material for: The Prevalence of Species and Strains in the Human Microbiome: A Resource for Experimental Efforts
Source: PLoS One. 2014 May 14;9(5):e97279. doi: 10.1371/journal.pone.0097279 (PMC4020798; doi:10.1371/journal.pone.0097279)

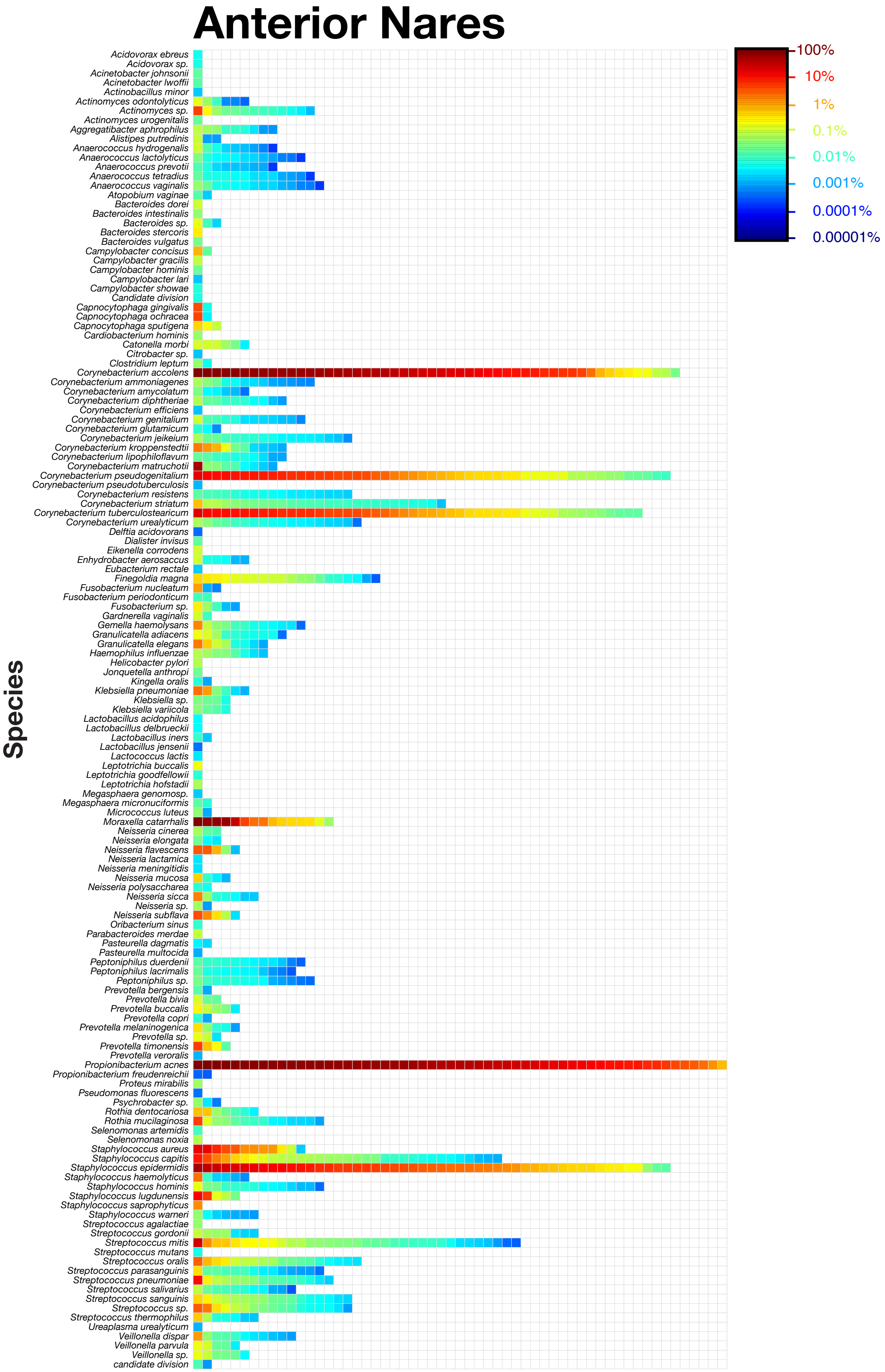
Anterior Nares
Species
100%
10%
1%
0.1%
0.01%
0.001%
0.0001%
0.00001%
Acidovorax ebreus
Acidovorax sp.
Acinetobacter johnsonii
Acinetobacter lwoffii
Actinobacillus minor
Actinomyces odontolyticus
Actinomyces sp.
Actinomyces urogenitalis
Aggregatibacter aphrophilus
Alistipes putredinis
Anaerococcus hydrogenalis
Anaerococcus lactolyticus
Anaerococcus prevotii
Anaerococcus tetradius
Anaerococcus vaginalis
Atopobium vaginae
Bacteroides dorei
Bacteroides intestinalis
Bacteroides sp.
Bacteroides stercoris
Bacteroides vulgatus
Campylobacter concisus
Campylobacter gracilis
Campylobacter hominis
Campylobacter lari
Campylobacter showae
Candidate division
Capnocytophaga gingivalis
Capnocytophaga ochracea
Capnocytophaga sputigena
Cardiobacterium hominis
Catonella morbi
Citrobacter sp.
Clostridium leptum
Corynebacterium accolens
Corynebacterium ammoniagenes
Corynebacterium amycolatum
Corynebacterium diphtheriae
Corynebacterium efficiens
Corynebacterium genitalium
Corynebacterium glutamicum
Corynebacterium jeikeium
Corynebacterium kroppenstedtii
Corynebacterium lipophiloflavum
Corynebacterium matruchotii
Corynebacterium pseudogenitalium
Corynebacterium pseudotuberculosis
Corynebacterium resistens
Corynebacterium striatum
Corynebacterium tuberculostearicum
Corynebacterium urealyticum
Delftia acidovorans
Dialister invisus
Eikenella corrodens
Enhydrobacter aerosaccus
Eubacterium rectale
Finegoldia magna
Fusobacterium nucleatum
Fusobacterium periodonticum
Fusobacterium sp.
Gardnerella vaginalis
Gemella haemolysans
Granulicatella adiacens
Granulicatella elegans
Haemophilus influenzae
Helicobacter pylori
Jonquetella anthropi
Kingella oralis
Klebsiella pneumoniae
Klebsiella sp.
Klebsiella variicola
Lactobacillus acidophilus
Lactobacillus delbrueckii
Lactobacillus iners
Lactobacillus jensenii
Lactococcus lactis
Leptotrichia buccalis
Leptotrichia goodfellowii
Leptotrichia hofstadii
Megasphaera genomosp.
Megasphaera micronuciformis
Micrococcus luteus
Moraxella catarrhalis
Neisseria cinerea
Neisseria elongata
Neisseria flavescens
Neisseria lactamica
Neisseria meningitidis
Neisseria mucosa
Neisseria polysaccharea
Neisseria sicca
Neisseria sp.
Neisseria subflava
Oribacterium sinus
Parabacteroides merdae
Pasteurella dagmatis
Pasteurella multocida
Peptoniphilus duerdenii
Peptoniphilus lacrimalis
Peptoniphilus sp.
Prevotella bergensis
Prevotella bivia
Prevotella buccalis
Prevotella copri
Prevotella melaninogenica
Prevotella sp.
Prevotella timonensis
Prevotella veroralis
Propionibacterium acnes
Propionibacterium freudenreichii
Proteus mirabilis
Pseudomonas fluorescens
Psychrobacter sp.
Rothia dentocariosa
Rothia mucilaginosa
Selenomonas artemidis
Selenomonas noxia
Staphylococcus aureus
Staphylococcus capitis
Staphylococcus epidermidis
Staphylococcus haemolyticus
Staphylococcus hominis
Staphylococcus lugdunensis
Staphylococcus saprophyticus
Staphylococcus warneri
Streptococcus agalactiae
Streptococcus gordonii
Streptococcus mitis
Streptococcus mutans
Streptococcus oralis
Streptococcus parasanguinis
Streptococcus pneumoniae
Streptococcus salivarius
Streptococcus sanguinis
Streptococcus sp.
Streptococcus thermophilus
Ureaplasma urealyticum
Veillonella dispar
Veillonella parvula
Veillonella sp.
candidate division

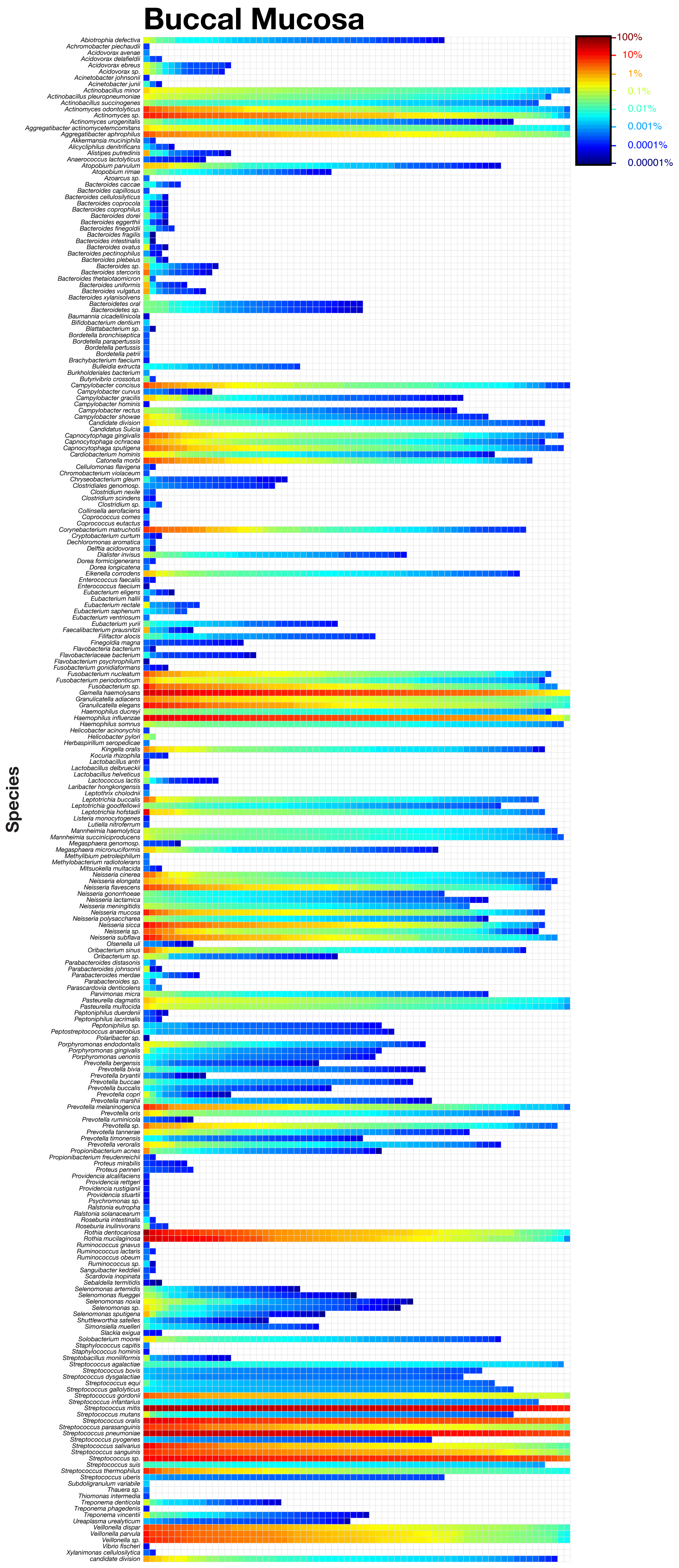

Buccal Mucosa
Species
100%
10%
1%
0.1%
0.01%
0.001%
0.0001%
0.00001%
Abiotrophia defectiva
Achromobacter piechaudii
Acidovorax avenae
Acidovorax delafieldii
Acidovorax ebreus
Acidovorax sp.
Acinetobacter johnsonii
Acinetobacter junii
Actinobacillus minor
Actinobacillus pleuropneumoniae
Actinobacillus succinogenes
Actinomyces odontolyticus
Actinomyces sp.
Actinomyces urogenitalis
Aggregatibacter actinomycetemcomitans
Aggregatibacter aphrophilus
Akkermansia muciniphila
Alicycliphilus denitrificans
Alistipes putredinis
Anaerococcus lactolyticus
Atopobium parvulum
Atopobium rimae
Azoarcus sp.
Bacteroides caccae
Bacteroides capillosus
Bacteroides cellulosilyticus
Bacteroides coprocola
Bacteroides coprophilus
Bacteroides dorei
Bacteroides eggerthii
Bacteroides finegoldii
Bacteroides fragilis
Bacteroides intestinalis
Bacteroides ovatus
Bacteroides pectinophilus
Bacteroides plebeius
Bacteroides sp.
Bacteroides stercoris
Bacteroides thetaiotaomicron
Bacteroides uniformis
Bacteroides vulgatus
Bacteroides xylanisolvens
Bacteroidetes oral
Bacteroidetes sp.
Baumannia cicadellinicola
Bifidobacterium dentium
Blattabacterium sp.
Bordetella bronchiseptica
Bordetella parapertussis
Bordetella pertussis
Bordetella petrii
Brachybacterium faecium
Bulleidia extructa
Burkholderiales bacterium
Butyrivibrio crossotus
Campylobacter concisus
Campylobacter curvus
Campylobacter gracilis
Campylobacter hominis
Campylobacter rectus
Campylobacter showae
Candidate division
Candidatus Sulcia
Capnocytophaga gingivalis
Capnocytophaga ochracea
Capnocytophaga sputigena
Cardiobacterium hominis
Catonella morbi
Cellulomonas flavigena
Chromobacterium violaceum
Chryseobacterium gleum
Clostridiales genomosp.
Clostridium nexile
Clostridium scindens
Clostridium sp.
Collinsella aerofaciens
Coprococcus comes
Coprococcus eutactus
Corynebacterium matruchotii
Cryptobacterium curtum
Dechloromonas aromatica
Delftia acidovorans
Dialister invisus
Dorea formicigenerans
Dorea longicatena
Eikenella corrodens
Enterococcus faecalis
Enterococcus faecium
Eubacterium eligens
Eubacterium hallii
Eubacterium rectale
Eubacterium saphenum
Eubacterium ventriosum
Eubacterium yurii
Faecalibacterium prausnitzii
Filifactor alocis
Finegoldia magna
Flavobacteria bacterium
Flavobacteriaceae bacterium
Flavobacterium psychrophilum
Fusobacterium gonidiaformans
Fusobacterium nucleatum
Fusobacterium periodonticum
Fusobacterium sp.
Gemella haemolysans
Granulicatella adiacens
Granulicatella elegans
Haemophilus ducreyi
Haemophilus influenzae
Haemophilus somnus
Helicobacter acinonychis
Helicobacter pylori
Herbaspirillum seropedicae
Kingella oralis
Kocuria rhizophila
Lactobacillus antri
Lactobacillus delbrueckii
Lactobacillus helveticus
Lactococcus lactis
Laribacter hongkongensis
Leptothrix cholodnii
Leptotrichia buccalis
Leptotrichia goodfellowii
Leptotrichia hofstadii
Listeria monocytogenes
Lutiella nitroferrum
Mannheimia haemolytica
Mannheimia succiniciproducens
Megasphaera genomosp.
Megasphaera micronuciformis
Methylibium petroleiphilum
Methylobacterium radiotolerans
Mitsuokella multacida
Neisseria cinerea
Neisseria elongata
Neisseria flavescens
Neisseria gonorrhoeae
Neisseria lactamica
Neisseria meningitidis
Neisseria mucosa
Neisseria polysaccharea
Neisseria sicca
Neisseria sp.
Neisseria subflava
Olsenella uli
Oribacterium sinus
Oribacterium sp.
Parabacteroides distasonis
Parabacteroides johnsonii
Parabacteroides merdae
Parabacteroides sp.
Parascardovia denticolens
Parvimonas micra
Pasteurella dagmatis
Pasteurella multocida
Peptoniphilus duerdenii
Peptoniphilus lacrimalis
Peptoniphilus sp.
Peptostreptococcus anaerobius
Polaribacter sp.
Porphyromonas endodontalis
Porphyromonas gingivalis
Porphyromonas uenonis
Prevotella bergensis
Prevotella bivia
Prevotella bryantii
Prevotella buccae
Prevotella buccalis
Prevotella copri
Prevotella marshii
Prevotella melaninogenica
Prevotella oris
Prevotella ruminicola
Prevotella sp.
Prevotella tannerae
Prevotella timonensis
Prevotella veroralis
Propionibacterium acnes
Propionibacterium freudenreichii
Proteus mirabilis
Proteus penneri
Providencia alcalifaciens
Providencia rettgeri
Providencia rustigianii
Providencia stuartii
Psychromonas sp.
Ralstonia eutropha
Ralstonia solanacearum
Roseburia intestinalis
Roseburia inulinivorans
Rothia dentocariosa
Rothia mucilaginosa
Ruminococcus gnavus
Ruminococcus lactaris
Ruminococcus obeum
Ruminococcus sp.
Sanguibacter keddieii
Scardovia inopinata
Sebaldella termitidis
Selenomonas artemidis
Selenomonas flueggei
Selenomonas noxia
Selenomonas sp.
Selenomonas sputigena
Shuttleworthia satelles
Simonsiella muelleri
Slackia exigua
Solobacterium moorei
Staphylococcus capitis
Staphylococcus hominis
Streptobacillus moniliformis
Streptococcus agalactiae
Streptococcus bovis
Streptococcus dysgalactiae
Streptococcus equi
Streptococcus gallolyticus
Streptococcus gordonii
Streptococcus infantarius
Streptococcus mitis
Streptococcus mutans
Streptococcus oralis
Streptococcus parasanguinis
Streptococcus pneumoniae
Streptococcus pyogenes
Streptococcus salivarius
Streptococcus sanguinis
Streptococcus sp.
Streptococcus suis
Streptococcus thermophilus
Streptococcus uberis
Subdoligranulum variabile
Thauera sp.
Thiomonas intermedia
Treponema denticola
Treponema phagedenis
Treponema vincentii
Ureaplasma urealyticum
Veillonella dispar
Veillonella parvula
Veillonella sp.
Vibrio fischeri
Xylanimonas cellulosilytica
candidate division

Posterior Fornix

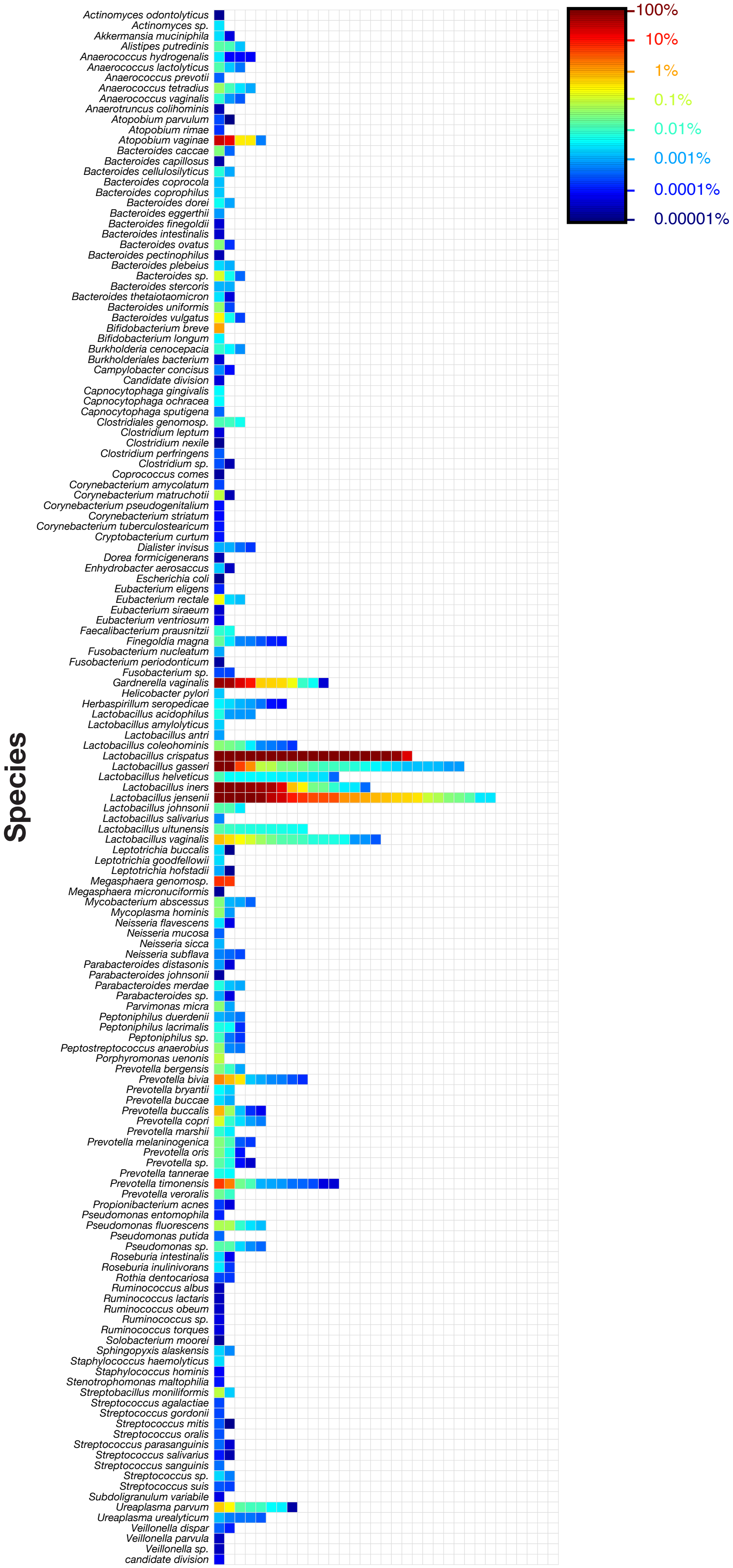

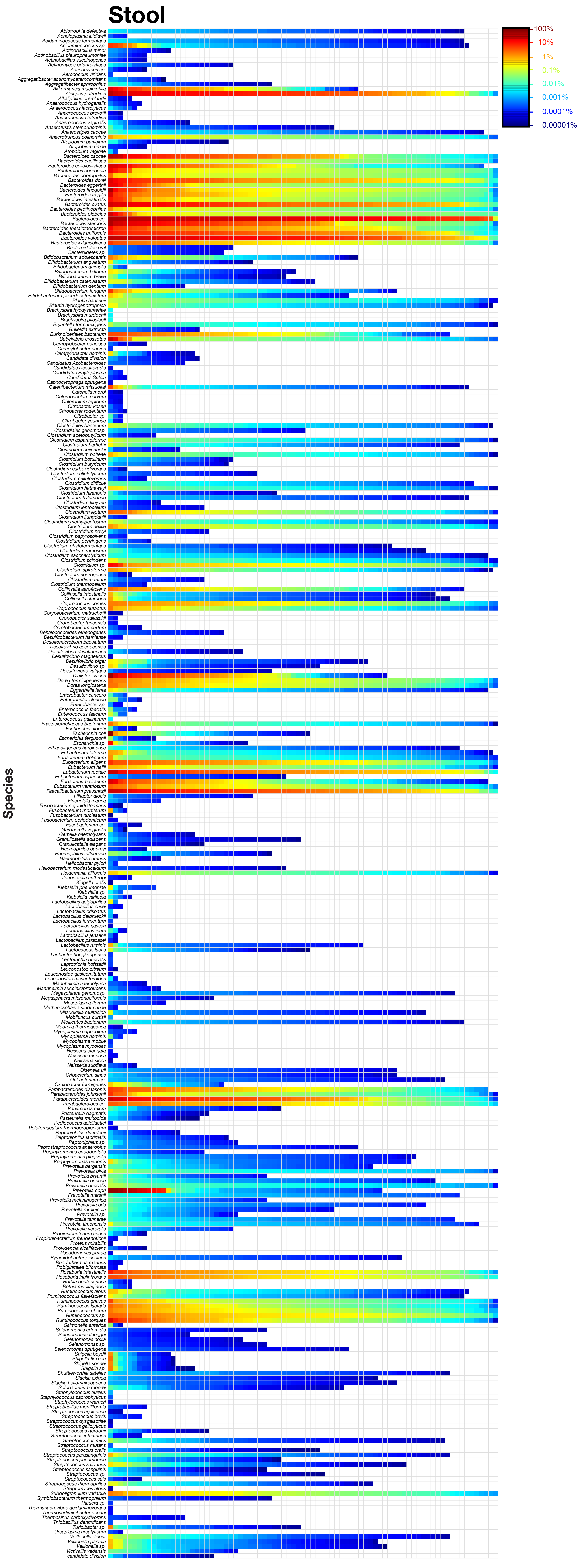
Stool
100%
10%
1%
0.1%
0.01%
0.001%
0.0001%
0.00001%
Species
Abiotrophia defectiva
Acholeplasma laidlawii
Acidaminococcus fermentans
Acidaminococcus sp.
Actinobacillus minor
Actinobacillus pleuropneumoniae
Actinobacillus succinogenes
Actinomyces odontolyticus
Actinomyces sp.
Aerococcus viridans
Aggregatibacter actinomycetemcomitans
Aggregatibacter aphrophilus
Akkermansia muciniphila
Alistipes putredinis
Alkaliphilus oremlandii
Anaerococcus hydrogenalis
Anaerococcus lactolyticus
Anaerococcus prevotii
Anaerococcus tetradius
Anaerococcus vaginalis
Anaerofustis stercorihominis
Anaerostipes caccae
Anaerotruncus colihominis
Atopobium parvulum
Atopobium rimae
Atopobium vaginae
Bacteroides caccae
Bacteroides capillosus
Bacteroides cellulosilyticus
Bacteroides coprocola
Bacteroides coprophilus
Bacteroides dorei
Bacteroides eggerthii
Bacteroides finegoldii
Bacteroides fragilis
Bacteroides intestinalis
Bacteroides ovatus
Bacteroides pectinophilus
Bacteroides plebeius
Bacteroides sp.
Bacteroides stercoris
Bacteroides thetaiotaomicron
Bacteroides uniformis
Bacteroides vulgatus
Bacteroides xylanisolvens
Bacteroidetes oral
Bacteroidetes sp.
Bifidobacterium adolescentis
Bifidobacterium angulatum
Bifidobacterium animalis
Bifidobacterium bifidum
Bifidobacterium breve
Bifidobacterium catenulatum
Bifidobacterium dentium
Bifidobacterium longum
Bifidobacterium pseudocatenulatum
Blautia hansenii
Blautia hydrogenotrophica
Brachyspira hyodysenteriae
Brachyspira murdochii
Brachyspira pilosicoli
Bryantella formatexigens
Bulleidia extructa
Burkholderiales bacterium
Butyrivibrio crossotus
Campylobacter concisus
Campylobacter curvus
Campylobacter hominis
Candidate division
Candidatus Azobacteroides
Candidatus Desulforudis
Candidatus Phytoplasma
Candidatus Sulcia
Capnocytophaga sputigena
Catenibacterium mitsuokai
Catonella morbi
Chlorobaculum parvum
Chlorobium tepidum
Citrobacter koseri
Citrobacter rodentium
Citrobacter sp.
Citrobacter youngae
Clostridiales bacterium
Clostridiales genomosp.
Clostridium acetobutylicum
Clostridium asparagiforme
Clostridium bartlettii
Clostridium beijerinckii
Clostridium bolteae
Clostridium botulinum
Clostridium butyricum
Clostridium carboxidivorans
Clostridium cellulolyticum
Clostridium cellulovorans
Clostridium difficile
Clostridium hathewayi
Clostridium hiranonis
Clostridium hylemonae
Clostridium kluyveri
Clostridium lentocellum
Clostridium leptum
Clostridium ljungdahlii
Clostridium methylpentosum
Clostridium nexile
Clostridium novyi
Clostridium papyrosolvens
Clostridium perfringens
Clostridium phytofermentans
Clostridium ramosum
Clostridium saccharolyticum
Clostridium scindens
Clostridium sp.
Clostridium spiroforme
Clostridium sporogenes
Clostridium tetani
Clostridium thermocellum
Collinsella aerofaciens
Collinsella intestinalis
Collinsella stercoris
Coprococcus comes
Coprococcus eutactus
Corynebacterium matruchotii
Cronobacter sakazakii
Cronobacter turicensis
Cryptobacterium curtum
Dehalococcoides ethenogenes
Desulfitobacterium hafniense
Desulfomicrobium baculatum
Desulfovibrio aespoeensis
Desulfovibrio desulfuricans
Desulfovibrio magneticus
Desulfovibrio piger
Desulfovibrio sp.
Desulfovibrio vulgaris
Dialister invisus
Dorea formicigenerans
Dorea longicatena
Eggerthella lenta
Enterobacter cancero
Enterobacter cloacae
Enterobacter sp.
Enterococcus faecalis
Enterococcus faecium
Enterococcus gallinarum
Erysipelotrichaceae bacterium
Escherichia albertii
Escherichia coli
Escherichia fergusonii
Escherichia sp.
Ethanoligenens harbinense
Eubacterium biforme
Eubacterium dolichum
Eubacterium eligens
Eubacterium hallii
Eubacterium rectale
Eubacterium saphenum
Eubacterium siraeum
Eubacterium ventriosum
Faecalibacterium prausnitzii
Filifactor alocis
Finegoldia magna
Fusobacterium gonidiaformans
Fusobacterium mortiferum
Fusobacterium nucleatum
Fusobacterium periodonticum
Fusobacterium sp.
Gardnerella vaginalis
Gemella haemolysans
Granulicatella adiacens
Granulicatella elegans
Haemophilus ducreyi
Haemophilus influenzae
Haemophilus somnus
Helicobacter pylori
Heliobacterium modesticaldum
Holdemania filiformis
Jonquetella anthropi
Kingella oralis
Klebsiella pneumoniae
Klebsiella sp.
Klebsiella variicola
Lactobacillus acidophilus
Lactobacillus casei
Lactobacillus crispatus
Lactobacillus delbrueckii
Lactobacillus fermentum
Lactobacillus gasseri
Lactobacillus iners
Lactobacillus jensenii
Lactobacillus paracasei
Lactobacillus ruminis
Lactococcus lactis
Laribacter hongkongensis
Leptotrichia buccalis
Leptotrichia hofstadii
Leuconostoc citreum
Leuconostoc gasicomitatum
Leuconostoc mesenteroides
Mannheimia haemolytica
Mannheimia succiniciproducens
Megasphaera genomosp.
Megasphaera micronuciformis
Mesoplasma florum
Methanosphaera stadtmanae
Mitsuokella multacida
Mobiluncus curtisii
Mollicutes bacterium
Moorella thermoacetica
Mycoplasma capricolum
Mycoplasma hominis
Mycoplasma mobile
Mycoplasma mycoides
Neisseria elongata
Neisseria mucosa
Neisseria sicca
Neisseria subflava
Olsenella uli
Oribacterium sinus
Oribacterium sp.
Oxalobacter formigenes
Parabacteroides distasonis
Parabacteroides johnsonii
Parabacteroides merdae
Parabacteroides sp.
Parvimonas micra
Pasteurella dagmatis
Pasteurella multocida
Pediococcus acidilactici
Pelotomaculum thermopropionicum
Peptoniphilus duerdenii
Peptoniphilus lacrimalis
Peptoniphilus sp.
Peptostreptococcus anaerobius
Porphyromonas endodontalis
Porphyromonas gingivalis
Porphyromonas uenonis
Prevotella bergensis
Prevotella bivia
Prevotella bryantii
Prevotella buccae
Prevotella buccalis
Prevotella copri
Prevotella marshii
Prevotella melaninogenica
Prevotella oris
Prevotella ruminicola
Prevotella sp.
Prevotella tannerae
Prevotella timonensis
Prevotella veroralis
Propionibacterium acnes
Propionibacterium freudenreichii
Proteus mirabilis
Providencia alcalifaciens
Pseudomonas putida
Pyramidobacter piscolens
Rhodothermus marinus
Robiginitalea biformata
Roseburia intestinalis
Roseburia inulinivorans
Rothia dentocariosa
Rothia mucilaginosa
Ruminococcus albus
Ruminococcus flavefaciens
Ruminococcus gnavus
Ruminococcus lactaris
Ruminococcus obeum
Ruminococcus sp.
Ruminococcus torques
Salmonella enterica
Selenomonas artemidis
Selenomonas flueggei
Selenomonas noxia
Selenomonas sp.
Selenomonas sputigena
Shigella boydii
Shigella flexneri
Shigella sonnei
Shigella sp.
Shuttleworthia satelles
Slackia exigua
Slackia heliotrinireducens
Solobacterium moorei
Staphylococcus aureus
Staphylococcus saprophyticus
Staphylococcus warneri
Streptobacillus moniliformis
Streptococcus agalactiae
Streptococcus bovis
Streptococcus dysgalactiae
Streptococcus gallolyticus
Streptococcus gordonii
Streptococcus infantarius
Streptococcus mitis
Streptococcus mutans
Streptococcus oralis
Streptococcus parasanguinis
Streptococcus pneumoniae
Streptococcus salivarius
Streptococcus sanguinis
Streptococcus sp.
Streptococcus suis
Streptococcus thermophilus
Streptomyces albus
Subdoligranulum variabile
Symbiobacterium thermophilum
Thauera sp.
Thermanaerovibrio acidaminovorans
Thermosediminibacter oceani
Thermosinus carboxydivorans
Thiobacillus denitrificans
Turicibacter sp.
Ureaplasma urealyticum
Veillonella dispar
Veillonella parvula
Veillonella sp.
Victivallis vadensis
candidate division

Supragingival Plaque

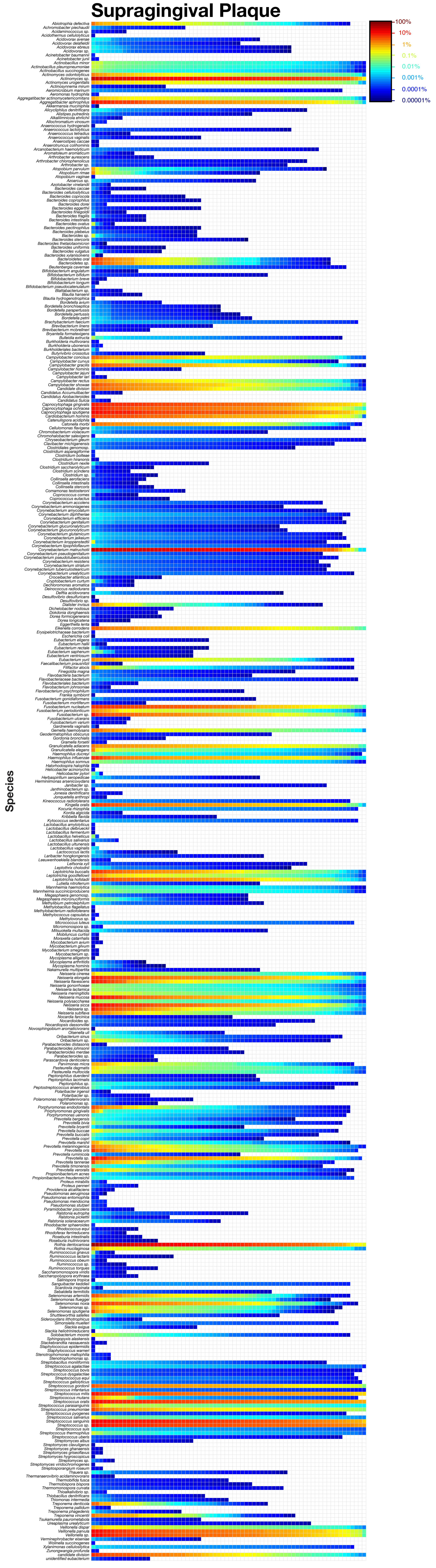

Tongue Dorsum

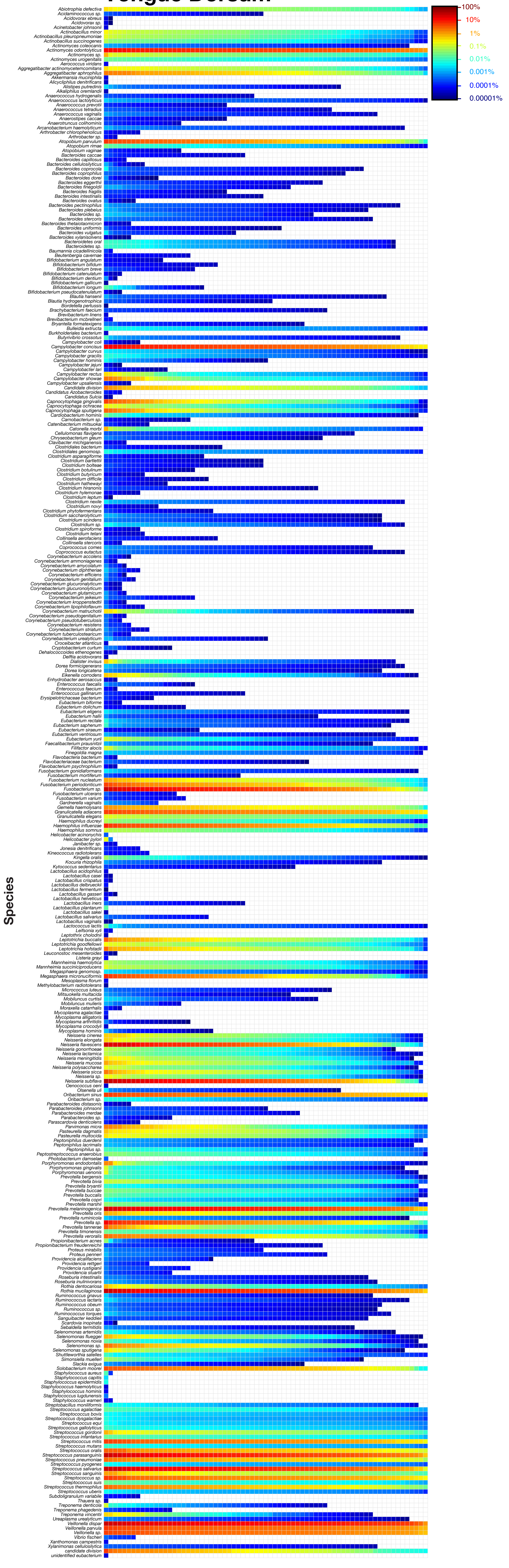

Supplement: Figure S5 — Heat maps of ordered species abundance. Heat map representation of the relative abundance of each species ordered by the relative abundance for each species (for each sample the abundances of strains within a species were summed to calculate the species total). Species are listed alphabetically. (PDF) [file pone.0097279.s005.pdf]
