# Supplementary material for: The Prevalence of Species and Strains in the Human Microbiome: A Resource for Experimental Efforts
Source: PLoS One. 2014 May 14;9(5):e97279. doi: 10.1371/journal.pone.0097279 (PMC4020798; doi:10.1371/journal.pone.0097279)
Supplement: Figure S7 — 16 S heat maps and comparison with WGS data. (A–G) These figures show a heat map representation of the relative abundance of each genus (y-axis) for each sample (x-axis) for six body sites, both individually (A–F) and together (G), as determined by 16 S sequence data. The abundances are hierarchically clustered using Spearman rank correlation with average linkage. (H–L) For each genus that was represented by 3 or more species in the whole genome shotgun data, the rank order for each sample was determined and compared to the average rank order of the genus using Kendall's W. The correlations are hierarchically clustered using Spearman rank correlation with average linkage. The overall concordance of all the samples is shown in the far right column. Genera with a high, statistically significant Kendall's W (>0.8) are colored red. (PDF) [file pone.0097279.s007.pdf]

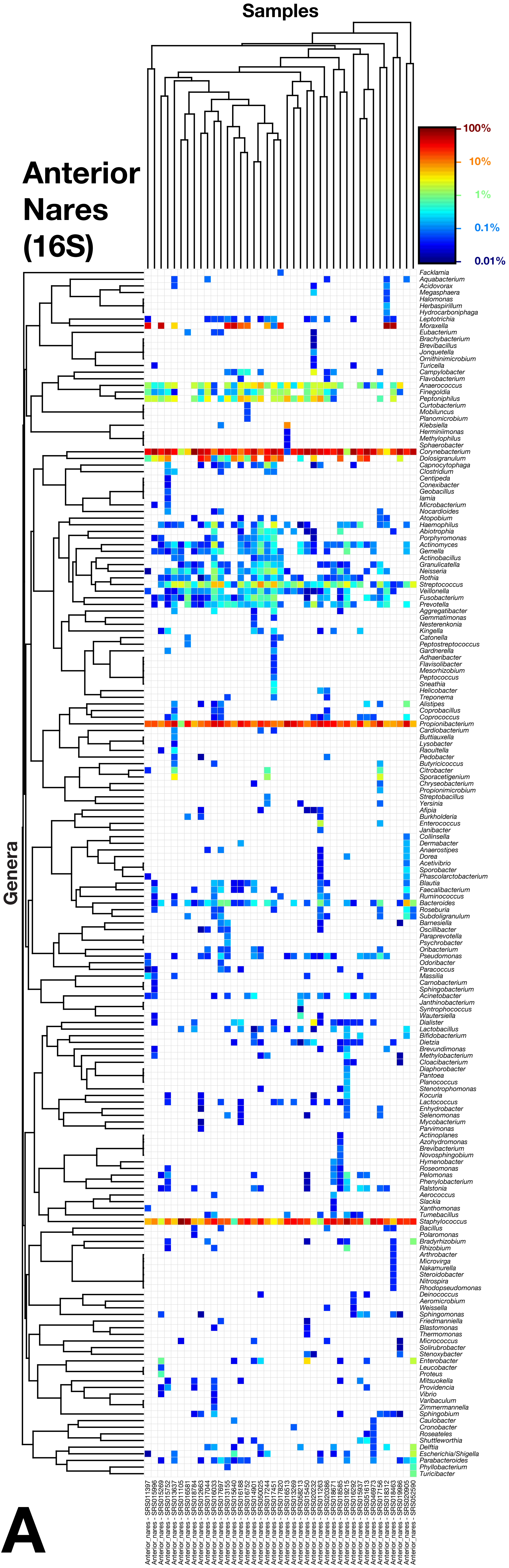

Samples
Anterior Nares (16S)
100%
10%
1%
0.1%
0.01%
Genera
Facklamia
Aquabacterium
Acidovorax
Megasphaera
Halomonas
Herbaspirillum
Hydrocarboniphaga
Leptotrichia
Moraxella
Eubacterium
Brachybacterium
Brevibacillus
Jonquetella
Ornithinimicrobium
Turicella
Campylobacter
Flavobacterium
Anaerococcus
Finegoldia
Peptoniphilus
Curtobacterium
Mobiluncus
Planomicrobium
Klebsiella
Herminiimonas
Methylophilus
Sphaerobacter
Corynebacterium
Dolosigranulum
Capnocytophaga
Clostridium
Centipeda
Conexibacter
Geobacillus
Iamia
Microbacterium
Nocardioides
Atopobium
Haemophilus
Abiotrophia
Porphyromonas
Actinomyces
Gemella
Actinobacillus
Granulicatella
Neisseria
Rothia
Streptococcus
Veillonella
Fusobacterium
Prevotella
Aggregatibacter
Gemmatimonas
Nesterenkonia
Kingella
Catonella
Peptostreptococcus
Gardnerella
Adhaeribacter
Flavisolibacter
Mesorhizobium
Peptococcus
Sneathia
Helicobacter
Treponema
Alistipes
Coprobacillus
Coprococcus
Propionibacterium
Cardiobacterium
Buttiauxella
Lysobacter
Raoultella
Pedobacter
Butyricicoccus
Citrobacter
Sporacetigenium
Chryseobacterium
Propionimicrobium
Streptobacillus
Yersinia
Afipia
Burkholderia
Enterococcus
Janibacter
Collinsella
Dermabacter
Anaerostipes
Dorea
Acetivibrio
Sporobacter
Phascolarctobacterium
Blautia
Faecalibacterium
Ruminococcus
Bacteroides
Roseburia
Subdoligranulum
Barnesiella
Oscillibacter
Paraprevotella
Psychrobacter
Oribacterium
Pseudomonas
Odoribacter
Paracoccus
Massilia
Carnobacterium
Sphingobacterium
Acinetobacter
Janthinobacterium
Syntrophococcus
Wautersiella
Dialister
Lactobacillus
Bifidobacterium
Dietzia
Brevundimonas
Methylobacterium
Cloacibacterium
Diaphorobacter
Pantoea
Planococcus
Stenotrophomonas
Kocuria
Lactococcus
Enhydrobacter
Selenomonas
Mycobacterium
Parvimonas
Actinoplanes
Azohydromonas
Brevibacterium
Novosphingobium
Hymenobacter
Roseomonas
Pelomonas
Phenylobacterium
Ralstonia
Aerococcus
Slackia
Xanthomonas
Tumebacillus
Staphylococcus
Bacillus
Polaromonas
Bradyrhizobium
Rhizobium
Arthrobacter
Microvirga
Nakamurella
Steroidobacter
Nitrospira
Rhodopseudomonas
Deinococcus
Aeromicrobium
Weissella
Sphingomonas
Friedmanniella
Blastomonas
Thermomonas
Micrococcus
Solirubrobacter
Stenoxybacter
Enterobacter
Leucobacter
Proteus
Mitsuokella
Providencia
Vibrio
Varibaculum
Zimmermannella
Sphingobium
Caulobacter
Cronobacter
Roseateles
Shuttleworthia
Delftia
Escherichia/Shigella
Parabacteroides
Phyllobacterium
Turicibacter
Anterior_nares - SRS011397
Anterior_nares - SRS015996
Anterior_nares - SRS015269
Anterior_nares - SRS015752
Anterior_nares - SRS013637
Anterior_nares - SRS011105
Anterior_nares - SRS016581
Anterior_nares - SRS018784
Anterior_nares - SRS012663
Anterior_nares - SRS017044
Anterior_nares - SRS016033
Anterior_nares - SRS017697
Anterior_nares - SRS013155
Anterior_nares - SRS015640
Anterior_nares - SRS016188
Anterior_nares - SRS016752
Anterior_nares - SRS014901
Anterior_nares - SRS050025
Anterior_nares - SRS017244
Anterior_nares - SRS017451
Anterior_nares - SRS017820
Anterior_nares - SRS016513
Anterior_nares - SRS013269
Anterior_nares - SRS058213
Anterior_nares - SRS015450
Anterior_nares - SRS020232
Anterior_nares - SRS011263
Anterior_nares - SRS020386
Anterior_nares - SRS018671
Anterior_nares - SRS018585
Anterior_nares - SRS019215
Anterior_nares - SRS016292
Anterior_nares - SRS015937
Anterior_nares - SRS051613
Anterior_nares - SRS046973
Anterior_nares - SRS017156
Anterior_nares - SRS018312
Anterior_nares - SRS018463
Anterior_nares - SRS019986
Anterior_nares - SRS020905
Anterior_nares - SRS052590

A

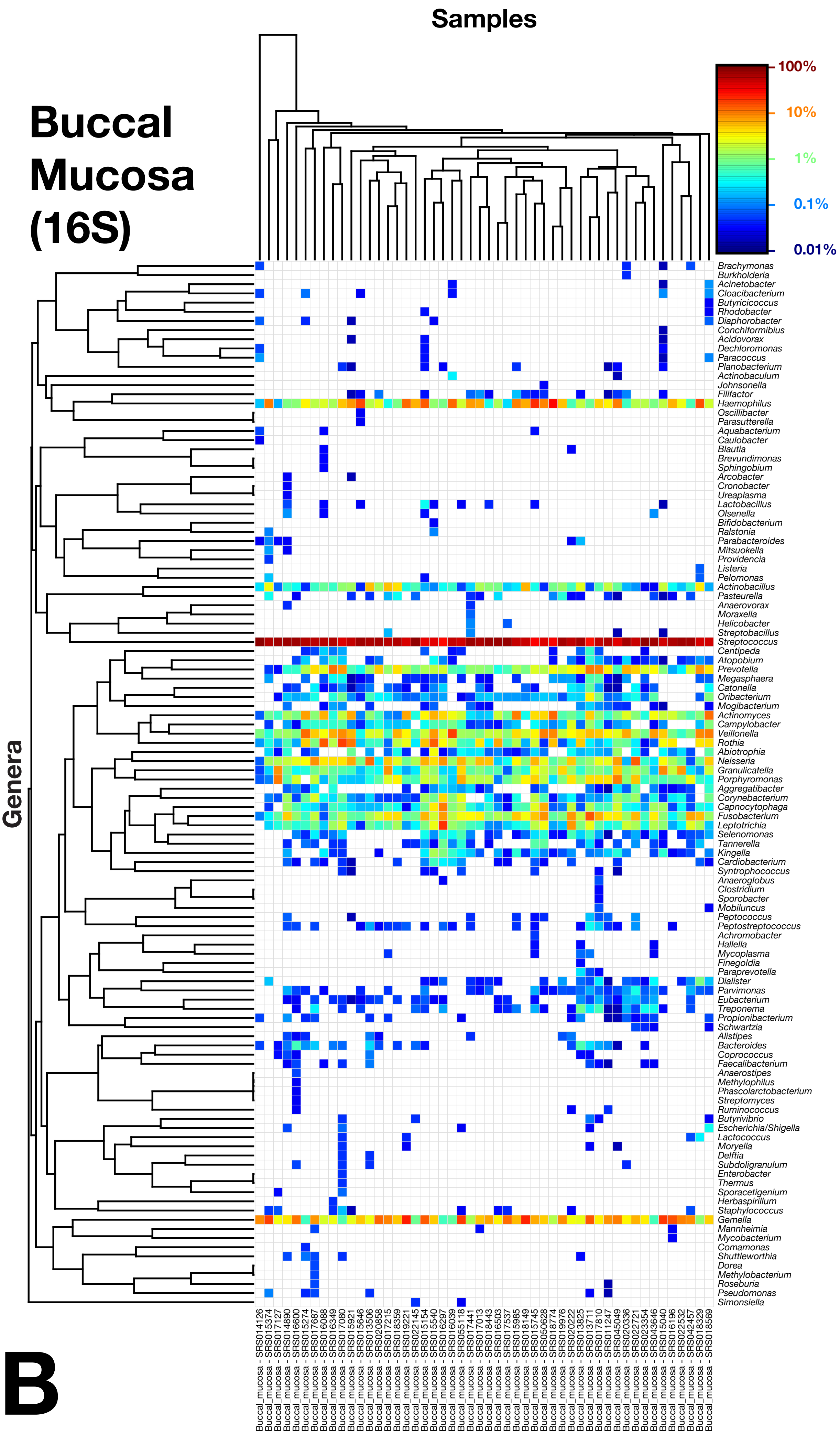
Samples
Buccal
Mucosa
(16S)
100%
10%
1%
0.1%
0.01%
Genera
Brachymonas
Burkholderia
Acinetobacter
Cloacibacterium
Butyricicoccus
Rhodobacter
Diaphorobacter
Conchiformibius
Acidovorax
Dechloromonas
Paracoccus
Planobacterium
Actinobaculum
Johnsonella
Filifactor
Haemophilus
Oscillibacter
Parasutterella
Aquabacterium
Caulobacter
Blautia
Brevundimonas
Sphingobium
Arcobacter
Cronobacter
Ureaplasma
Lactobacillus
Olsenella
Bifidobacterium
Ralstonia
Parabacteroides
Mitsuokella
Providencia
Listeria
Pelomonas
Actinobacillus
Pasteurella
Anaerovorax
Moraxella
Helicobacter
Streptobacillus
Streptococcus
Centipeda
Atopobium
Prevotella
Megasphaera
Catonella
Oribacterium
Mogibacterium
Actinomyces
Campylobacter
Veillonella
Rothia
Abiotrophia
Neisseria
Granulicatella
Porphyromonas
Aggregatibacter
Corynebacterium
Capnocytophaga
Fusobacterium
Leptotrichia
Selenomonas
Tannerella
Kingella
Cardiobacterium
Syntrophococcus
Anaeroglobus
Clostridium
Sporobacter
Mobiluncus
Peptococcus
Peptostreptococcus
Achromobacter
Hallella
Mycoplasma
Finegoldia
Paraprevotella
Dialister
Parvimonas
Eubacterium
Treponema
Propionibacterium
Schwartzia
Alistipes
Bacteroides
Coprococcus
Faecalibacterium
Anaerostipes
Methylophilus
Phascolarctobacterium
Streptomyces
Ruminococcus
Butyrivibrio
Escherichia/Shigella
Lactococcus
Moryella
Delftia
Subdoligranulum
Enterobacter
Thermus
Sporacetigenium
Herbaspirillum
Staphylococcus
Gemella
Mannheimia
Mycobacterium
Comamonas
Shuttleworthia
Dorea
Methylobacterium
Roseburia
Pseudomonas
Simonsiella
Buccal_mucosa - SRS014126
Buccal_mucosa - SRS015374
Buccal_mucosa - SRS017127
Buccal_mucosa - SRS014890
Buccal_mucosa - SRS016600
Buccal_mucosa - SRS015274
Buccal_mucosa - SRS017687
Buccal_mucosa - SRS016088
Buccal_mucosa - SRS016349
Buccal_mucosa - SRS017080
Buccal_mucosa - SRS015921
Buccal_mucosa - SRS015646
Buccal_mucosa - SRS013506
Buccal_mucosa - SRS020858
Buccal_mucosa - SRS017215
Buccal_mucosa - SRS018359
Buccal_mucosa - SRS019221
Buccal_mucosa - SRS022145
Buccal_mucosa - SRS015154
Buccal_mucosa - SRS015540
Buccal_mucosa - SRS016297
Buccal_mucosa - SRS016039
Buccal_mucosa - SRS055118
Buccal_mucosa - SRS017441
Buccal_mucosa - SRS017013
Buccal_mucosa - SRS018443
Buccal_mucosa - SRS016503
Buccal_mucosa - SRS017537
Buccal_mucosa - SRS015985
Buccal_mucosa - SRS018149
Buccal_mucosa - SRS015745
Buccal_mucosa - SRS050628
Buccal_mucosa - SRS018774
Buccal_mucosa - SRS019976
Buccal_mucosa - SRS020222
Buccal_mucosa - SRS013825
Buccal_mucosa - SRS013711
Buccal_mucosa - SRS017810
Buccal_mucosa - SRS011247
Buccal_mucosa - SRS045049
Buccal_mucosa - SRS020336
Buccal_mucosa - SRS022721
Buccal_mucosa - SRS023354
Buccal_mucosa - SRS043646
Buccal_mucosa - SRS015040
Buccal_mucosa - SRS016196
Buccal_mucosa - SRS022532
Buccal_mucosa - SRS042457
Buccal_mucosa - SRS018329
Buccal_mucosa - SRS018569
B

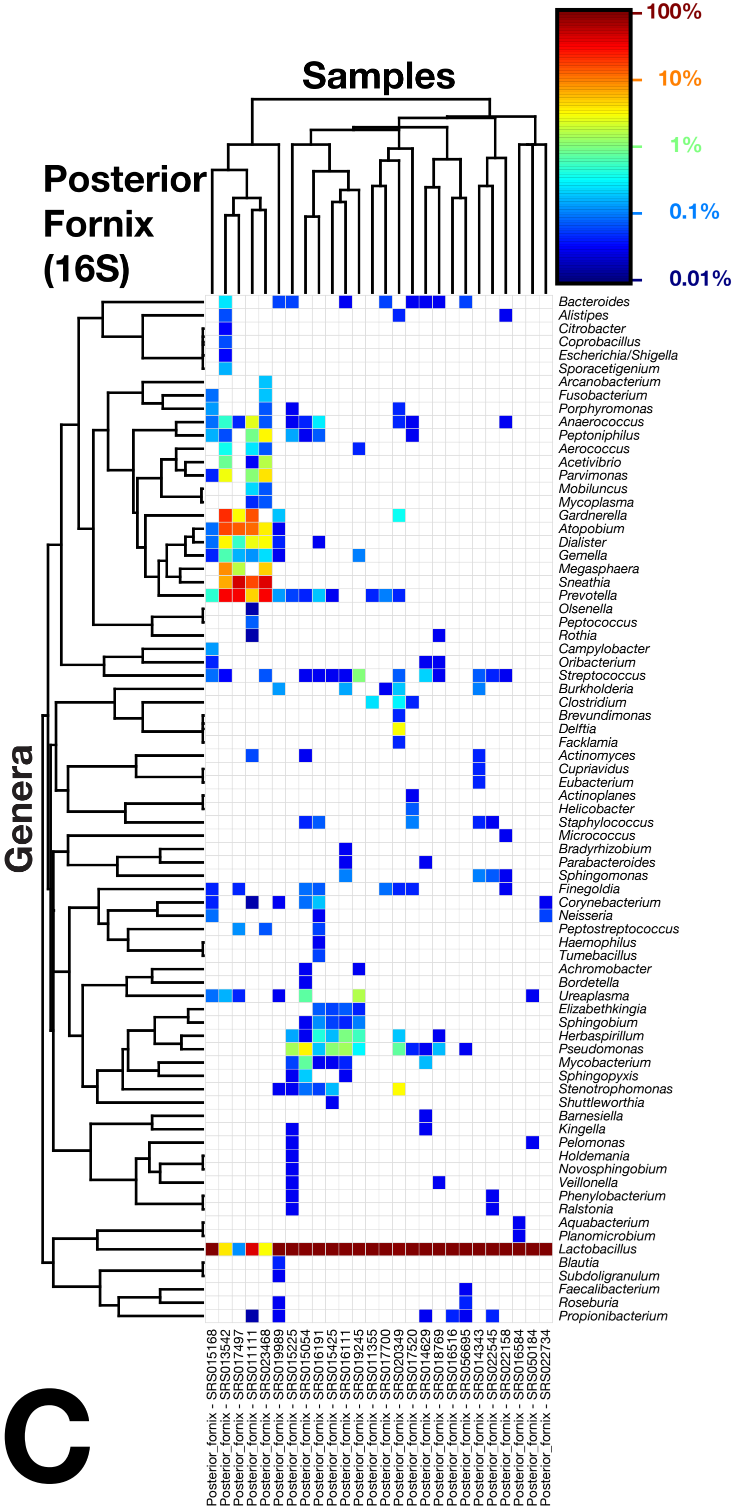
Posterior Fornix (16S)
Samples
Genera
100%
10%
1%
0.1%
0.01%
Bacteroides
Alistipes
Citrobacter
Coprobacillus
Escherichia/Shigella
Sporacetigenium
Arcanobacterium
Fusobacterium
Porphyromonas
Anaerococcus
Peptoniphilus
Aerococcus
Acetivibrio
Parvimonas
Mobiluncus
Mycoplasma
Gardnerella
Atopobium
Dialister
Gemella
Megasphaera
Sneathia
Prevotella
Olsenella
Peptococcus
Rothia
Campylobacter
Oribacterium
Streptococcus
Burkholderia
Clostridium
Brevundimonas
Delftia
Facklamia
Actinomyces
Cupriavidus
Eubacterium
Actinoplanes
Helicobacter
Staphylococcus
Micrococcus
Bradyrhizobium
Parabacteroides
Sphingomonas
Finegoldia
Corynebacterium
Neisseria
Peptostreptococcus
Haemophilus
Tumebacillus
Achromobacter
Bordetella
Ureaplasma
Elizabethkingia
Sphingobium
Herbaspirillum
Pseudomonas
Mycobacterium
Sphingopyxis
Stenotrophomonas
Shuttleworthia
Barnesiella
Kingella
Pelomonas
Holdemania
Novosphingobium
Veillonella
Phenylobacterium
Ralstonia
Aquabacterium
Planomicrobium
Lactobacillus
Blautia
Subdoligranulum
Faecalibacterium
Roseburia
Propionibacterium
Posterior_fornix - SRS015168
Posterior_fornix - SRS013542
Posterior_fornix - SRS017497
Posterior_fornix - SRS011111
Posterior_fornix - SRS023468
Posterior_fornix - SRS019989
Posterior_fornix - SRS015225
Posterior_fornix - SRS015054
Posterior_fornix - SRS016191
Posterior_fornix - SRS015425
Posterior_fornix - SRS016111
Posterior_fornix - SRS019245
Posterior_fornix - SRS011355
Posterior_fornix - SRS017700
Posterior_fornix - SRS020349
Posterior_fornix - SRS017520
Posterior_fornix - SRS014629
Posterior_fornix - SRS018769
Posterior_fornix - SRS016516
Posterior_fornix - SRS056695
Posterior_fornix - SRS014343
Posterior_fornix - SRS022545
Posterior_fornix - SRS022158
Posterior_fornix - SRS016584
Posterior_fornix - SRS050184
Posterior_fornix - SRS022734
C

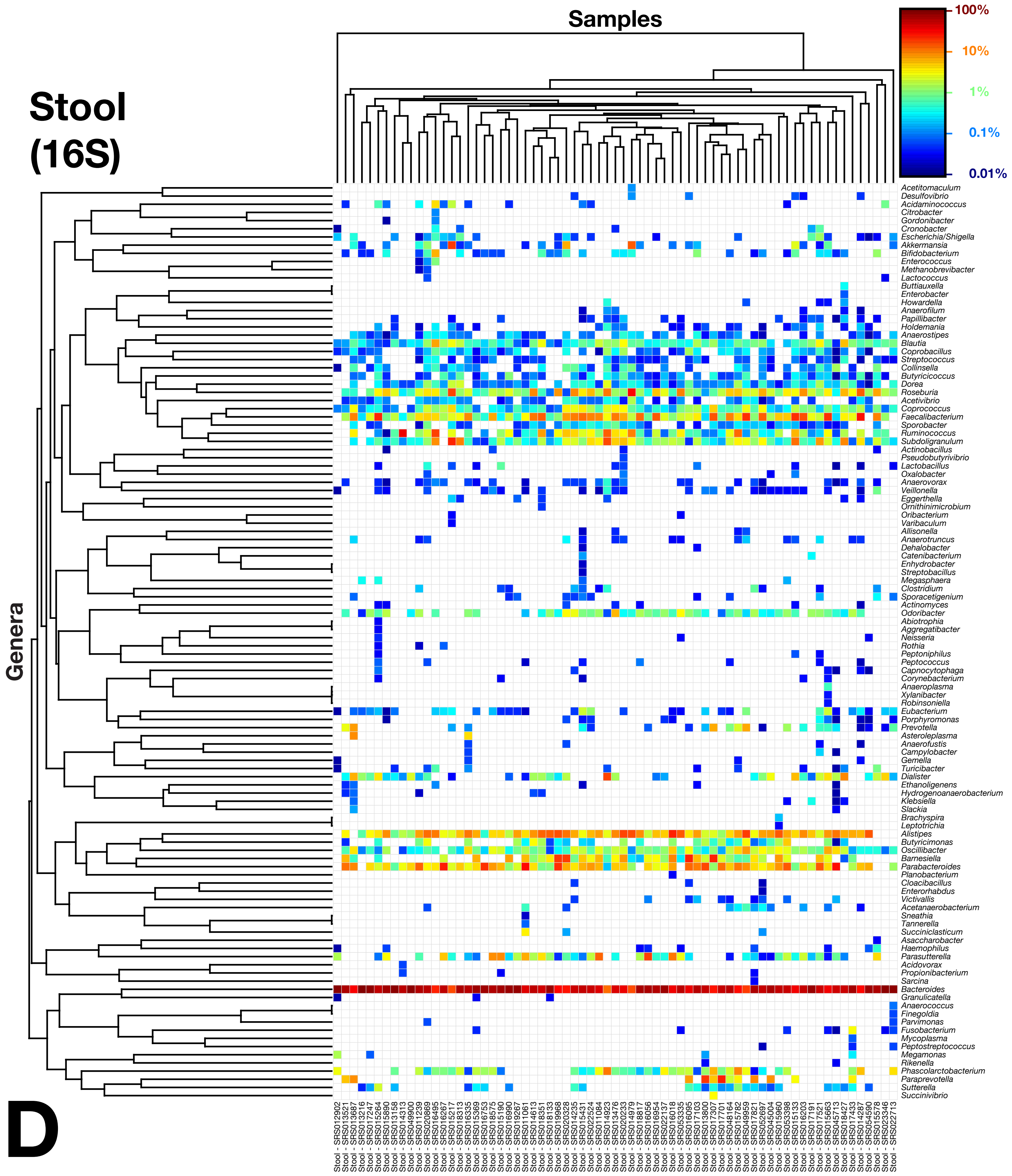
Stool
(16S)
Samples
100%
10%
1%
0.1%
0.01%
Genera
Acetitomaculum
Desulfovibrio
Acidaminococcus
Citrobacter
Gordonibacter
Cronobacter
Escherichia/Shigella
Akkermansia
Bifidobacterium
Enterococcus
Methanobrevibacter
Lactococcus
Buttiauxella
Enterobacter
Howardella
Anaerofilum
Papillibacter
Holdemania
Anaerostipes
Blautia
Coprobacillus
Streptococcus
Collinsella
Butyricicoccus
Dorea
Roseburia
Acetivibrio
Coprococcus
Faecalibacterium
Sporobacter
Ruminococcus
Subdoligranulum
Actinobacillus
Pseudobutyrivibrio
Lactobacillus
Oxalobacter
Anaerovorax
Veillonella
Eggerthella
Ornithinimicrobium
Oribacterium
Varibaculum
Allisonella
Anaerotruncus
Dehalobacter
Catenibacterium
Enhydrobacter
Streptobacillus
Megasphaera
Clostridium
Sporacetigenium
Actinomyces
Odoribacter
Abiotrophia
Aggregatibacter
Neisseria
Rothia
Peptoniphilus
Peptococcus
Capnocytophaga
Corynebacterium
Anaeroplasma
Xylanibacter
Robinsoniella
Eubacterium
Porphyromonas
Prevotella
Asteroleplasma
Anaerofustis
Campylobacter
Gemella
Turicibacter
Dialister
Ethanoligenens
Hydrogenoanaerobacterium
Klebsiella
Slackia
Brachyspira
Leptotrichia
Alistipes
Butyricimonas
Oscillibacter
Barnesiella
Parabacteroides
Planobacterium
Cloacibacillus
Enterorhabdus
Victivallis
Acetanaerobacterium
Sneathia
Tannerella
Succiniclasticum
Asaccharobacter
Haemophilus
Parasutterella
Acidovorax
Propionibacterium
Sarcina
Bacteroides
Granulicatella
Anaerococcus
Finegoldia
Parvimonas
Fusobacterium
Mycoplasma
Peptostreptococcus
Megamonas
Rikenella
Phascolarctobacterium
Paraprevotella
Sutterella
Succinivibrio
D

E

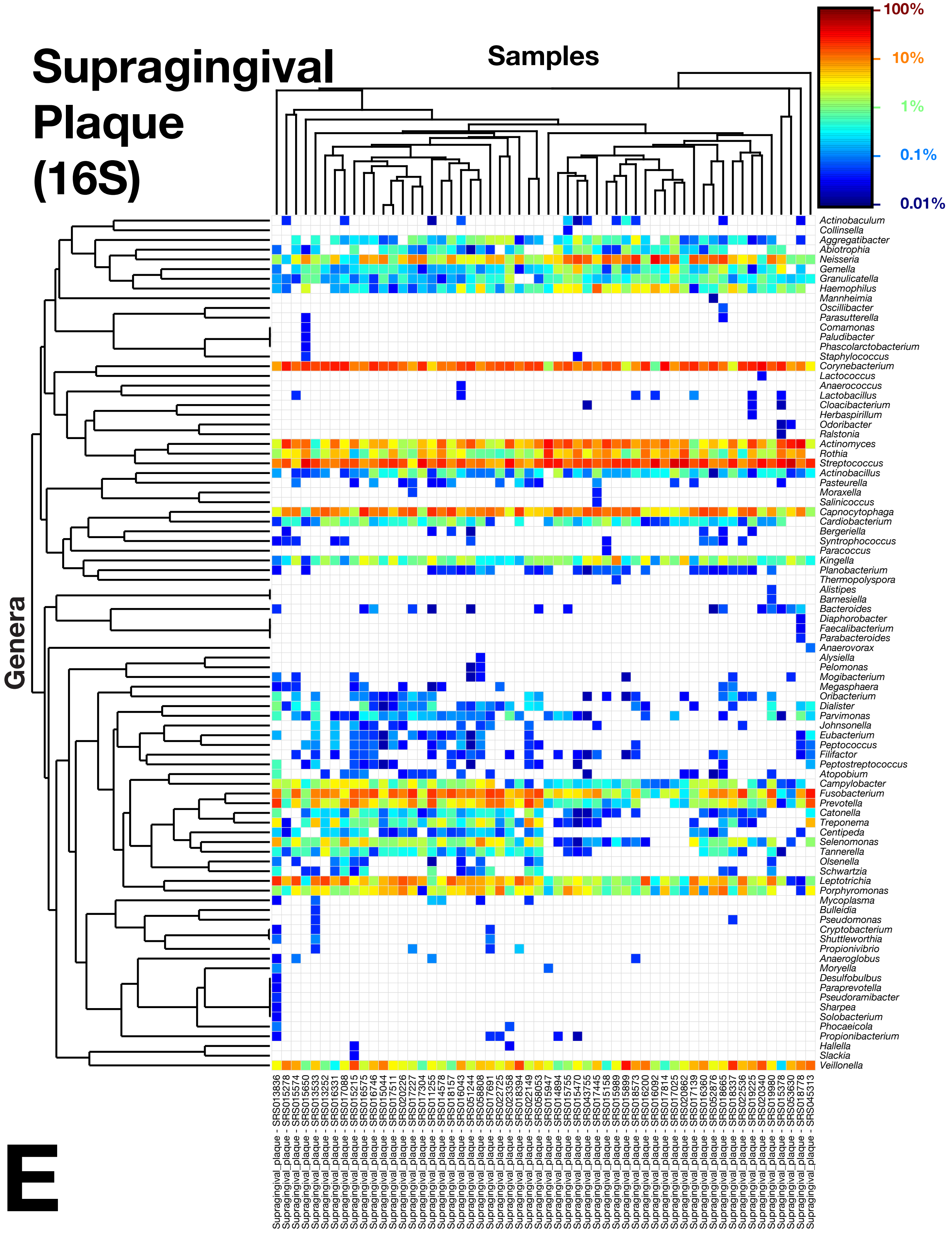
Supragingival Plaque (16S)
Samples
Genera
100%
10%
1%
0.1%
0.01%
Actinobaculum
Collinsella
Aggregatibacter
Abiotrophia
Neisseria
Gemella
Granulicatella
Haemophilus
Mannheimia
Oscillibacter
Parasutterella
Comamonas
Paludibacter
Phascolarctobacterium
Staphylococcus
Corynebacterium
Lactococcus
Anaerococcus
Lactobacillus
Cloacibacterium
Herbaspirillum
Odoribacter
Ralstonia
Actinomyces
Rothia
Streptococcus
Actinobacillus
Pasteurella
Moraxella
Salinicoccus
Capnocytophaga
Cardiobacterium
Bergeriella
Syntrophococcus
Paracoccus
Kingella
Planobacterium
Thermopolyspora
Alistipes
Barnesiella
Bacteroides
Diaphorobacter
Faecalibacterium
Parabacteroides
Anaerovorax
Alysiella
Pelomonas
Mogibacterium
Megasphaera
Oribacterium
Dialister
Parvimonas
Johnsonella
Eubacterium
Peptococcus
Filifactor
Peptostreptococcus
Atopobium
Campylobacter
Fusobacterium
Prevotella
Catonella
Treponema
Centipeda
Selenomonas
Tannerella
Olsenella
Schwartzia
Leptotrichia
Porphyromonas
Mycoplasma
Bulleidia
Pseudomonas
Cryptobacterium
Shuttleworthia
Propionivibrio
Anaeroglobus
Moryella
Desulfobulbus
Paraprevotella
Pseudoramibacter
Sharpea
Solobacterium
Phocaeicola
Propionibacterium
Hallella
Slackia
Veillonella
Supragingival_plaque - SRS013836
Supragingival_plaque - SRS015278
Supragingival_plaque - SRS015574
Supragingival_plaque - SRS015650
Supragingival_plaque - SRS013533
Supragingival_plaque - SRS013252
Supragingival_plaque - SRS016331
Supragingival_plaque - SRS017088
Supragingival_plaque - SRS015215
Supragingival_plaque - SRS016575
Supragingival_plaque - SRS016746
Supragingival_plaque - SRS015044
Supragingival_plaque - SRS017511
Supragingival_plaque - SRS020226
Supragingival_plaque - SRS017227
Supragingival_plaque - SRS017304
Supragingival_plaque - SRS011255
Supragingival_plaque - SRS014578
Supragingival_plaque - SRS018157
Supragingival_plaque - SRS016043
Supragingival_plaque - SRS051244
Supragingival_plaque - SRS058808
Supragingival_plaque - SRS017691
Supragingival_plaque - SRS022725
Supragingival_plaque - SRS023358
Supragingival_plaque - SRS018394
Supragingival_plaque - SRS022149
Supragingival_plaque - SRS058053
Supragingival_plaque - SRS015947
Supragingival_plaque - SRS014894
Supragingival_plaque - SRS015755
Supragingival_plaque - SRS015470
Supragingival_plaque - SRS043755
Supragingival_plaque - SRS017445
Supragingival_plaque - SRS015158
Supragingival_plaque - SRS015989
Supragingival_plaque - SRS015899
Supragingival_plaque - SRS018573
Supragingival_plaque - SRS016200
Supragingival_plaque - SRS016092
Supragingival_plaque - SRS017814
Supragingival_plaque - SRS017025
Supragingival_plaque - SRS020862
Supragingival_plaque - SRS017139
Supragingival_plaque - SRS016360
Supragingival_plaque - SRS052876
Supragingival_plaque - SRS018665
Supragingival_plaque - SRS018337
Supragingival_plaque - SRS022536
Supragingival_plaque - SRS019225
Supragingival_plaque - SRS020340
Supragingival_plaque - SRS019980
Supragingival_plaque - SRS015378
Supragingival_plaque - SRS053630
Supragingival_plaque - SRS018778
Supragingival_plaque - SRS045313

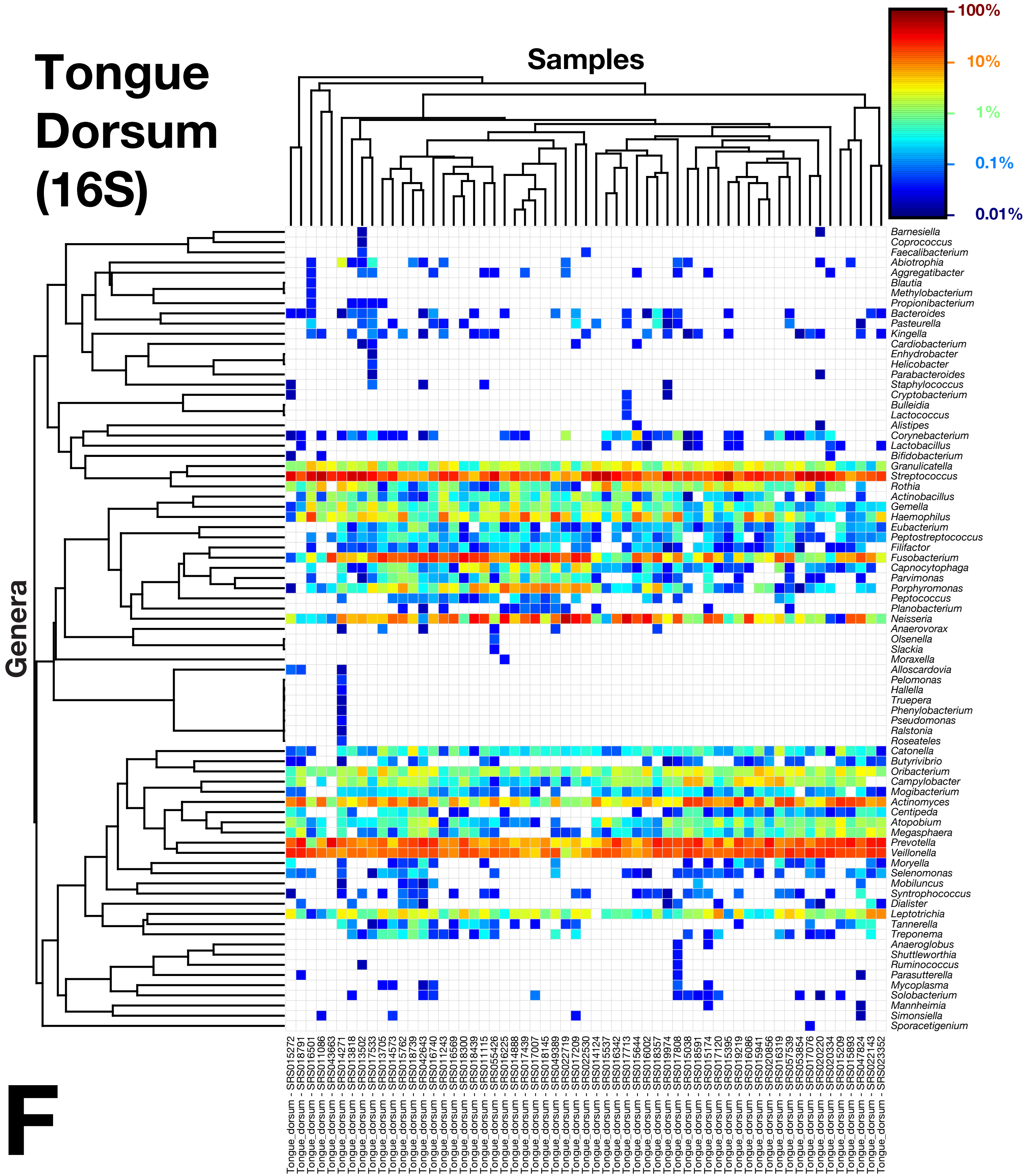
Tongue
Dorsum
(16S)
Samples
Genera
100%
10%
1%
0.1%
0.01%
Barnesiella
Coprococcus
Faecalibacterium
Abiotrophia
Aggregatibacter
Blautia
Methylobacterium
Propionibacterium
Bacteroides
Pasteurella
Kingella
Cardiobacterium
Enhydrobacter
Helicobacter
Parabacteroides
Staphylococcus
Cryptobacterium
Bulleidia
Lactococcus
Alistipes
Corynebacterium
Lactobacillus
Bifidobacterium
Granulicatella
Streptococcus
Rothia
Actinobacillus
Gemella
Haemophilus
Eubacterium
Peptostreptococcus
Filifactor
Fusobacterium
Capnocytophaga
Parvimonas
Porphyromonas
Peptococcus
Planobacterium
Neisseria
Anaerovorax
Olsenella
Slackia
Moraxella
Alloscardovia
Pelomonas
Hallella
Truepera
Phenylobacterium
Pseudomonas
Ralstonia
Roseateles
Catonella
Butyrivibrio
Oribacterium
Campylobacter
Mogibacterium
Actinomyces
Centipeda
Atopobium
Megasphaera
Prevotella
Veillonella
Moryella
Selenomonas
Mobiluncus
Syntrophococcus
Dialister
Leptotrichia
Tannerella
Treponema
Anaeroglobus
Shuttleworthia
Ruminococcus
Parasutterella
Mycoplasma
Solobacterium
Mannheimia
Simonsiella
Sporacetigenium
Tongue_dorsum - SRS015272
Tongue_dorsum - SRS018791
Tongue_dorsum - SRS016501
Tongue_dorsum - SRS011086
Tongue_dorsum - SRS043663
Tongue_dorsum - SRS014271
Tongue_dorsum - SRS013818
Tongue_dorsum - SRS013502
Tongue_dorsum - SRS017533
Tongue_dorsum - SRS013705
Tongue_dorsum - SRS014573
Tongue_dorsum - SRS015762
Tongue_dorsum - SRS018739
Tongue_dorsum - SRS042643
Tongue_dorsum - SRS016740
Tongue_dorsum - SRS011243
Tongue_dorsum - SRS016569
Tongue_dorsum - SRS018300
Tongue_dorsum - SRS018439
Tongue_dorsum - SRS011115
Tongue_dorsum - SRS055426
Tongue_dorsum - SRS016225
Tongue_dorsum - SRS014888
Tongue_dorsum - SRS017439
Tongue_dorsum - SRS017007
Tongue_dorsum - SRS018145
Tongue_dorsum - SRS049389
Tongue_dorsum - SRS022719
Tongue_dorsum - SRS017209
Tongue_dorsum - SRS022530
Tongue_dorsum - SRS014124
Tongue_dorsum - SRS015537
Tongue_dorsum - SRS016342
Tongue_dorsum - SRS017713
Tongue_dorsum - SRS015644
Tongue_dorsum - SRS016002
Tongue_dorsum - SRS018357
Tongue_dorsum - SRS019974
Tongue_dorsum - SRS017808
Tongue_dorsum - SRS015038
Tongue_dorsum - SRS018591
Tongue_dorsum - SRS015174
Tongue_dorsum - SRS017120
Tongue_dorsum - SRS015395
Tongue_dorsum - SRS019219
Tongue_dorsum - SRS016086
Tongue_dorsum - SRS015941
Tongue_dorsum - SRS020856
Tongue_dorsum - SRS016319
Tongue_dorsum - SRS057539
Tongue_dorsum - SRS053854
Tongue_dorsum - SRS017076
Tongue_dorsum - SRS020220
Tongue_dorsum - SRS020334
Tongue_dorsum - SRS015209
Tongue_dorsum - SRS015893
Tongue_dorsum - SRS047824
Tongue_dorsum - SRS022143
Tongue_dorsum - SRS023352
F

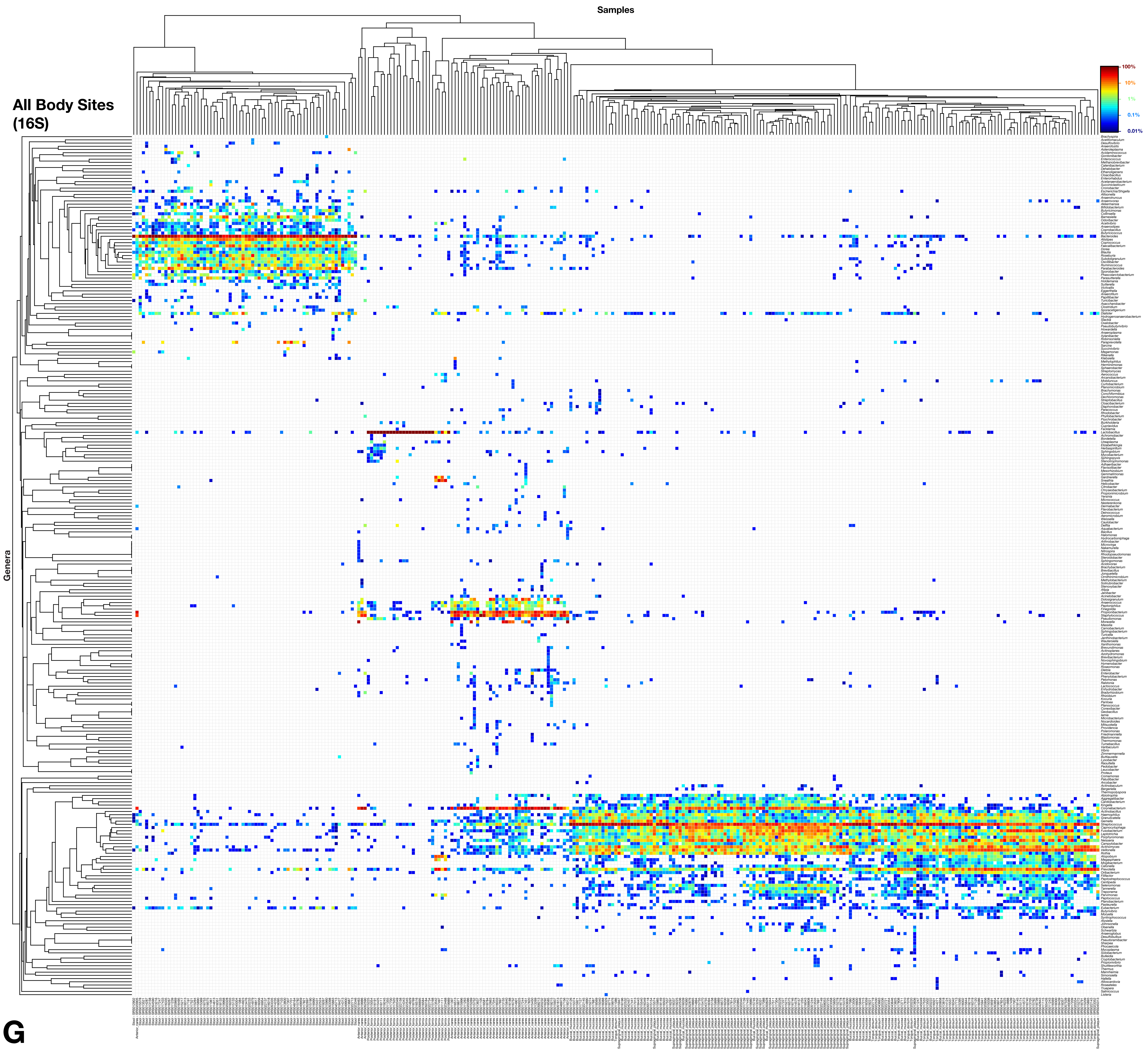

G

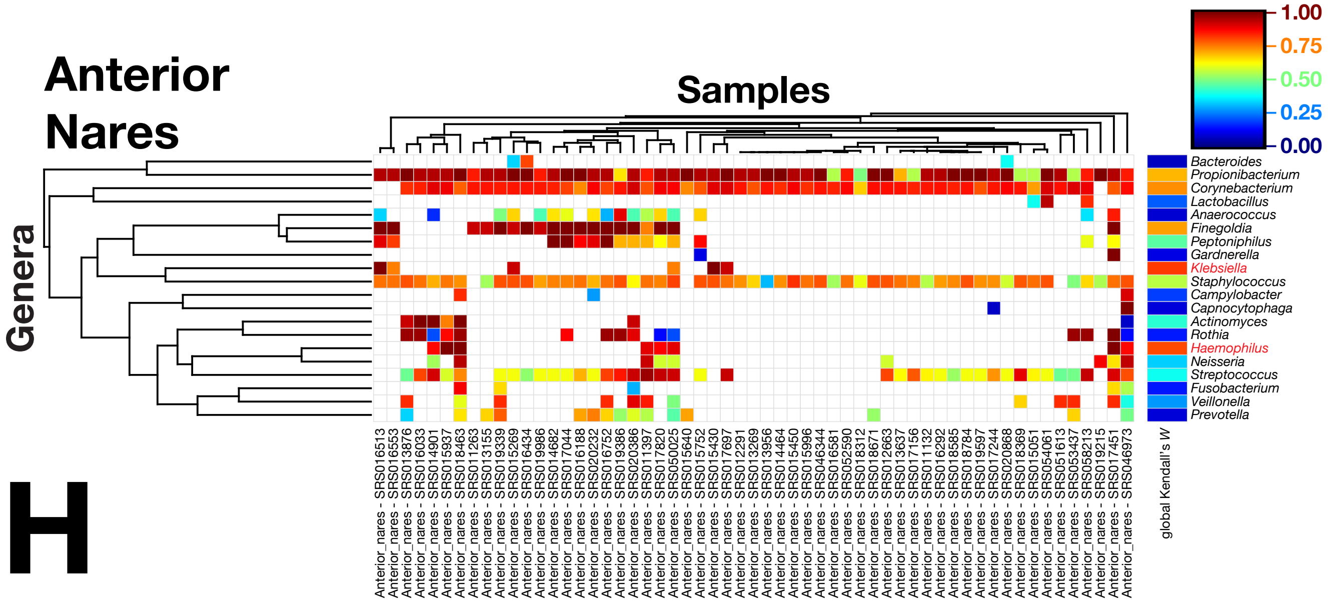
Anterior Nares
Samples
Genera
H
1.00
0.75
0.50
0.25
0.00
Bacteroides
Propionibacterium
Corynebacterium
Lactobacillus
Anaerococcus
Finegoldia
Peptoniphilus
Gardnerella
Klebsiella
Staphylococcus
Campylobacter
Capnocytophaga
Actinomyces
Rothia
Haemophilus
Neisseria
Streptococcus
Fusobacterium
Veillonella
Prevotella
global Kendall's W
Anterior_nares - SRS016513
Anterior_nares - SRS016553
Anterior_nares - SRS013876
Anterior_nares - SRS016033
Anterior_nares - SRS014901
Anterior_nares - SRS015937
Anterior_nares - SRS018463
Anterior_nares - SRS011263
Anterior_nares - SRS013155
Anterior_nares - SRS019339
Anterior_nares - SRS015269
Anterior_nares - SRS016434
Anterior_nares - SRS019986
Anterior_nares - SRS014682
Anterior_nares - SRS017044
Anterior_nares - SRS016188
Anterior_nares - SRS020232
Anterior_nares - SRS016752
Anterior_nares - SRS019386
Anterior_nares - SRS020386
Anterior_nares - SRS011397
Anterior_nares - SRS017820
Anterior_nares - SRS050025
Anterior_nares - SRS015640
Anterior_nares - SRS015752
Anterior_nares - SRS015430
Anterior_nares - SRS017697
Anterior_nares - SRS012291
Anterior_nares - SRS013269
Anterior_nares - SRS013956
Anterior_nares - SRS014464
Anterior_nares - SRS015450
Anterior_nares - SRS015996
Anterior_nares - SRS046344
Anterior_nares - SRS016581
Anterior_nares - SRS052590
Anterior_nares - SRS018312
Anterior_nares - SRS018671
Anterior_nares - SRS012663
Anterior_nares - SRS013637
Anterior_nares - SRS017156
Anterior_nares - SRS011132
Anterior_nares - SRS016292
Anterior_nares - SRS018585
Anterior_nares - SRS018784
Anterior_nares - SRS019597
Anterior_nares - SRS017244
Anterior_nares - SRS020868
Anterior_nares - SRS018369
Anterior_nares - SRS015051
Anterior_nares - SRS054061
Anterior_nares - SRS051613
Anterior_nares - SRS053437
Anterior_nares - SRS058213
Anterior_nares - SRS019215
Anterior_nares - SRS017451
Anterior_nares - SRS046973

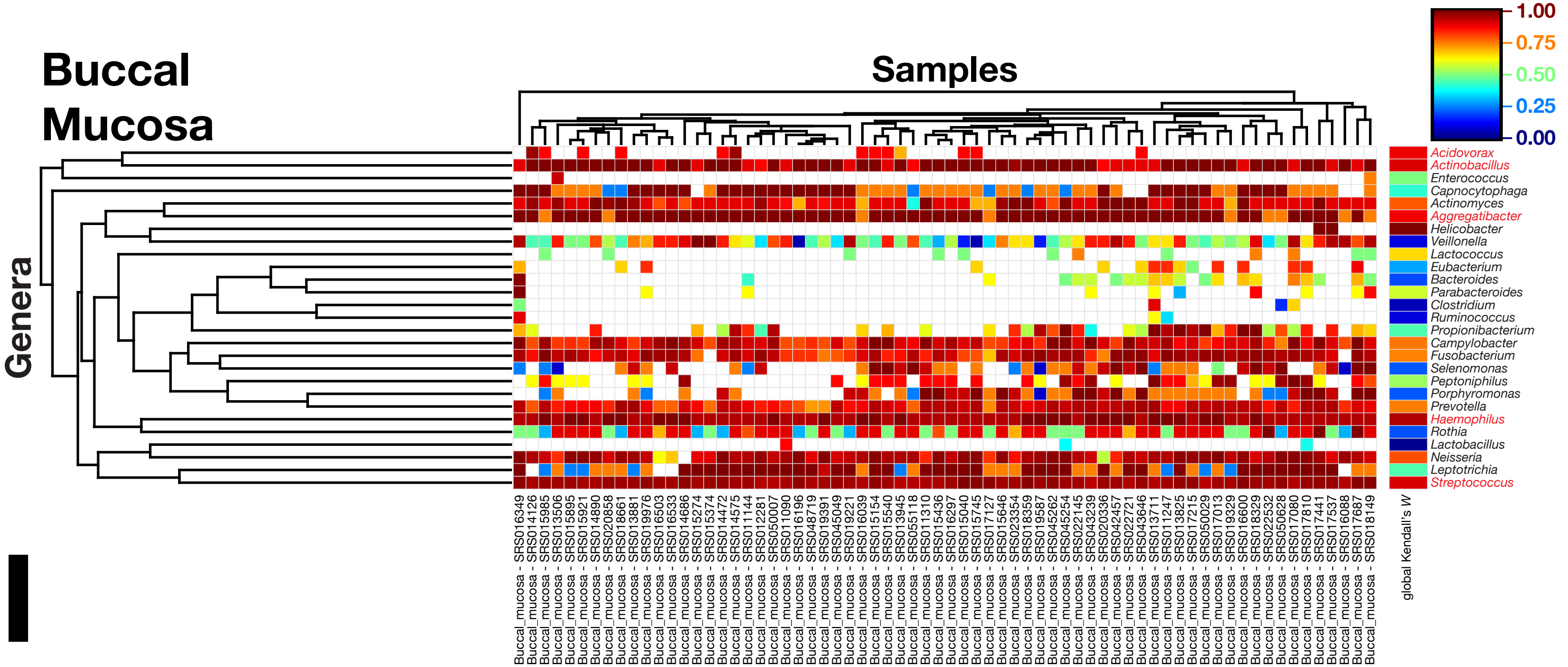

Buccal Mucosa
Samples
Genera
1.00
0.75
0.50
0.25
0.00
Acidovorax
Actinobacillus
Enterococcus
Capnocytophaga
Actinomyces
Aggregatibacter
Helicobacter
Veillonella
Lactococcus
Eubacterium
Bacteroides
Parabacteroides
Clostridium
Ruminococcus
Propionibacterium
Campylobacter
Fusobacterium
Selenomonas
Peptoniphilus
Porphyromonas
Prevotella
Haemophilus
Rothia
Lactobacillus
Neisseria
Leptotrichia
Streptococcus
global Kendall's W
Buccal_mucosa - SRS016349
Buccal_mucosa - SRS014126
Buccal_mucosa - SRS015985
Buccal_mucosa - SRS013506
Buccal_mucosa - SRS015895
Buccal_mucosa - SRS015921
Buccal_mucosa - SRS014890
Buccal_mucosa - SRS020858
Buccal_mucosa - SRS018661
Buccal_mucosa - SRS013881
Buccal_mucosa - SRS019976
Buccal_mucosa - SRS016503
Buccal_mucosa - SRS016533
Buccal_mucosa - SRS014686
Buccal_mucosa - SRS015274
Buccal_mucosa - SRS015374
Buccal_mucosa - SRS014472
Buccal_mucosa - SRS014575
Buccal_mucosa - SRS011144
Buccal_mucosa - SRS012281
Buccal_mucosa - SRS050007
Buccal_mucosa - SRS011090
Buccal_mucosa - SRS016196
Buccal_mucosa - SRS048719
Buccal_mucosa - SRS019391
Buccal_mucosa - SRS045049
Buccal_mucosa - SRS019221
Buccal_mucosa - SRS016039
Buccal_mucosa - SRS015154
Buccal_mucosa - SRS015540
Buccal_mucosa - SRS013945
Buccal_mucosa - SRS055118
Buccal_mucosa - SRS011310
Buccal_mucosa - SRS015436
Buccal_mucosa - SRS016297
Buccal_mucosa - SRS015040
Buccal_mucosa - SRS015745
Buccal_mucosa - SRS017127
Buccal_mucosa - SRS015646
Buccal_mucosa - SRS023354
Buccal_mucosa - SRS018359
Buccal_mucosa - SRS019587
Buccal_mucosa - SRS045262
Buccal_mucosa - SRS045254
Buccal_mucosa - SRS022145
Buccal_mucosa - SRS043239
Buccal_mucosa - SRS020336
Buccal_mucosa - SRS042457
Buccal_mucosa - SRS022721
Buccal_mucosa - SRS043646
Buccal_mucosa - SRS013711
Buccal_mucosa - SRS011247
Buccal_mucosa - SRS013825
Buccal_mucosa - SRS017215
Buccal_mucosa - SRS050029
Buccal_mucosa - SRS017013
Buccal_mucosa - SRS019329
Buccal_mucosa - SRS016600
Buccal_mucosa - SRS018329
Buccal_mucosa - SRS022532
Buccal_mucosa - SRS050628
Buccal_mucosa - SRS017080
Buccal_mucosa - SRS017810
Buccal_mucosa - SRS017441
Buccal_mucosa - SRS017537
Buccal_mucosa - SRS016088
Buccal_mucosa - SRS017687
Buccal_mucosa - SRS018149

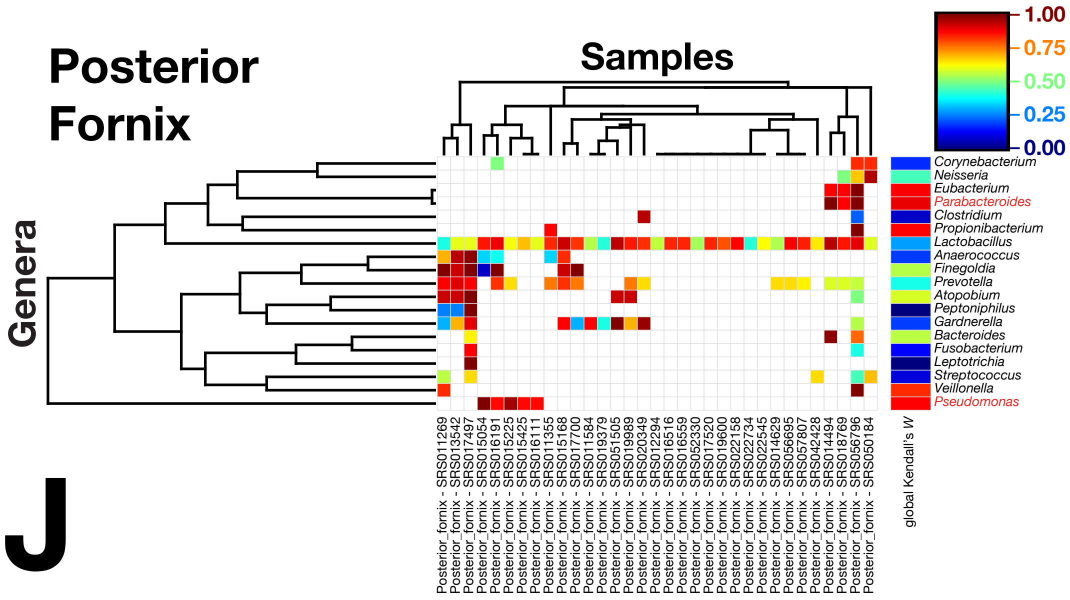

Posterior Fornix
Samples
Genera
J
1.00
0.75
0.50
0.25
0.00
Corynebacterium
Neisseria
Eubacterium
Parabacteroides
Clostridium
Propionibacterium
Lactobacillus
Anaerococcus
Finegoldia
Prevotella
Atopobium
Peptoniphilus
Gardnerella
Bacteroides
Fusobacterium
Leptotrichia
Streptococcus
Veillonella
Pseudomonas
Posterior_fornix - SRS011269
Posterior_fornix - SRS013542
Posterior_fornix - SRS017497
Posterior_fornix - SRS015054
Posterior_fornix - SRS016191
Posterior_fornix - SRS015225
Posterior_fornix - SRS015425
Posterior_fornix - SRS016111
Posterior_fornix - SRS011355
Posterior_fornix - SRS015168
Posterior_fornix - SRS017700
Posterior_fornix - SRS011584
Posterior_fornix - SRS019379
Posterior_fornix - SRS051505
Posterior_fornix - SRS019989
Posterior_fornix - SRS020349
Posterior_fornix - SRS012294
Posterior_fornix - SRS016516
Posterior_fornix - SRS016559
Posterior_fornix - SRS052330
Posterior_fornix - SRS017520
Posterior_fornix - SRS019600
Posterior_fornix - SRS022158
Posterior_fornix - SRS022734
Posterior_fornix - SRS022545
Posterior_fornix - SRS014629
Posterior_fornix - SRS056695
Posterior_fornix - SRS057807
Posterior_fornix - SRS042428
Posterior_fornix - SRS014494
Posterior_fornix - SRS018769
Posterior_fornix - SRS056796
Posterior_fornix - SRS050184
global Kendall's W

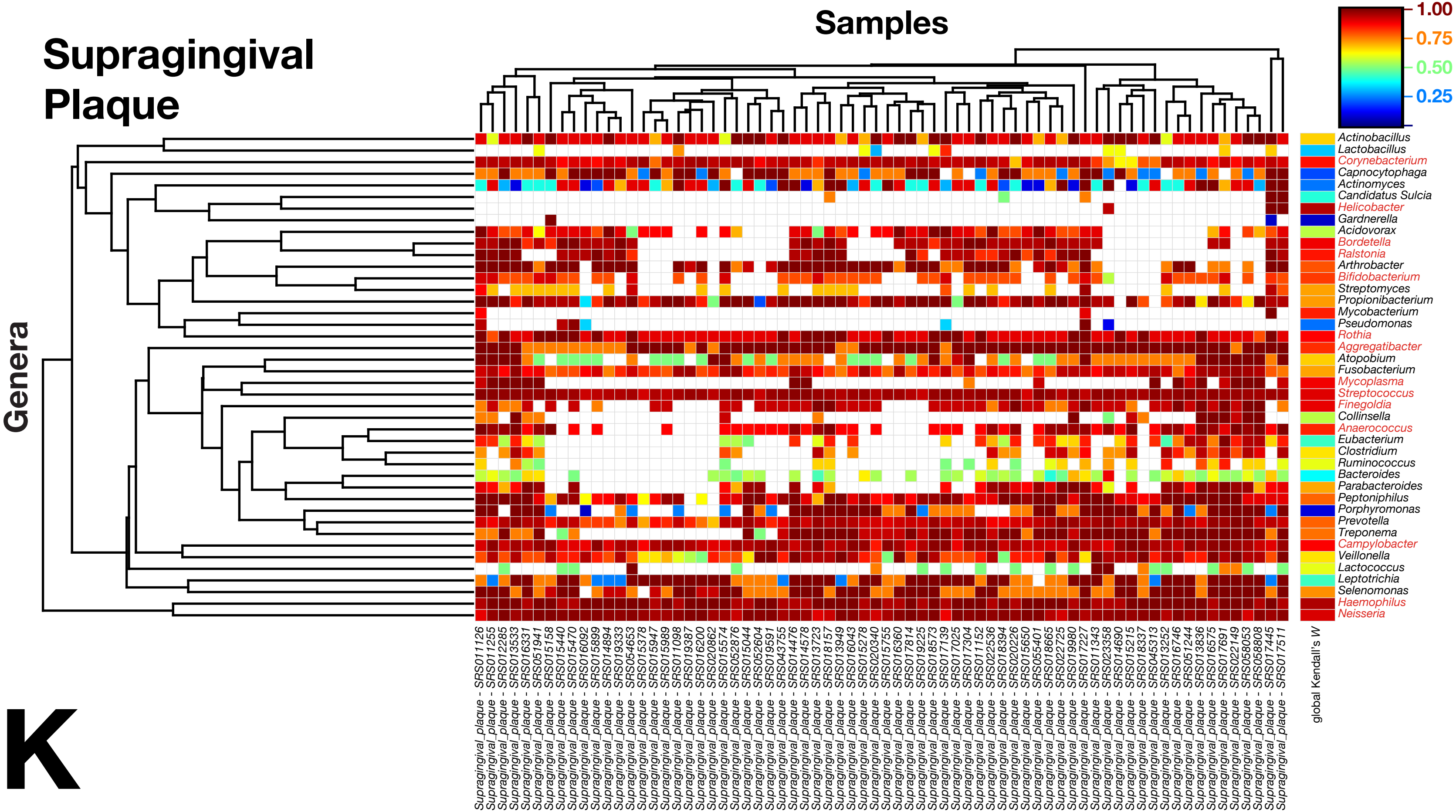
Supragingival Plaque
Samples
Genera
1.00
0.75
0.50
0.25
Actinobacillus
Lactobacillus
Corynebacterium
Capnocytophaga
Actinomyces
Candidatus Sulcia
Helicobacter
Gardnerella
Acidovorax
Bordetella
Ralstonia
Arthrobacter
Bifidobacterium
Streptomyces
Propionibacterium
Mycobacterium
Pseudomonas
Rothia
Aggregatibacter
Atopobium
Fusobacterium
Mycoplasma
Streptococcus
Finegoldia
Collinsella
Anaerococcus
Eubacterium
Clostridium
Ruminococcus
Bacteroides
Parabacteroides
Peptoniphilus
Porphyromonas
Prevotella
Treponema
Campylobacter
Veillonella
Lactococcus
Leptotrichia
Selenomonas
Haemophilus
Neisseria
global Kendall's W
Supragingival_plaque - SRS011126
Supragingival_plaque - SRS011255
Supragingival_plaque - SRS012285
Supragingival_plaque - SRS013533
Supragingival_plaque - SRS016331
Supragingival_plaque - SRS051941
Supragingival_plaque - SRS015158
Supragingival_plaque - SRS015440
Supragingival_plaque - SRS015470
Supragingival_plaque - SRS016092
Supragingival_plaque - SRS015899
Supragingival_plaque - SRS014894
Supragingival_plaque - SRS019333
Supragingival_plaque - SRS054653
Supragingival_plaque - SRS015378
Supragingival_plaque - SRS015947
Supragingival_plaque - SRS015989
Supragingival_plaque - SRS011098
Supragingival_plaque - SRS019387
Supragingival_plaque - SRS016200
Supragingival_plaque - SRS020862
Supragingival_plaque - SRS015574
Supragingival_plaque - SRS052876
Supragingival_plaque - SRS015044
Supragingival_plaque - SRS052604
Supragingival_plaque - SRS019591
Supragingival_plaque - SRS043755
Supragingival_plaque - SRS014476
Supragingival_plaque - SRS014578
Supragingival_plaque - SRS013723
Supragingival_plaque - SRS018157
Supragingival_plaque - SRS013949
Supragingival_plaque - SRS016043
Supragingival_plaque - SRS015278
Supragingival_plaque - SRS020340
Supragingival_plaque - SRS015755
Supragingival_plaque - SRS016360
Supragingival_plaque - SRS017814
Supragingival_plaque - SRS019225
Supragingival_plaque - SRS018573
Supragingival_plaque - SRS017139
Supragingival_plaque - SRS017025
Supragingival_plaque - SRS017304
Supragingival_plaque - SRS011152
Supragingival_plaque - SRS022536
Supragingival_plaque - SRS018394
Supragingival_plaque - SRS020226
Supragingival_plaque - SRS015650
Supragingival_plaque - SRS055401
Supragingival_plaque - SRS018665
Supragingival_plaque - SRS022725
Supragingival_plaque - SRS019980
Supragingival_plaque - SRS017227
Supragingival_plaque - SRS011343
Supragingival_plaque - SRS023358
Supragingival_plaque - SRS014690
Supragingival_plaque - SRS015215
Supragingival_plaque - SRS018337
Supragingival_plaque - SRS045313
Supragingival_plaque - SRS013252
Supragingival_plaque - SRS016746
Supragingival_plaque - SRS051244
Supragingival_plaque - SRS013836
Supragingival_plaque - SRS016575
Supragingival_plaque - SRS017691
Supragingival_plaque - SRS022149
Supragingival_plaque - SRS058053
Supragingival_plaque - SRS058808
Supragingival_plaque - SRS017445
Supragingival_plaque - SRS017511

K

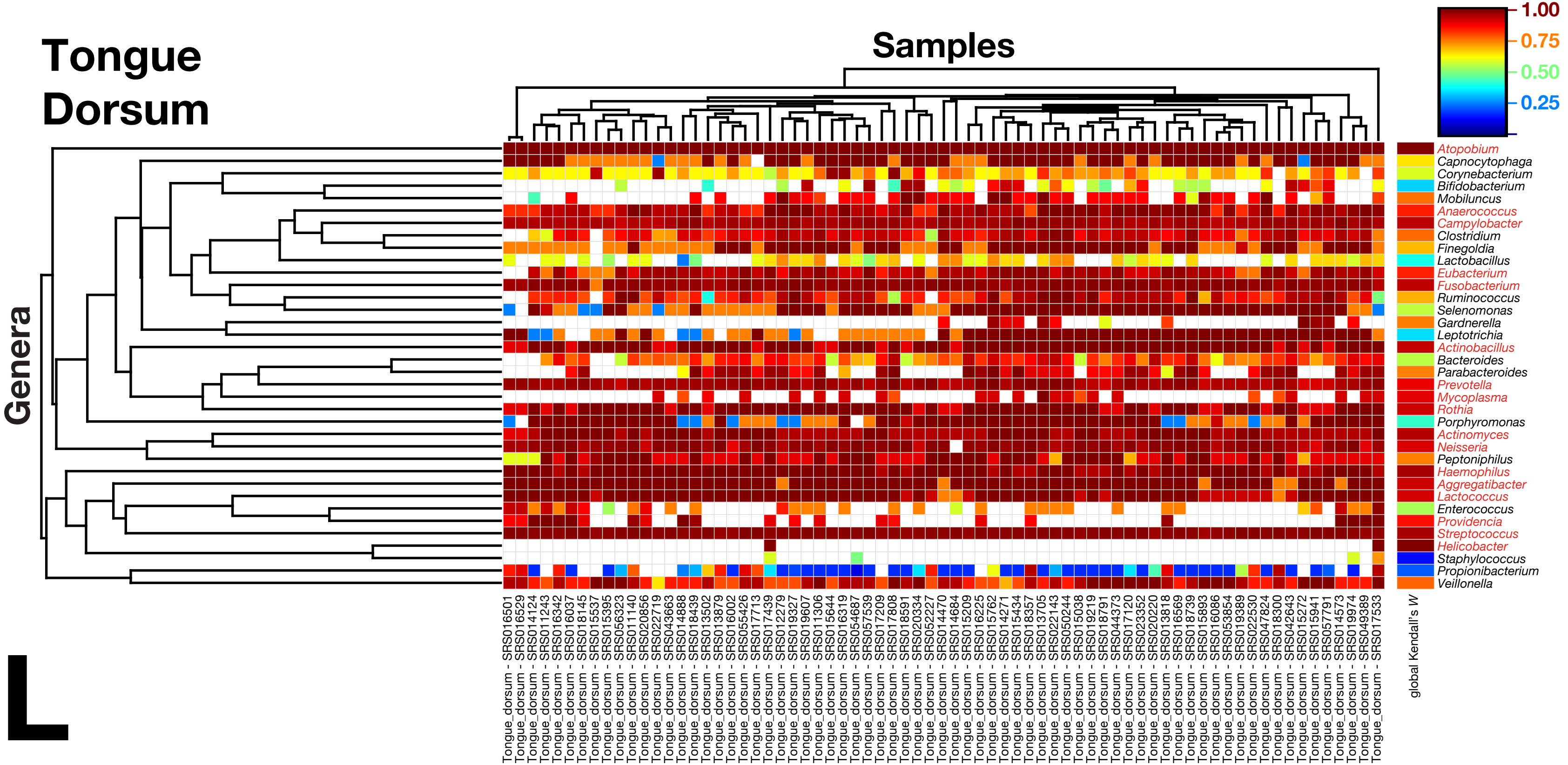
Tongue
Dorsum
Samples
Genera
L
1.00
0.75
0.50
0.25
Atopobium
Capnocytophaga
Corynebacterium
Bifidobacterium
Mobiluncus
Anaerococcus
Campylobacter
Clostridium
Finegoldia
Lactobacillus
Eubacterium
Fusobacterium
Ruminococcus
Selenomonas
Gardnerella
Leptotrichia
Actinobacillus
Bacteroides
Parabacteroides
Prevotella
Mycoplasma
Rothia
Porphyromonas
Actinomyces
Neisseria
Peptoniphilus
Haemophilus
Aggregatibacter
Lactococcus
Enterococcus
Providencia
Streptococcus
Helicobacter
Staphylococcus
Propionibacterium
Veillonella
global Kendall's W
Tongue_dorsum - SRS016501
Tongue_dorsum - SRS016529
Tongue_dorsum - SRS014124
Tongue_dorsum - SRS011243
Tongue_dorsum - SRS016342
Tongue_dorsum - SRS016037
Tongue_dorsum - SRS018145
Tongue_dorsum - SRS015537
Tongue_dorsum - SRS015395
Tongue_dorsum - SRS056323
Tongue_dorsum - SRS011140
Tongue_dorsum - SRS020856
Tongue_dorsum - SRS022719
Tongue_dorsum - SRS043663
Tongue_dorsum - SRS014888
Tongue_dorsum - SRS018439
Tongue_dorsum - SRS013502
Tongue_dorsum - SRS013879
Tongue_dorsum - SRS016002
Tongue_dorsum - SRS055426
Tongue_dorsum - SRS017713
Tongue_dorsum - SRS017439
Tongue_dorsum - SRS012279
Tongue_dorsum - SRS019327
Tongue_dorsum - SRS019607
Tongue_dorsum - SRS011306
Tongue_dorsum - SRS015644
Tongue_dorsum - SRS016319
Tongue_dorsum - SRS054687
Tongue_dorsum - SRS057539
Tongue_dorsum - SRS017209
Tongue_dorsum - SRS017808
Tongue_dorsum - SRS018591
Tongue_dorsum - SRS020334
Tongue_dorsum - SRS052227
Tongue_dorsum - SRS014470
Tongue_dorsum - SRS014684
Tongue_dorsum - SRS015209
Tongue_dorsum - SRS016225
Tongue_dorsum - SRS015762
Tongue_dorsum - SRS014271
Tongue_dorsum - SRS015434
Tongue_dorsum - SRS018357
Tongue_dorsum - SRS013705
Tongue_dorsum - SRS022143
Tongue_dorsum - SRS050244
Tongue_dorsum - SRS015038
Tongue_dorsum - SRS019219
Tongue_dorsum - SRS018791
Tongue_dorsum - SRS044373
Tongue_dorsum - SRS017120
Tongue_dorsum - SRS023352
Tongue_dorsum - SRS020220
Tongue_dorsum - SRS013818
Tongue_dorsum - SRS016569
Tongue_dorsum - SRS018739
Tongue_dorsum - SRS015893
Tongue_dorsum - SRS016086
Tongue_dorsum - SRS053854
Tongue_dorsum - SRS019389
Tongue_dorsum - SRS022530
Tongue_dorsum - SRS047824
Tongue_dorsum - SRS018300
Tongue_dorsum - SRS042643
Tongue_dorsum - SRS015272
Tongue_dorsum - SRS015941
Tongue_dorsum - SRS057791
Tongue_dorsum - SRS014573
Tongue_dorsum - SRS019974
Tongue_dorsum - SRS049389
Tongue_dorsum - SRS017533
