# Supplementary material for: The Prevalence of Species and Strains in the Human Microbiome: A Resource for Experimental Efforts
Source: PLoS One. 2014 May 14;9(5):e97279. doi: 10.1371/journal.pone.0097279 (PMC4020798; doi:10.1371/journal.pone.0097279)
Supplement: Figure S9 — Strain diversity and top 80% by abundance. (A–B) Sample diversity (alpha diversity) visualized in a boxplot using the Simpson index (A) and the Shannon index (B) for each body site. The median diversity is indicated by a horizontal line in the box (covering the 25th until the 75th percentile), the diamond represents the average and the outliers are visualized using dots. The whiskers of the box are the lowest and highest observation of diversity. The Simpson index equals the probability that two strains taken at random from the data set are the same. A high Simpson index equals low diversity. Here the more common 1—Simpson Index is plotted. The Shannon index quantifies the uncertainty (entropy) in predicting the identity of a strain taken at random from the data set. A low Shannon index indicates low strain diversity. The Shannon index was calculated with e as the base of the logarithm. Low diversity can be caused either by a small number of total strains in the data set or by strain domination of the body site. High diversity indicates either a large number of total strains in the data set or a very even distribution of abundance of strains. (C–G) Heat map visualization of the distribution of abundances of the species that are part of the top 80% in at least one sample in the five remaining body sites (see Figure 6 for stool): (C) anterior nares, (D) buccal mucosa, (E) posterior fornix, (F) supragingival plaque and (G) tongue dorsum. Only abundances of 1% or more are visualized to emphasize the differences in prevalence of the most abundant strains. The values are hierarchically clustered using Spearman rank correlation with average linkage. (H) Strain participation in the top 80%. For all the strains that are in the top 80% in at least one sample in a body site the participation over all the samples is given as a percentage for each body site. The heat map of participation percentages is hierarchically clustered using Spearman rank correlation with average linkage [file pone.0097279.s009.pdf]

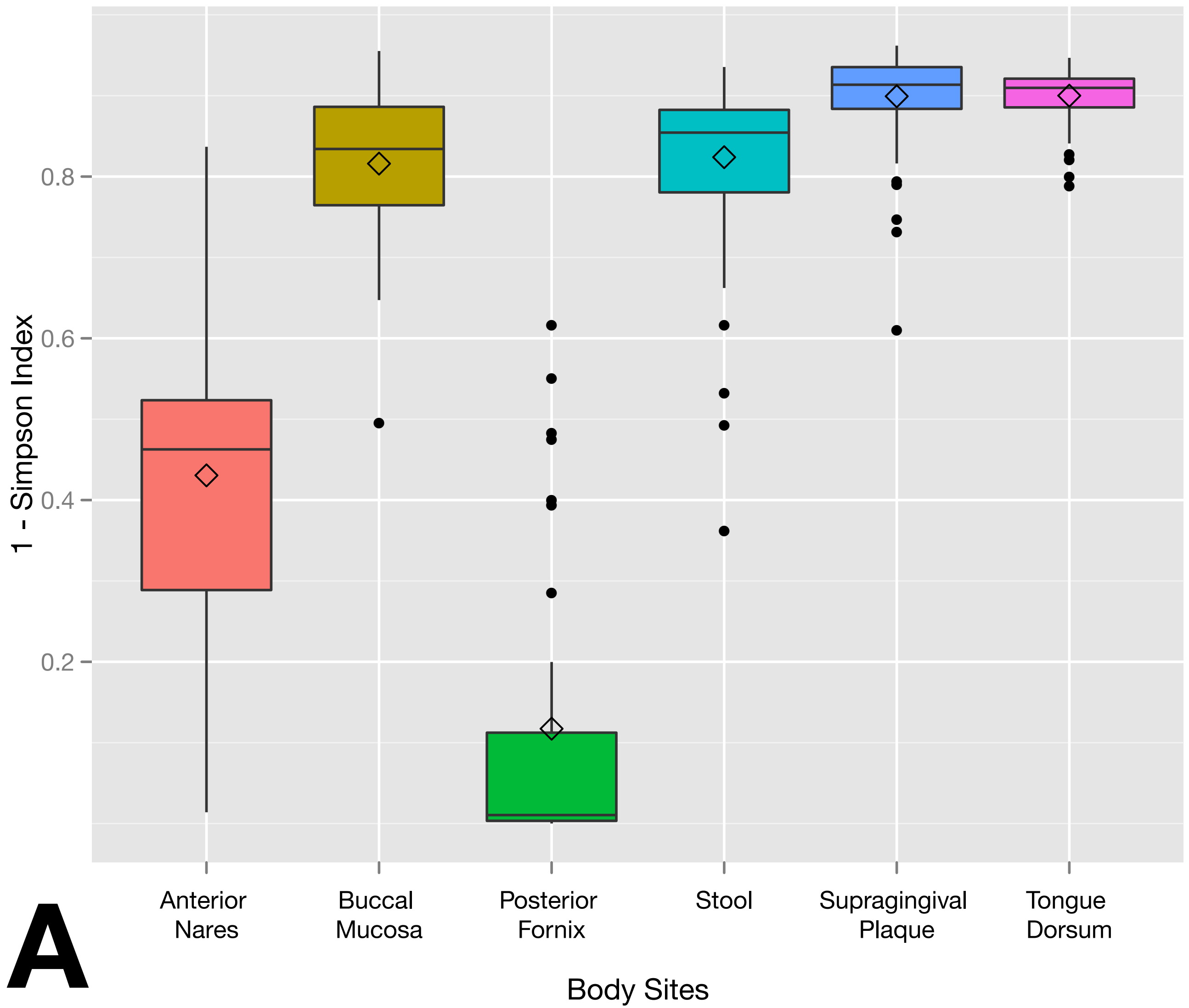
1 - Simpson Index
0.2
0.4
0.6
0.8
Anterior Nares
Buccal Mucosa
Posterior Fornix
Stool
Supragingival Plaque
Tongue Dorsum
Body Sites
A

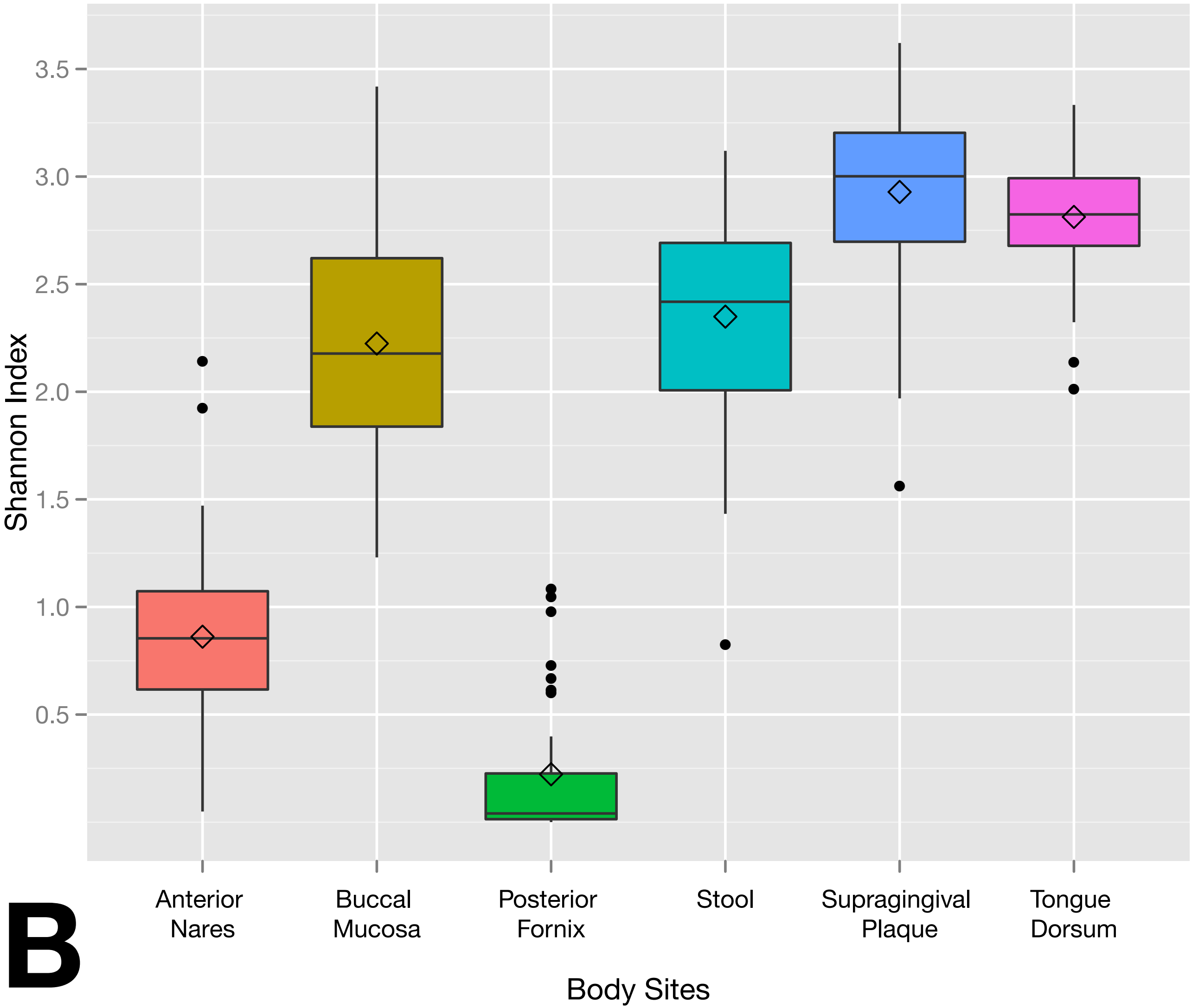

3.5
3.0
2.5
2.0
1.5
1.0
0.5
Shannon Index
Anterior Nares
Buccal Mucosa
Posterior Fornix
Stool
Supragingival Plaque
Tongue Dorsum
Body Sites

B

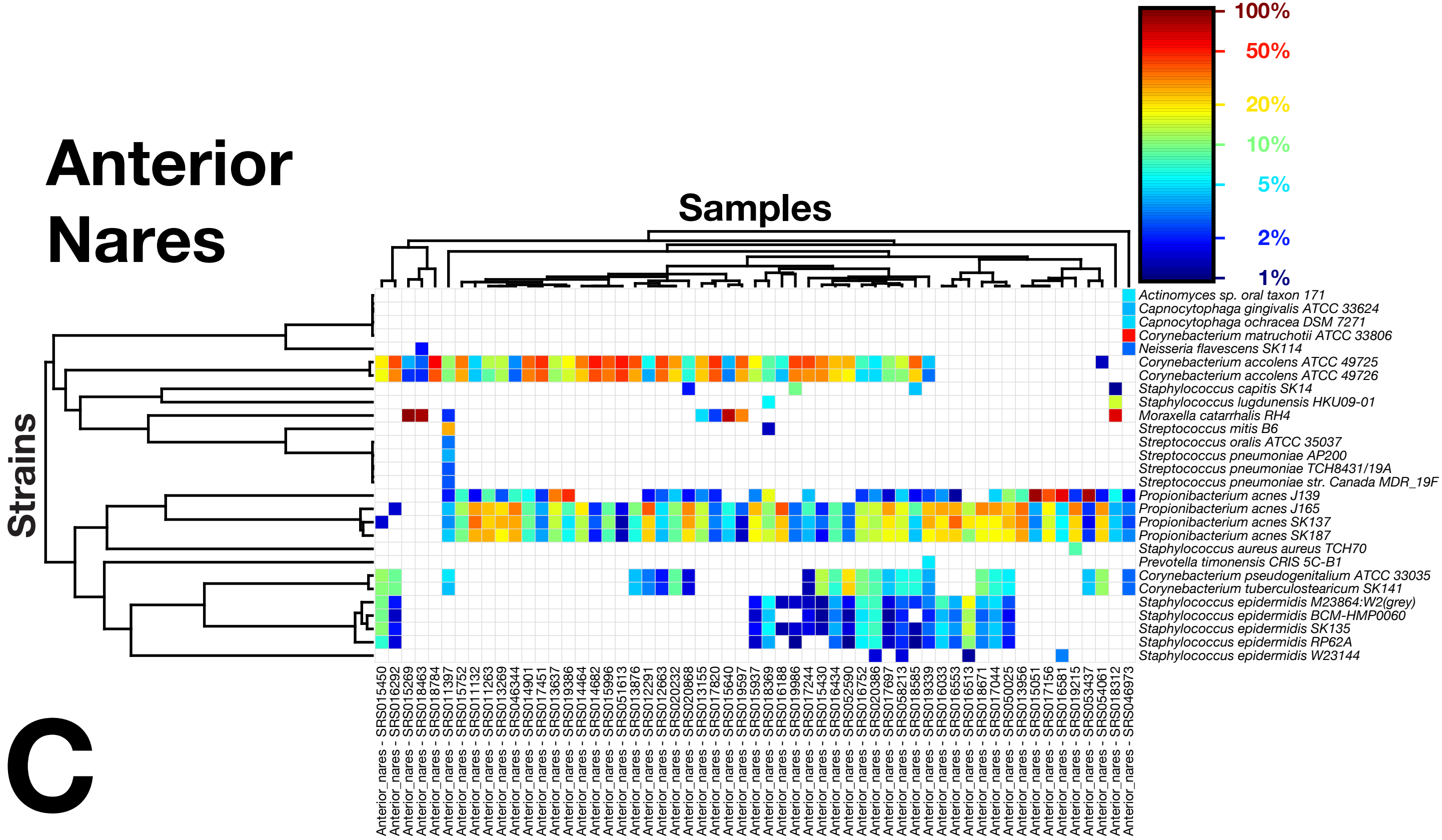
Anterior Nares
Samples
Strains
C
100%
50%
20%
10%
5%
2%
1%
Actinomyces sp. oral taxon 171
Capnocytophaga gingivalis ATCC 33624
Capnocytophaga ochracea DSM 7271
Corynebacterium matruchotii ATCC 33806
Neisseria flavescens SK114
Corynebacterium accolens ATCC 49725
Corynebacterium accolens ATCC 49726
Staphylococcus capitis SK14
Staphylococcus lugdunensis HKU09-01
Moraxella catarrhalis RH4
Streptococcus mitis B6
Streptococcus oralis ATCC 35037
Streptococcus pneumoniae AP200
Streptococcus pneumoniae TCH8431/19A
Streptococcus pneumoniae str. Canada MDR_19F
Propionibacterium acnes J139
Propionibacterium acnes J165
Propionibacterium acnes SK137
Propionibacterium acnes SK187
Staphylococcus aureus aureus TCH70
Prevotella timonensis CRIS 5C-B1
Corynebacterium pseudogenitalium ATCC 33035
Corynebacterium tuberculostearicum SK141
Staphylococcus epidermidis M23864:W2(grey)
Staphylococcus epidermidis BCM-HMP0060
Staphylococcus epidermidis SK135
Staphylococcus epidermidis RP62A
Staphylococcus epidermidis W23144
Anterior_nares - SRS015450
Anterior_nares - SRS016292
Anterior_nares - SRS015269
Anterior_nares - SRS018463
Anterior_nares - SRS018784
Anterior_nares - SRS011397
Anterior_nares - SRS015752
Anterior_nares - SRS011132
Anterior_nares - SRS011263
Anterior_nares - SRS013269
Anterior_nares - SRS046344
Anterior_nares - SRS014901
Anterior_nares - SRS017451
Anterior_nares - SRS013637
Anterior_nares - SRS019386
Anterior_nares - SRS014464
Anterior_nares - SRS014682
Anterior_nares - SRS015996
Anterior_nares - SRS051613
Anterior_nares - SRS013876
Anterior_nares - SRS012291
Anterior_nares - SRS012663
Anterior_nares - SRS020232
Anterior_nares - SRS020868
Anterior_nares - SRS013155
Anterior_nares - SRS017820
Anterior_nares - SRS015640
Anterior_nares - SRS019597
Anterior_nares - SRS015937
Anterior_nares - SRS018369
Anterior_nares - SRS016188
Anterior_nares - SRS019986
Anterior_nares - SRS017244
Anterior_nares - SRS015430
Anterior_nares - SRS016434
Anterior_nares - SRS052590
Anterior_nares - SRS016752
Anterior_nares - SRS020386
Anterior_nares - SRS017697
Anterior_nares - SRS058213
Anterior_nares - SRS018585
Anterior_nares - SRS019339
Anterior_nares - SRS016033
Anterior_nares - SRS016553
Anterior_nares - SRS016513
Anterior_nares - SRS018671
Anterior_nares - SRS017044
Anterior_nares - SRS050025
Anterior_nares - SRS013956
Anterior_nares - SRS015051
Anterior_nares - SRS017156
Anterior_nares - SRS016581
Anterior_nares - SRS019215
Anterior_nares - SRS053437
Anterior_nares - SRS054061
Anterior_nares - SRS018312
Anterior_nares - SRS046973

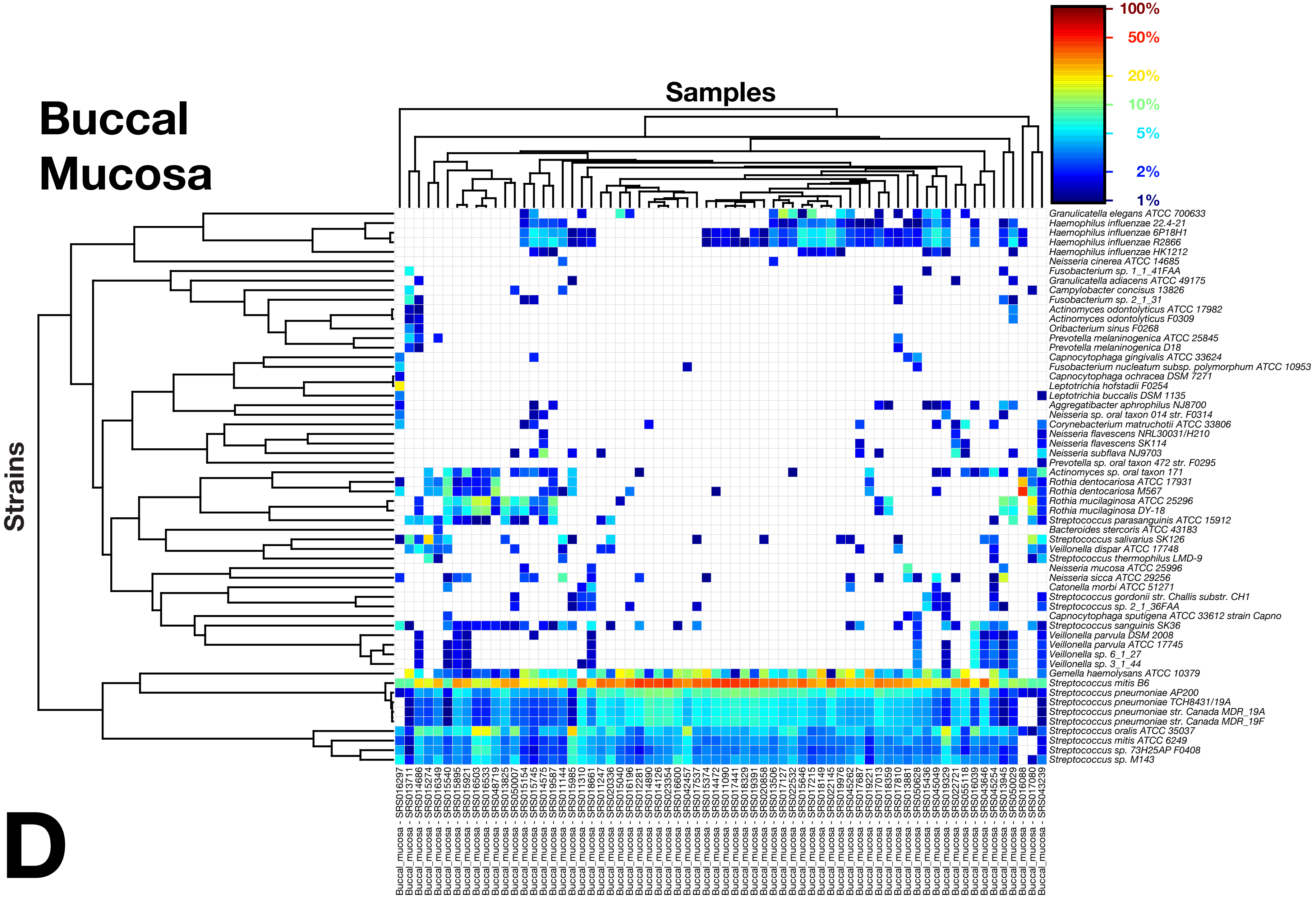
Buccal Mucosa
Samples
Strains
D
100%
50%
20%
10%
5%
2%
1%
Granulicatella elegans ATCC 700633
Haemophilus influenzae 22.4-21
Haemophilus influenzae 6P18H1
Haemophilus influenzae R2866
Haemophilus influenzae HK1212
Neisseria cinerea ATCC 14685
Fusobacterium sp. 1_1_41FAA
Granulicatella adiacens ATCC 49175
Campylobacter concisus 13826
Fusobacterium sp. 2_1_31
Actinomyces odontolyticus ATCC 17982
Actinomyces odontolyticus F0309
Oribacterium sinus F0268
Prevotella melaninogenica ATCC 25845
Prevotella melaninogenica D18
Capnocytophaga gingivalis ATCC 33624
Fusobacterium nucleatum subsp. polymorphum ATCC 10953
Capnocytophaga ochracea DSM 7271
Leptotrichia hofstadii F0254
Leptotrichia buccalis DSM 1135
Aggregatibacter aphrophilus NJ8700
Neisseria sp. oral taxon 014 str. F0314
Corynebacterium matruchotii ATCC 33806
Neisseria flavescens NRL30031/H210
Neisseria flavescens SK114
Neisseria subflava NJ9703
Prevotella sp. oral taxon 472 str. F0295
Actinomyces sp. oral taxon 171
Rothia dentocariosa ATCC 17931
Rothia dentocariosa M567
Rothia mucilaginosa ATCC 25296
Rothia mucilaginosa DY-18
Streptococcus parasanguinis ATCC 15912
Bacteroides stercoris ATCC 43183
Streptococcus salivarius SK126
Veillonella dispar ATCC 17748
Streptococcus thermophilus LMD-9
Neisseria mucosa ATCC 25996
Neisseria sicca ATCC 29256
Catonella morbi ATCC 51271
Streptococcus gordonii str. Challis substr. CH1
Streptococcus sp. 2_1_36FAA
Capnocytophaga sputigena ATCC 33612 strain Capno
Streptococcus sanguinis SK36
Veillonella parvula DSM 2008
Veillonella parvula ATCC 17745
Veillonella sp. 6_1_27
Veillonella sp. 3_1_44
Gemella haemolysans ATCC 10379
Streptococcus mitis B6
Streptococcus pneumoniae AP200
Streptococcus pneumoniae TCH8431/19A
Streptococcus pneumoniae str. Canada MDR_19A
Streptococcus pneumoniae str. Canada MDR_19F
Streptococcus oralis ATCC 35037
Streptococcus mitis ATCC 6249
Streptococcus sp. 73H25AP F0408
Streptococcus sp. M143
Buccal_mucosa - SRS016297
Buccal_mucosa - SRS013711
Buccal_mucosa - SRS014686
Buccal_mucosa - SRS015274
Buccal_mucosa - SRS016349
Buccal_mucosa - SRS015540
Buccal_mucosa - SRS015895
Buccal_mucosa - SRS015921
Buccal_mucosa - SRS016503
Buccal_mucosa - SRS016533
Buccal_mucosa - SRS048719
Buccal_mucosa - SRS013825
Buccal_mucosa - SRS050007
Buccal_mucosa - SRS015154
Buccal_mucosa - SRS015745
Buccal_mucosa - SRS014575
Buccal_mucosa - SRS019587
Buccal_mucosa - SRS011144
Buccal_mucosa - SRS015985
Buccal_mucosa - SRS011310
Buccal_mucosa - SRS018661
Buccal_mucosa - SRS011247
Buccal_mucosa - SRS020336
Buccal_mucosa - SRS015040
Buccal_mucosa - SRS016196
Buccal_mucosa - SRS012281
Buccal_mucosa - SRS014890
Buccal_mucosa - SRS014126
Buccal_mucosa - SRS023354
Buccal_mucosa - SRS016600
Buccal_mucosa - SRS042457
Buccal_mucosa - SRS017537
Buccal_mucosa - SRS015374
Buccal_mucosa - SRS014472
Buccal_mucosa - SRS011090
Buccal_mucosa - SRS017441
Buccal_mucosa - SRS018329
Buccal_mucosa - SRS019391
Buccal_mucosa - SRS020858
Buccal_mucosa - SRS013506
Buccal_mucosa - SRS017127
Buccal_mucosa - SRS022532
Buccal_mucosa - SRS015646
Buccal_mucosa - SRS017215
Buccal_mucosa - SRS018149
Buccal_mucosa - SRS022145
Buccal_mucosa - SRS019976
Buccal_mucosa - SRS045262
Buccal_mucosa - SRS017687
Buccal_mucosa - SRS019221
Buccal_mucosa - SRS017013
Buccal_mucosa - SRS018359
Buccal_mucosa - SRS017810
Buccal_mucosa - SRS013881
Buccal_mucosa - SRS050628
Buccal_mucosa - SRS015436
Buccal_mucosa - SRS045049
Buccal_mucosa - SRS019329
Buccal_mucosa - SRS022721
Buccal_mucosa - SRS055118
Buccal_mucosa - SRS016039
Buccal_mucosa - SRS043646
Buccal_mucosa - SRS045254
Buccal_mucosa - SRS013945
Buccal_mucosa - SRS050029
Buccal_mucosa - SRS016088
Buccal_mucosa - SRS017080
Buccal_mucosa - SRS043239

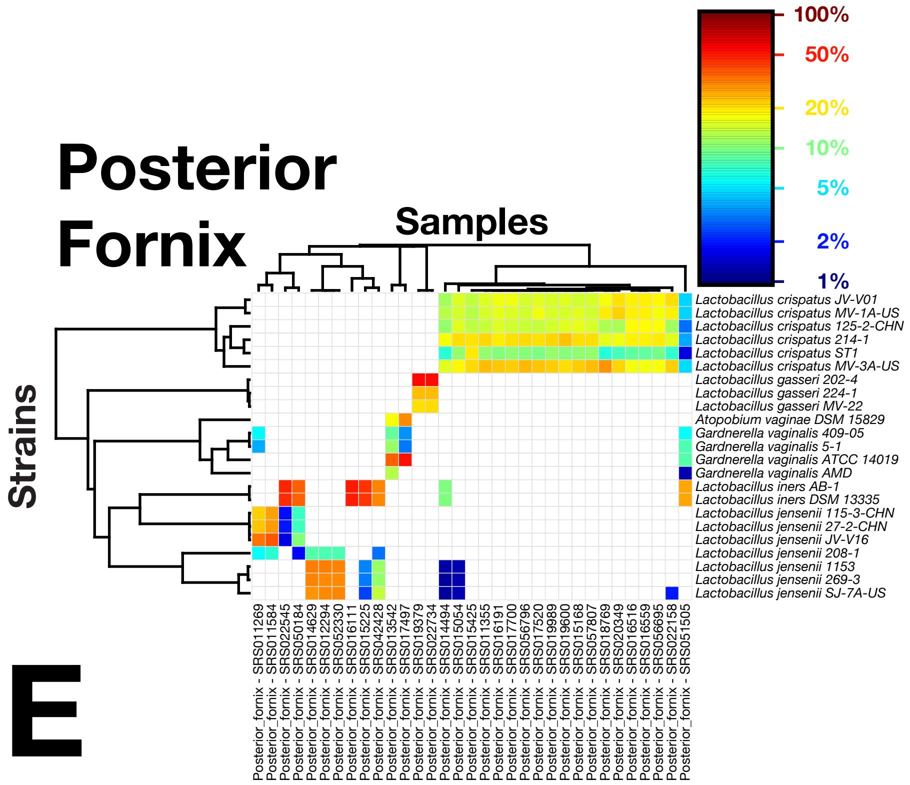
E
Posterior Fornix
Samples
Strains
100%
50%
20%
10%
5%
2%
1%
Lactobacillus crispatus JV-V01
Lactobacillus crispatus MV-1A-US
Lactobacillus crispatus 125-2-CHN
Lactobacillus crispatus 214-1
Lactobacillus crispatus ST1
Lactobacillus crispatus MV-3A-US
Lactobacillus gasseri 202-4
Lactobacillus gasseri 224-1
Lactobacillus gasseri MV-22
Atopobium vaginae DSM 15829
Gardnerella vaginalis 409-05
Gardnerella vaginalis 5-1
Gardnerella vaginalis ATCC 14019
Gardnerella vaginalis AMD
Lactobacillus iners AB-1
Lactobacillus iners DSM 13335
Lactobacillus jensenii 115-3-CHN
Lactobacillus jensenii 27-2-CHN
Lactobacillus jensenii JV-V16
Lactobacillus jensenii 208-1
Lactobacillus jensenii 1153
Lactobacillus jensenii 269-3
Lactobacillus jensenii SJ-7A-US
Posterior_fornix - SRS011269
Posterior_fornix - SRS011584
Posterior_fornix - SRS022545
Posterior_fornix - SRS050184
Posterior_fornix - SRS014629
Posterior_fornix - SRS012294
Posterior_fornix - SRS052330
Posterior_fornix - SRS016111
Posterior_fornix - SRS015225
Posterior_fornix - SRS042428
Posterior_fornix - SRS013542
Posterior_fornix - SRS017497
Posterior_fornix - SRS019379
Posterior_fornix - SRS022734
Posterior_fornix - SRS014494
Posterior_fornix - SRS015054
Posterior_fornix - SRS015425
Posterior_fornix - SRS011355
Posterior_fornix - SRS016191
Posterior_fornix - SRS017700
Posterior_fornix - SRS056796
Posterior_fornix - SRS017520
Posterior_fornix - SRS019989
Posterior_fornix - SRS019600
Posterior_fornix - SRS015168
Posterior_fornix - SRS057807
Posterior_fornix - SRS018769
Posterior_fornix - SRS020349
Posterior_fornix - SRS016516
Posterior_fornix - SRS016559
Posterior_fornix - SRS056695
Posterior_fornix - SRS022158
Posterior_fornix - SRS051505

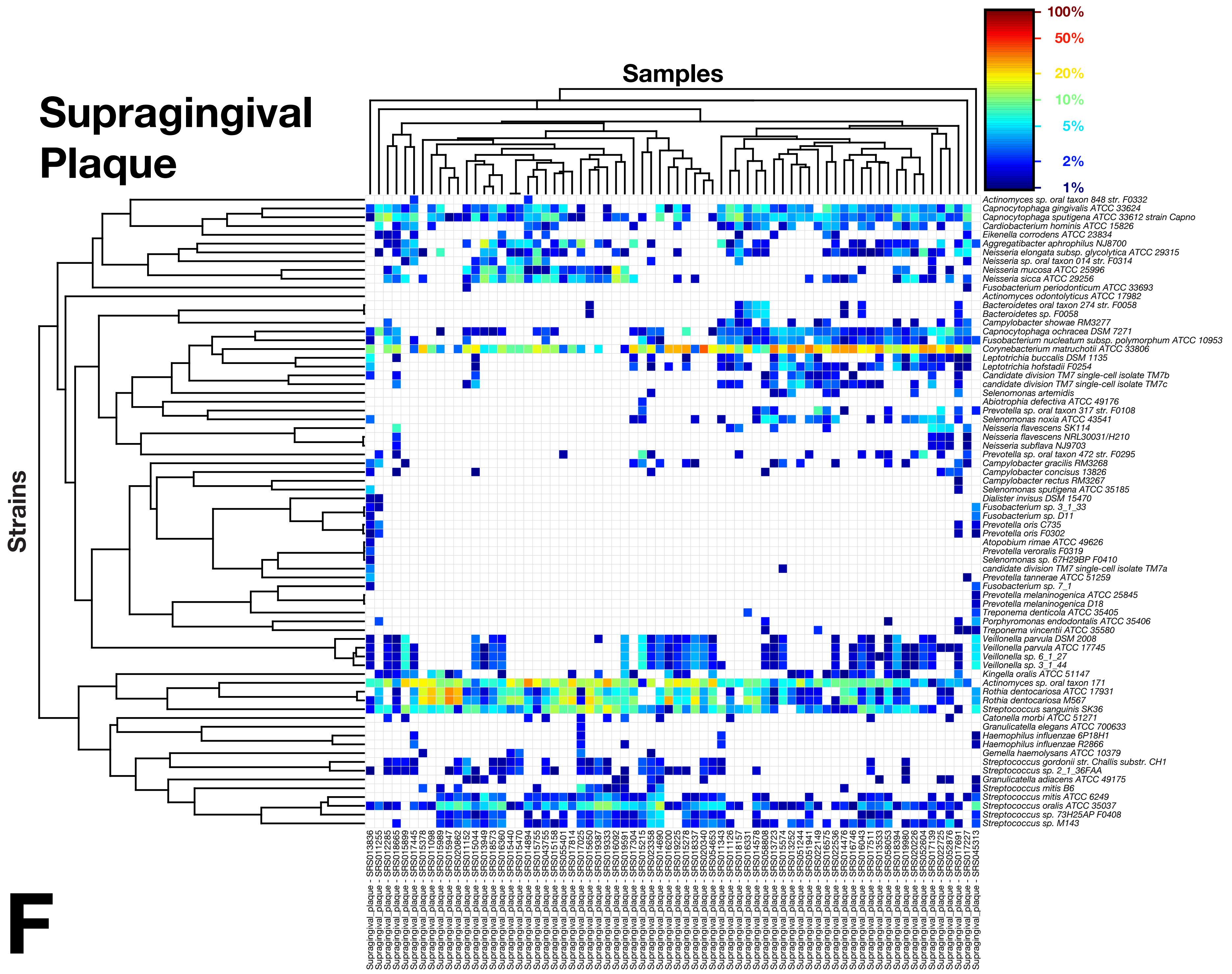

Supragingival Plaque
Samples
Strains
F
100%
50%
20%
10%
5%
2%
1%
Actinomyces sp. oral taxon 848 str. F0332
Capnocytophaga gingivalis ATCC 33624
Capnocytophaga sputigena ATCC 33612 strain Capno
Cardiobacterium hominis ATCC 15826
Eikenella corrodens ATCC 23834
Aggregatibacter aphrophilus NJ8700
Neisseria elongata subsp. glycolytica ATCC 29315
Neisseria sp. oral taxon 014 str. F0314
Neisseria mucosa ATCC 25996
Neisseria sicca ATCC 29256
Fusobacterium periodonticum ATCC 33693
Actinomyces odontolyticus ATCC 17982
Bacteroidetes oral taxon 274 str. F0058
Bacteroidetes sp. F0058
Campylobacter showae RM3277
Capnocytophaga ochracea DSM 7271
Fusobacterium nucleatum subsp. polymorphum ATCC 10953
Corynebacterium matruchotii ATCC 33806
Leptotrichia buccalis DSM 1135
Leptotrichia hofstadii F0254
Candidate division TM7 single-cell isolate TM7b
candidate division TM7 single-cell isolate TM7c
Selenomonas artemidis
Abiotrophia defectiva ATCC 49176
Prevotella sp. oral taxon 317 str. F0108
Selenomonas noxia ATCC 43541
Neisseria flavescens SK114
Neisseria flavescens NRL30031/H210
Neisseria subflava NJ9703
Prevotella sp. oral taxon 472 str. F0295
Campylobacter gracilis RM3268
Campylobacter concisus 13826
Campylobacter rectus RM3267
Selenomonas sputigena ATCC 35185
Dialister invisus DSM 15470
Fusobacterium sp. 3_1_33
Fusobacterium sp. D11
Prevotella oris C735
Prevotella oris F0302
Atopobium rimae ATCC 49626
Prevotella veroralis F0319
Selenomonas sp. 67H29BP F0410
candidate division TM7 single-cell isolate TM7a
Prevotella tannerae ATCC 51259
Fusobacterium sp. 7_1
Prevotella melaninogenica ATCC 25845
Prevotella melaninogenica D18
Treponema denticola ATCC 35405
Porphyromonas endodontalis ATCC 35406
Treponema vincentii ATCC 35580
Veillonella parvula DSM 2008
Veillonella parvula ATCC 17745
Veillonella sp. 6_1_27
Veillonella sp. 3_1_44
Kingella oralis ATCC 51147
Actinomyces sp. oral taxon 171
Rothia dentocariosa ATCC 17931
Rothia dentocariosa M567
Streptococcus sanguinis SK36
Catonella morbi ATCC 51271
Granulicatella elegans ATCC 700633
Haemophilus influenzae 6P18H1
Haemophilus influenzae R2866
Gemella haemolysans ATCC 10379
Streptococcus gordonii str. Challis substr. CH1
Streptococcus sp. 2_1_36FAA
Granulicatella adiacens ATCC 49175
Streptococcus mitis B6
Streptococcus mitis ATCC 6249
Streptococcus oralis ATCC 35037
Streptococcus sp. 73H25AP F0408
Streptococcus sp. M143
Supragingival_plaque - SRS013836
Supragingival_plaque - SRS011255
Supragingival_plaque - SRS012285
Supragingival_plaque - SRS018665
Supragingival_plaque - SRS015899
Supragingival_plaque - SRS017445
Supragingival_plaque - SRS015378
Supragingival_plaque - SRS011098
Supragingival_plaque - SRS015989
Supragingival_plaque - SRS015947
Supragingival_plaque - SRS020862
Supragingival_plaque - SRS011152
Supragingival_plaque - SRS015044
Supragingival_plaque - SRS013949
Supragingival_plaque - SRS018573
Supragingival_plaque - SRS016360
Supragingival_plaque - SRS015440
Supragingival_plaque - SRS015470
Supragingival_plaque - SRS014894
Supragingival_plaque - SRS015755
Supragingival_plaque - SRS043755
Supragingival_plaque - SRS015158
Supragingival_plaque - SRS055401
Supragingival_plaque - SRS017814
Supragingival_plaque - SRS017025
Supragingival_plaque - SRS015650
Supragingival_plaque - SRS019387
Supragingival_plaque - SRS019333
Supragingival_plaque - SRS016092
Supragingival_plaque - SRS019591
Supragingival_plaque - SRS017304
Supragingival_plaque - SRS015215
Supragingival_plaque - SRS023358
Supragingival_plaque - SRS014690
Supragingival_plaque - SRS016200
Supragingival_plaque - SRS019225
Supragingival_plaque - SRS015278
Supragingival_plaque - SRS018337
Supragingival_plaque - SRS020340
Supragingival_plaque - SRS054653
Supragingival_plaque - SRS011343
Supragingival_plaque - SRS011126
Supragingival_plaque - SRS018157
Supragingival_plaque - SRS016331
Supragingival_plaque - SRS014578
Supragingival_plaque - SRS058808
Supragingival_plaque - SRS013723
Supragingival_plaque - SRS015574
Supragingival_plaque - SRS013252
Supragingival_plaque - SRS051244
Supragingival_plaque - SRS051941
Supragingival_plaque - SRS022149
Supragingival_plaque - SRS016575
Supragingival_plaque - SRS022536
Supragingival_plaque - SRS014476
Supragingival_plaque - SRS016746
Supragingival_plaque - SRS016043
Supragingival_plaque - SRS017511
Supragingival_plaque - SRS013533
Supragingival_plaque - SRS058053
Supragingival_plaque - SRS018394
Supragingival_plaque - SRS019980
Supragingival_plaque - SRS020226
Supragingival_plaque - SRS052604
Supragingival_plaque - SRS017139
Supragingival_plaque - SRS022725
Supragingival_plaque - SRS052876
Supragingival_plaque - SRS017691
Supragingival_plaque - SRS017227
Supragingival_plaque - SRS045313

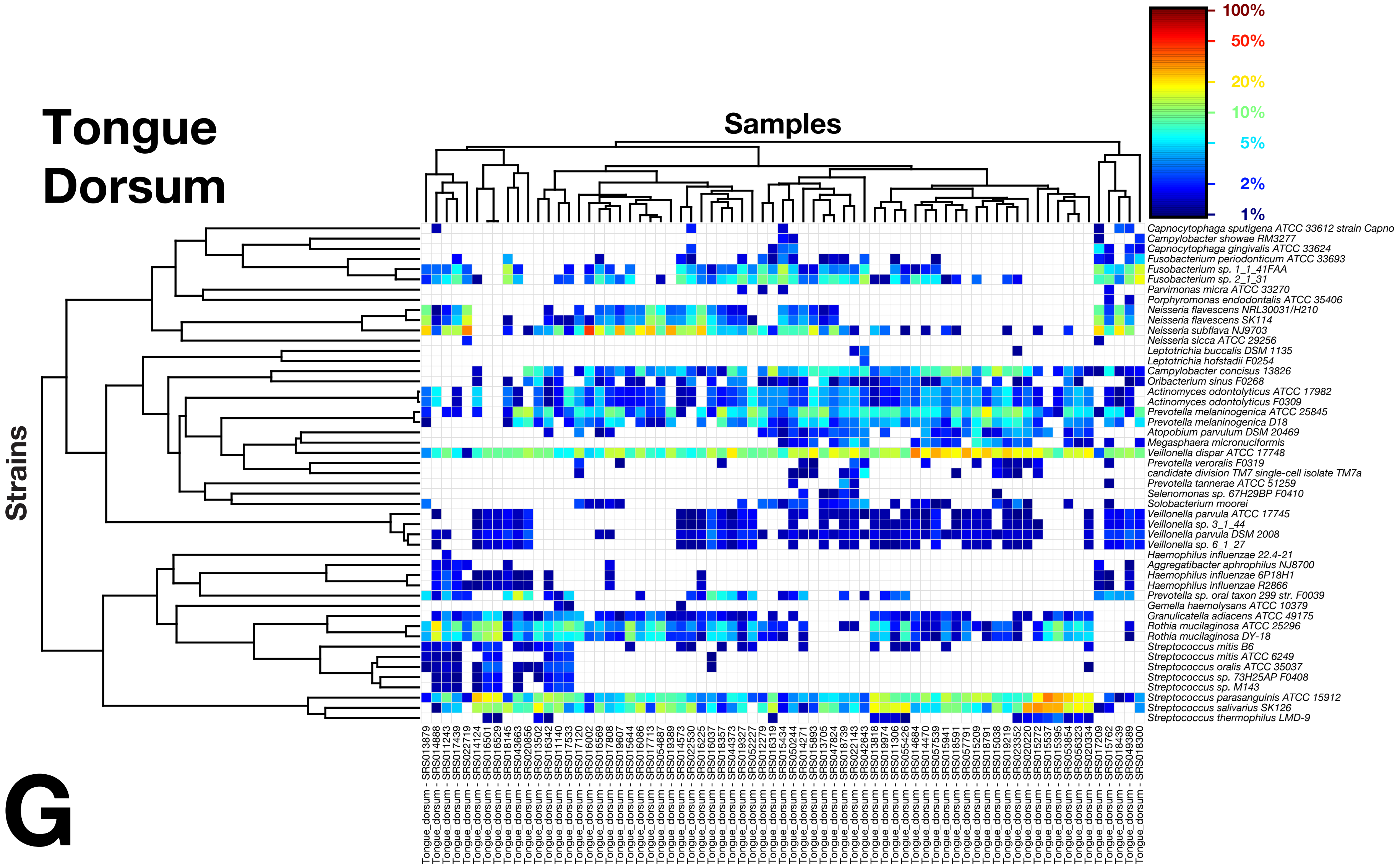

Tongue
Dorsum
Samples
Strains
G
100%
50%
20%
10%
5%
2%
1%
Capnocytophaga sputigena ATCC 33612 strain Capno
Campylobacter showae RM3277
Capnocytophaga gingivalis ATCC 33624
Fusobacterium periodonticum ATCC 33693
Fusobacterium sp. 1_1_41FAA
Fusobacterium sp. 2_1_31
Parvimonas micra ATCC 33270
Porphyromonas endodontalis ATCC 35406
Neisseria flavescens NRL30031/H210
Neisseria flavescens SK114
Neisseria subflava NJ9703
Neisseria sicca ATCC 29256
Leptotrichia buccalis DSM 1135
Leptotrichia hofstadii F0254
Campylobacter concisus 13826
Oribacterium sinus F0268
Actinomyces odontolyticus ATCC 17982
Actinomyces odontolyticus F0309
Prevotella melaninogenica ATCC 25845
Prevotella melaninogenica D18
Atopobium parvulum DSM 20469
Megasphaera micronuciformis
Veillonella dispar ATCC 17748
Prevotella veroralis F0319
candidate division TM7 single-cell isolate TM7a
Prevotella tannerae ATCC 51259
Selenomonas sp. 67H29BP F0410
Solobacterium moorei
Veillonella parvula ATCC 17745
Veillonella sp. 3_1_44
Veillonella parvula DSM 2008
Veillonella sp. 6_1_27
Haemophilus influenzae 22.4-21
Aggregatibacter aphrophilus NJ8700
Haemophilus influenzae 6P18H1
Haemophilus influenzae R2866
Prevotella sp. oral taxon 299 str. F0039
Gemella haemolysans ATCC 10379
Granulicatella adiacens ATCC 49175
Rothia mucilaginosa ATCC 25296
Rothia mucilaginosa DY-18
Streptococcus mitis B6
Streptococcus mitis ATCC 6249
Streptococcus oralis ATCC 35037
Streptococcus sp. 73H25AP F0408
Streptococcus sp. M143
Streptococcus parasanguinis ATCC 15912
Streptococcus salivarius SK126
Streptococcus thermophilus LMD-9
Tongue_dorsum - SRS013879
Tongue_dorsum - SRS014888
Tongue_dorsum - SRS011243
Tongue_dorsum - SRS017439
Tongue_dorsum - SRS022719
Tongue_dorsum - SRS014124
Tongue_dorsum - SRS016501
Tongue_dorsum - SRS016529
Tongue_dorsum - SRS018145
Tongue_dorsum - SRS043663
Tongue_dorsum - SRS020856
Tongue_dorsum - SRS013502
Tongue_dorsum - SRS016342
Tongue_dorsum - SRS011140
Tongue_dorsum - SRS017533
Tongue_dorsum - SRS017120
Tongue_dorsum - SRS016002
Tongue_dorsum - SRS016569
Tongue_dorsum - SRS017808
Tongue_dorsum - SRS019607
Tongue_dorsum - SRS015644
Tongue_dorsum - SRS016086
Tongue_dorsum - SRS017713
Tongue_dorsum - SRS054687
Tongue_dorsum - SRS019389
Tongue_dorsum - SRS014573
Tongue_dorsum - SRS022530
Tongue_dorsum - SRS016225
Tongue_dorsum - SRS016037
Tongue_dorsum - SRS018357
Tongue_dorsum - SRS044373
Tongue_dorsum - SRS019327
Tongue_dorsum - SRS052227
Tongue_dorsum - SRS012279
Tongue_dorsum - SRS016319
Tongue_dorsum - SRS015434
Tongue_dorsum - SRS050244
Tongue_dorsum - SRS014271
Tongue_dorsum - SRS015893
Tongue_dorsum - SRS013705
Tongue_dorsum - SRS047824
Tongue_dorsum - SRS018739
Tongue_dorsum - SRS022143
Tongue_dorsum - SRS042643
Tongue_dorsum - SRS013818
Tongue_dorsum - SRS019974
Tongue_dorsum - SRS011306
Tongue_dorsum - SRS055426
Tongue_dorsum - SRS014684
Tongue_dorsum - SRS014470
Tongue_dorsum - SRS057539
Tongue_dorsum - SRS015941
Tongue_dorsum - SRS018591
Tongue_dorsum - SRS057791
Tongue_dorsum - SRS015209
Tongue_dorsum - SRS018791
Tongue_dorsum - SRS015038
Tongue_dorsum - SRS019219
Tongue_dorsum - SRS023352
Tongue_dorsum - SRS020220
Tongue_dorsum - SRS015272
Tongue_dorsum - SRS015537
Tongue_dorsum - SRS015395
Tongue_dorsum - SRS053854
Tongue_dorsum - SRS056323
Tongue_dorsum - SRS020334
Tongue_dorsum - SRS017209
Tongue_dorsum - SRS015762
Tongue_dorsum - SRS018439
Tongue_dorsum - SRS049389
Tongue_dorsum - SRS018300

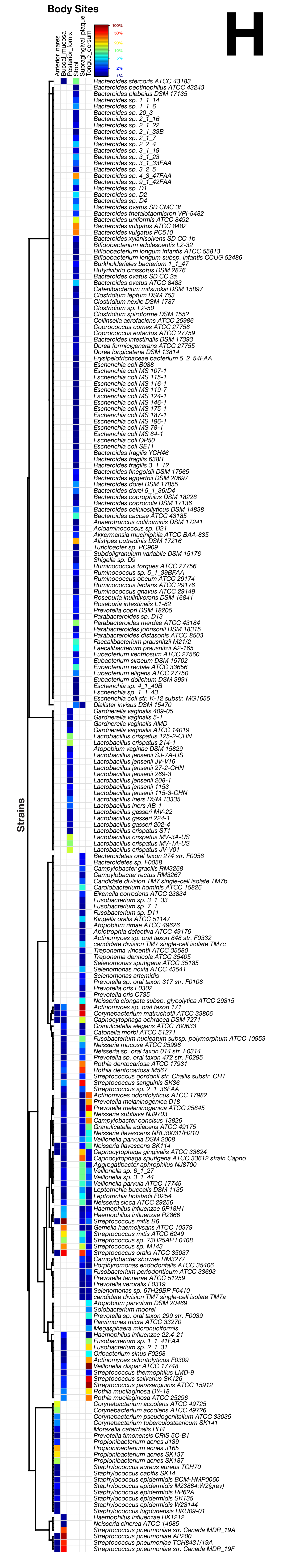

Body Sites
H
Anterior_nares
Buccal_mucosa
Posterior_fornix
Stool
Supragingival_plaque
Tongue_dorsum
100%
50%
20%
10%
5%
2%
1%
Strains
Bacteroides stercoris ATCC 43183
Bacteroides pectinophilus ATCC 43243
Bacteroides plebeius DSM 17135
Bacteroides sp. 1_1_14
Bacteroides sp. 1_1_6
Bacteroides sp. 20_3
Bacteroides sp. 2_1_16
Bacteroides sp. 2_1_22
Bacteroides sp. 2_1_33B
Bacteroides sp. 2_1_7
Bacteroides sp. 2_2_4
Bacteroides sp. 3_1_19
Bacteroides sp. 3_1_23
Bacteroides sp. 3_1_33FAA
Bacteroides sp. 3_2_5
Bacteroides sp. 4_3_47FAA
Bacteroides sp. 9_1_42FAA
Bacteroides sp. D1
Bacteroides sp. D2
Bacteroides sp. D4
Bacteroides ovatus SD CMC 3f
Bacteroides thetaiotaomicron VPI-5482
Bacteroides uniformis ATCC 8492
Bacteroides vulgatus ATCC 8482
Bacteroides vulgatus PC510
Bacteroides xylanisolvens SD CC 1b
Bifidobacterium adolescentis L2-32
Bifidobacterium longum infantis ATCC 55813
Bifidobacterium longum subsp. infantis CCUG 52486
Burkholderiales bacterium 1_1_47
Butyrivibrio crossotus DSM 2876
Bacteroides ovatus SD CC 2a
Bacteroides ovatus ATCC 8483
Catenibacterium mitsuokai DSM 15897
Clostridium leptum DSM 753
Clostridium nexile DSM 1787
Clostridium sp. L2-50
Clostridium spiroforme DSM 1552
Collinsella aerofaciens ATCC 25986
Coprococcus comes ATCC 27758
Coprococcus eutactus ATCC 27759
Bacteroides intestinalis DSM 17393
Dorea formicigenerans ATCC 27755
Dorea longicatena DSM 13814
Erysipelotrichaceae bacterium 5_2_54FAA
Escherichia coli B088
Escherichia coli MS 107-1
Escherichia coli MS 115-1
Escherichia coli MS 116-1
Escherichia coli MS 119-7
Escherichia coli MS 124-1
Escherichia coli MS 146-1
Escherichia coli MS 175-1
Escherichia coli MS 187-1
Escherichia coli MS 196-1
Escherichia coli MS 78-1
Escherichia coli MS 84-1
Escherichia coli OP50
Escherichia coli SE11
Bacteroides fragilis YCH46
Bacteroides fragilis 638R
Bacteroides fragilis 3_1_12
Bacteroides finegoldii DSM 17565
Bacteroides eggerthii DSM 20697
Bacteroides dorei DSM 17855
Bacteroides dorei 5_1_36/D4
Bacteroides coprophilus DSM 18228
Bacteroides coprocola DSM 17136
Bacteroides cellulosilyticus DSM 14838
Bacteroides caccae ATCC 43185
Anaerotruncus colihominis DSM 17241
Acidaminococcus sp. D21
Akkermansia muciniphila ATCC BAA-835
Alistipes putredinis DSM 17216
Turicibacter sp. PC909
Subdoligranulum variabile DSM 15176
Shigella sp. D9
Ruminococcus torques ATCC 27756
Ruminococcus sp. 5_1_39BFAA
Ruminococcus obeum ATCC 29174
Ruminococcus lactaris ATCC 29176
Ruminococcus gnavus ATCC 29149
Roseburia inulinivorans DSM 16841
Roseburia intestinalis L1-82
Prevotella copri DSM 18205
Parabacteroides sp. D13
Parabacteroides merdae ATCC 43184
Parabacteroides johnsonii DSM 18315
Parabacteroides distasonis ATCC 8503
Faecalibacterium prausnitzii M21/2
Faecalibacterium prausnitzii A2-165
Eubacterium ventriosum ATCC 27560
Eubacterium siraeum DSM 15702
Eubacterium rectale ATCC 33656
Eubacterium eligens ATCC 27750
Eubacterium dolichum DSM 3991
Escherichia sp. 4_1_40B
Escherichia sp. 1_1_43
Escherichia coli str. K-12 substr. MG1655
Dialister invisus DSM 15470
Gardnerella vaginalis 409-05
Gardnerella vaginalis 5-1
Gardnerella vaginalis AMD
Gardnerella vaginalis ATCC 14019
Lactobacillus crispatus 125-2-CHN
Lactobacillus crispatus 214-1
Atopobium vaginae DSM 15829
Lactobacillus jensenii SJ-7A-US
Lactobacillus jensenii JV-V16
Lactobacillus jensenii 27-2-CHN
Lactobacillus jensenii 269-3
Lactobacillus jensenii 208-1
Lactobacillus jensenii 1153
Lactobacillus jensenii 115-3-CHN
Lactobacillus iners DSM 13335
Lactobacillus iners AB-1
Lactobacillus gasseri MV-22
Lactobacillus gasseri 224-1
Lactobacillus gasseri 202-4
Lactobacillus crispatus ST1
Lactobacillus crispatus MV-3A-US
Lactobacillus crispatus MV-1A-US
Lactobacillus crispatus JV-V01
Bacteroidetes oral taxon 274 str. F0058
Bacteroidetes sp. F0058
Campylobacter gracilis RM3268
Campylobacter rectus RM3267
Candidate division TM7 single-cell isolate TM7b
Cardiobacterium hominis ATCC 15826
Eikenella corrodens ATCC 23834
Fusobacterium sp. 3_1_33
Fusobacterium sp. 7_1
Fusobacterium sp. D11
Kingella oralis ATCC 51147
Atopobium rimae ATCC 49626
Abiotrophia defectiva ATCC 49176
Actinomyces sp. oral taxon 848 str. F0332
candidate division TM7 single-cell isolate TM7c
Treponema vincentii ATCC 35580
Treponema denticola ATCC 35405
Selenomonas sputigena ATCC 35185
Selenomonas noxia ATCC 43541
Selenomonas artemidis
Prevotella sp. oral taxon 317 str. F0108
Prevotella oris F0302
Prevotella oris C735
Neisseria elongata subsp. glycolytica ATCC 29315
Actinomyces sp. oral taxon 171
Corynebacterium matruchotii ATCC 33806
Capnocytophaga ochracea DSM 7271
Granulicatella elegans ATCC 700633
Catonella morbi ATCC 51271
Fusobacterium nucleatum subsp. polymorphum ATCC 10953
Neisseria mucosa ATCC 25996
Neisseria sp. oral taxon 014 str. F0314
Prevotella sp. oral taxon 472 str. F0295
Rothia dentocariosa ATCC 17931
Rothia dentocariosa M567
Streptococcus gordonii str. Challis substr. CH1
Streptococcus sanguinis SK36
Streptococcus sp. 2_1_36FAA
Actinomyces odontolyticus ATCC 17982
Prevotella melaninogenica D18
Prevotella melaninogenica ATCC 25845
Neisseria subflava NJ9703
Campylobacter concisus 13826
Granulicatella adiacens ATCC 49175
Neisseria flavescens NRL30031/H210
Veillonella parvula DSM 2008
Neisseria flavescens SK114
Capnocytophaga gingivalis ATCC 33624
Capnocytophaga sputigena ATCC 33612 strain Capno
Aggregatibacter aphrophilus NJ8700
Veillonella sp. 6_1_27
Veillonella sp. 3_1_44
Veillonella parvula ATCC 17745
Leptotrichia buccalis DSM 1135
Leptotrichia hofstadii F0254
Neisseria sicca ATCC 29256
Haemophilus influenzae 6P18H1
Haemophilus influenzae R2866
Streptococcus mitis B6
Gemella haemolysans ATCC 10379
Streptococcus mitis ATCC 6249
Streptococcus sp. 73H25AP F0408
Streptococcus sp. M143
Streptococcus oralis ATCC 35037
Campylobacter showae RM3277
Porphyromonas endodontalis ATCC 35406
Fusobacterium periodonticum ATCC 33693
Prevotella tannerae ATCC 51259
Prevotella veroralis F0319
Selenomonas sp. 67H29BP F0410
candidate division TM7 single-cell isolate TM7a
Atopobium parvulum DSM 20469
Solobacterium moorei
Prevotella sp. oral taxon 299 str. F0039
Parvimonas micra ATCC 33270
Megasphaera micronuciformis
Haemophilus influenzae 22.4-21
Fusobacterium sp. 1_1_41FAA
Fusobacterium sp. 2_1_31
Oribacterium sinus F0268
Actinomyces odontolyticus F0309
Veillonella dispar ATCC 17748
Streptococcus thermophilus LMD-9
Streptococcus salivarius SK126
Streptococcus parasanguinis ATCC 15912
Rothia mucilaginosa DY-18
Rothia mucilaginosa ATCC 25296
Corynebacterium accolens ATCC 49725
Corynebacterium accolens ATCC 49726
Corynebacterium pseudogenitalium ATCC 33035
Corynebacterium tuberculostearicum SK141
Moraxella catarrhalis RH4
Prevotella timonensis CRIS 5C-B1
Propionibacterium acnes J139
Propionibacterium acnes J165
Propionibacterium acnes SK137
Propionibacterium acnes SK187
Staphylococcus aureus aureus TCH70
Staphylococcus capitis SK14
Staphylococcus epidermidis BCM-HMP0060
Staphylococcus epidermidis M23864:W2(grey)
Staphylococcus epidermidis RP62A
Staphylococcus epidermidis SK135
Staphylococcus epidermidis W23144
Staphylococcus lugdunensis HKU09-01
Haemophilus influenzae HK1212
Neisseria cinerea ATCC 14685
Streptococcus pneumoniae str. Canada MDR_19A
Streptococcus pneumoniae AP200
Streptococcus pneumoniae TCH8431/19A
Streptococcus pneumoniae str. Canada MDR_19F
