# Supplementary material for: The Prevalence of Species and Strains in the Human Microbiome: A Resource for Experimental Efforts
Source: PLoS One. 2014 May 14;9(5):e97279. doi: 10.1371/journal.pone.0097279 (PMC4020798; doi:10.1371/journal.pone.0097279)
Supplement: Figure S10 — Stable low abundance strains. To find the strains that are present stably at low abundance, strains were identified that differed by only two orders of magnitude over 90% of the subjects (including subjects where the strain was not present) for each of the six body sites. The upper limit of the relative abundance is 0.01% (A), 0.1% (B) and 1% (C), each excluding the strains found for the preceding condition. No stable low abundance strains were identified for anterior nares and posterior fornix. The stable low abundance strains that were identified are visualized in a heat map of the relative abundances with the subjects ordered by the relative abundance for each strain. (PDF) [file pone.0097279.s010.pdf]

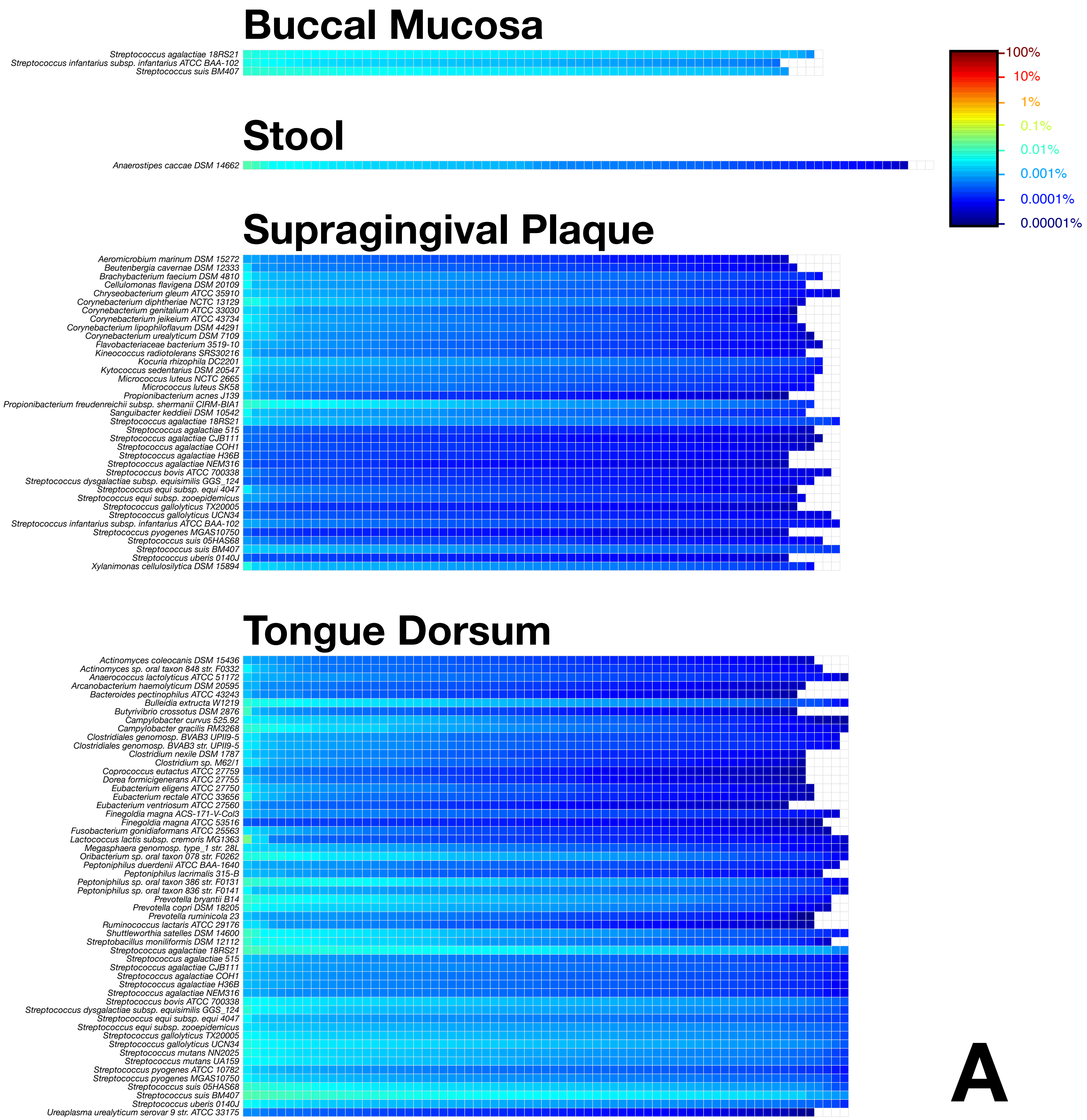
Buccal Mucosa
Streptococcus agalactiae 18RS21
Streptococcus infantarius subsp. infantarius ATCC BAA-102
Streptococcus suis BM407
Stool
Anaerostipes caccae DSM 14662
Supragingival Plaque
Aeromicrobium marinum DSM 15272
Beutenbergia cavernae DSM 12333
Brachybacterium faecium DSM 4810
Cellulomonas flavigena DSM 20109
Chryseobacterium gleum ATCC 35910
Corynebacterium diphtheriae NCTC 13129
Corynebacterium genitalium ATCC 33030
Corynebacterium jeikeium ATCC 43734
Corynebacterium lipophiloflavum DSM 44291
Corynebacterium urealyticum DSM 7109
Flavobacteriaceae bacterium 3519-10
Kineococcus radiotolerans SRS30216
Kocuria rhizophila DC2201
Kytococcus sedentarius DSM 20547
Micrococcus luteus NCTC 2665
Micrococcus luteus SK58
Propionibacterium acnes J139
Propionibacterium freudenreichii subsp. shermanii CIRM-BIA1
Sanguibacter keddieii DSM 10542
Streptococcus agalactiae 18RS21
Streptococcus agalactiae 515
Streptococcus agalactiae CJB111
Streptococcus agalactiae COH1
Streptococcus agalactiae H36B
Streptococcus agalactiae NEM316
Streptococcus bovis ATCC 700338
Streptococcus dysgalactiae subsp. equisimilis GGS_124
Streptococcus equi subsp. equi 4047
Streptococcus equi subsp. zooepidemicus
Streptococcus gallolyticus TX20005
Streptococcus gallolyticus UCN34
Streptococcus infantarius subsp. infantarius ATCC BAA-102
Streptococcus pyogenes MGAS10750
Streptococcus suis 05HAS68
Streptococcus suis BM407
Streptococcus uberis 0140J
Xylanimonas cellulosilytica DSM 15894
Tongue Dorsum
Actinomyces coleocanis DSM 15436
Actinomyces sp. oral taxon 848 str. F0332
Anaerococcus lactolyticus ATCC 51172
Arcanobacterium haemolyticum DSM 20595
Bacteroides pectinophilus ATCC 43243
Bulleidia extructa W1219
Butyrivibrio crossotus DSM 2876
Campylobacter curvus 525.92
Campylobacter gracilis RM3268
Clostridiales genomosp. BVAB3 UPII9-5
Clostridiales genomosp. BVAB3 str. UPII9-5
Clostridium nexile DSM 1787
Clostridium sp. M62/1
Coprococcus eutactus ATCC 27759
Dorea formicigenerans ATCC 27755
Eubacterium eligens ATCC 27750
Eubacterium rectale ATCC 33656
Eubacterium ventriosum ATCC 27560
Finegoldia magna ACS-171-V-Col3
Finegoldia magna ATCC 53516
Fusobacterium gonidiaformans ATCC 25563
Lactococcus lactis subsp. cremoris MG1363
Megasphaera genomosp. type_1 str. 28L
Oribacterium sp. oral taxon 078 str. F0262
Peptoniphilus duerdenii ATCC BAA-1640
Peptoniphilus lacrimalis 315-B
Peptoniphilus sp. oral taxon 386 str. F0131
Peptoniphilus sp. oral taxon 836 str. F0141
Prevotella bryantii B14
Prevotella copri DSM 18205
Prevotella ruminicola 23
Ruminococcus lactaris ATCC 29176
Shuttleworthia satelles DSM 14600
Streptobacillus moniliformis DSM 12112
Streptococcus agalactiae 18RS21
Streptococcus agalactiae 515
Streptococcus agalactiae CJB111
Streptococcus agalactiae COH1
Streptococcus agalactiae H36B
Streptococcus agalactiae NEM316
Streptococcus bovis ATCC 700338
Streptococcus dysgalactiae subsp. equisimilis GGS_124
Streptococcus equi subsp. equi 4047
Streptococcus equi subsp. zooepidemicus
Streptococcus gallolyticus TX20005
Streptococcus gallolyticus UCN34
Streptococcus mutans NN2025
Streptococcus mutans UA159
Streptococcus pyogenes ATCC 10782
Streptococcus pyogenes MGAS10750
Streptococcus suis 05HAS68
Streptococcus suis BM407
Streptococcus uberis 0140J
Ureaplasma urealyticum serovar 9 str. ATCC 33175
100%
10%
1%
0.1%
0.01%
0.001%
0.0001%
0.00001%
A

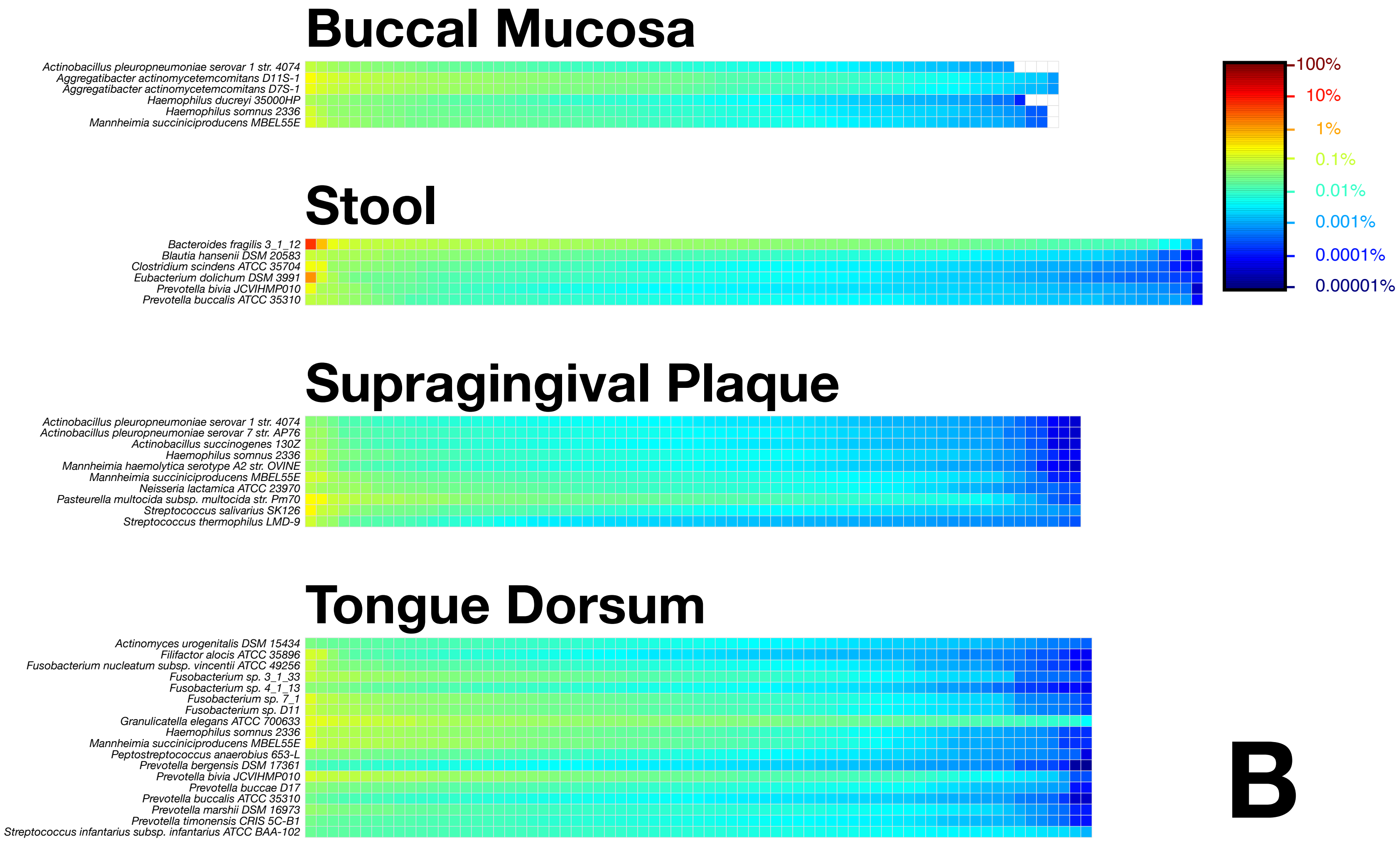
Buccal Mucosa
Actinobacillus pleuropneumoniae serovar 1 str. 4074
Aggregatibacter actinomycetemcomitans D11S-1
Aggregatibacter actinomycetemcomitans D7S-1
Haemophilus ducreyi 35000HP
Haemophilus somnus 2336
Mannheimia succiniciproducens MBEL55E
Stool
Bacteroides fragilis 3_1_12
Blautia hansenii DSM 20583
Clostridium scindens ATCC 35704
Eubacterium dolichum DSM 3991
Prevotella bivia JCVIHMP010
Prevotella buccalis ATCC 35310
Supragingival Plaque
Actinobacillus pleuropneumoniae serovar 1 str. 4074
Actinobacillus pleuropneumoniae serovar 7 str. AP76
Actinobacillus succinogenes 130Z
Haemophilus somnus 2336
Mannheimia haemolytica serotype A2 str. OVINE
Mannheimia succiniciproducens MBEL55E
Neisseria lactamica ATCC 23970
Pasteurella multocida subsp. multocida str. Pm70
Streptococcus salivarius SK126
Streptococcus thermophilus LMD-9
Tongue Dorsum
Actinomyces urogenitalis DSM 15434
Filifactor alocis ATCC 35896
Fusobacterium nucleatum subsp. vincentii ATCC 49256
Fusobacterium sp. 3_1_33
Fusobacterium sp. 4_1_13
Fusobacterium sp. 7_1
Fusobacterium sp. D11
Granulicatella elegans ATCC 700633
Haemophilus somnus 2336
Mannheimia succiniciproducens MBEL55E
Peptostreptococcus anaerobius 653-L
Prevotella bergensis DSM 17361
Prevotella bivia JCVIHMP010
Prevotella buccae D17
Prevotella buccalis ATCC 35310
Prevotella marshii DSM 16973
Prevotella timonensis CRIS 5C-B1
Streptococcus infantarius subsp. infantarius ATCC BAA-102
100%
10%
1%
0.1%
0.01%
0.001%
0.0001%
0.00001%
B

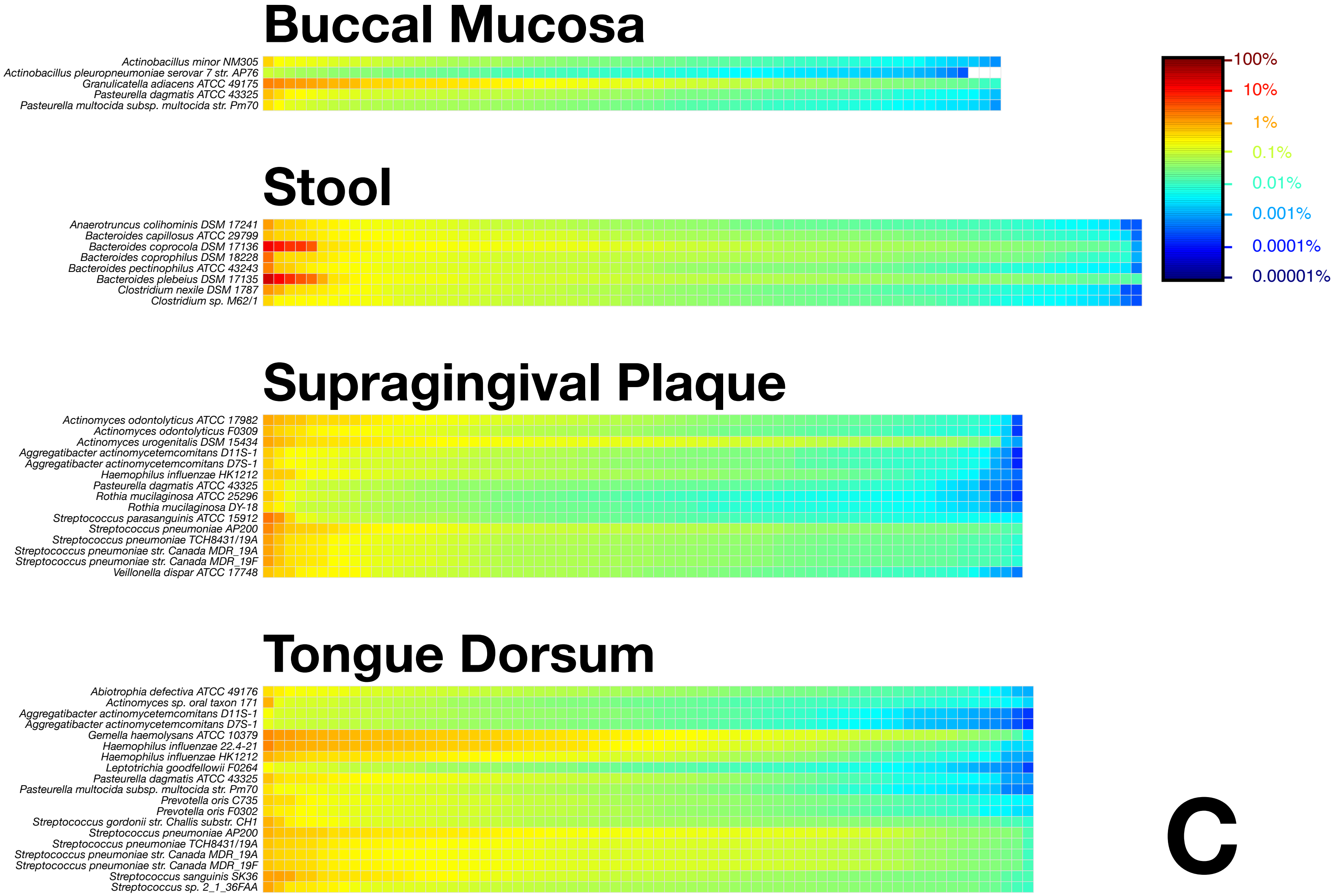
Buccal Mucosa
Actinobacillus minor NM305
Actinobacillus pleuropneumoniae serovar 7 str. AP76
Granulicatella adiacens ATCC 49175
Pasteurella dagmatis ATCC 43325
Pasteurella multocida subsp. multocida str. Pm70
Stool
Anaerotruncus colihominis DSM 17241
Bacteroides capillosus ATCC 29799
Bacteroides coprocola DSM 17136
Bacteroides coprophilus DSM 18228
Bacteroides pectinophilus ATCC 43243
Bacteroides plebeius DSM 17135
Clostridium nexile DSM 1787
Clostridium sp. M62/1
Supragingival Plaque
Actinomyces odontolyticus ATCC 17982
Actinomyces odontolyticus F0309
Actinomyces urogenitalis DSM 15434
Aggregatibacter actinomycetemcomitans D11S-1
Aggregatibacter actinomycetemcomitans D7S-1
Haemophilus influenzae HK1212
Pasteurella dagmatis ATCC 43325
Rothia mucilaginosa ATCC 25296
Rothia mucilaginosa DY-18
Streptococcus parasanguinis ATCC 15912
Streptococcus pneumoniae AP200
Streptococcus pneumoniae TCH8431/19A
Streptococcus pneumoniae str. Canada MDR_19A
Streptococcus pneumoniae str. Canada MDR_19F
Veillonella dispar ATCC 17748
Tongue Dorsum
Abiotrophia defectiva ATCC 49176
Actinomyces sp. oral taxon 171
Aggregatibacter actinomycetemcomitans D11S-1
Aggregatibacter actinomycetemcomitans D7S-1
Gemella haemolysans ATCC 10379
Haemophilus influenzae 22.4-21
Haemophilus influenzae HK1212
Leptotrichia goodfellowii F0264
Pasteurella dagmatis ATCC 43325
Pasteurella multocida subsp. multocida str. Pm70
Prevotella oris C735
Prevotella oris F0302
Streptococcus gordonii str. Challis substr. CH1
Streptococcus pneumoniae AP200
Streptococcus pneumoniae TCH8431/19A
Streptococcus pneumoniae str. Canada MDR_19A
Streptococcus pneumoniae str. Canada MDR_19F
Streptococcus sanguinis SK36
Streptococcus sp. 2_1_36FAA
100%
10%
1%
0.1%
0.01%
0.001%
0.0001%
0.00001%
C
